# How eriophyid mites shape metal metabolism in leaf galls on *Tilia cordata*

**DOI:** 10.1111/nph.70103

**Published:** 2025-04-16

**Authors:** Filis Morina, Anđela Kuvelja, Dennis Brückner, Miloš Mojović, Đura Nakarada, Syed Nadeem Hussain Bokhari, Bojan Vujić, Gerald Falkenberg, Hendrik Küpper

**Affiliations:** ^1^ Czech Academy of Sciences, Biology Centre, Institute of Plant Molecular Biology Laboratory of Plant Biophysics & Biochemistry 370 05 České Budějovice Czech Republic; ^2^ University of South Bohemia Faculty of Science 370 05 České Budějovice Czech Republic; ^3^ Deutsches Elektronen‐Synchrotron DESY 22607 Hamburg Germany; ^4^ Faculty of Physical Chemistry University of Belgrade 11000 Belgrade Serbia

**Keywords:** biotic stress, eriophyid mites, metal metabolism, micro‐XANES tomography, micro‐XRF tomography, Mn speciation, nutritive tissue, *Tilia cordata* galls

## Abstract

- Metal metabolism in plant–galler interactions is largely unknown. We hypothesise that the mites manipulate metal distribution by sequestration of excessive levels and differential regulation of metalloproteins to support the main functions of gall‐nutrition, protection and microenvironment.
- Using the *Tilia cordata*–eriophyid mites system, we aimed to reveal the role of metals in galls by investigating their distribution, speciation, gene expression and metabolome profiling. Complementary spectroscopy techniques (μXRF and μXANES tomographies, electron paramagnetic resonance), histochemical, metabolomic and transcriptomic analyses were employed.
- Mn was the most abundant micronutrient in the galls. Differential cell‐specific Mn accumulation (idioblasts vs nutritive tissue) and speciation are essential for its homeostasis. Mn(II)‐aquo complex, co‐localised with Ca, sequestered in idioblasts, while Mn bound to stronger ligands including enzymes accumulated in the nutritive tissue. Zn, Cu and Fe predominately accumulated in the nutritive tissue to support intensive metabolic processes such as secondary and lipid metabolism, protein N‐glycosylation and redox regulation. The slower rate of redox‐sensitive spin probes' decay in the galls indicated a lower amount of antioxidants than in the leaf.
- We reveal essential functions of micronutrients in the galls, supporting the developmental and chemical changes in the host plant, and the nutrition of the galler.

## Introduction

Galls are remarkable examples of biochemical, physiological and morphological changes in plant organs induced by various organisms, including bacteria, fungi, nematodes and arthropods (Mani, 2013; Ferreira *et al*., 2019). The mechanisms of gall induction and development, especially those induced by arthropods, are not well understood, and many questions remain open (reviewed by Harris & Pitzschke, 2020). The evolutionary advantage of producing galls can be explained by three main hypotheses: the nutritional hypothesis stating that galls provide high‐quality nutrient‐rich tissue available directly to the galler over the whole associated life cycle; the protection (enemy) hypothesis stating that galls provide a safe place against biotic stress such as predators and pathogens; and the microenvironment hypothesis stating that galls protect the gallers from abiotic stress (temperature, UV radiation, etc.) allowing optimal conditions for reproduction and growth (Price *et al*., 1987; Stone & Schönrogge, 2003; Harris & Pitzschke, 2020).

Arthropod‐induced galls can have different anatomical features, from simple tissue swelling, to complex, fascinating neoformed structures (Larew, 1981; Mani, 2013; Ferreira *et al*., 2019). Most galling‐inducing arthropods are highly host‐specific and often limited to one tissue type, such as leaf bud, stem or roots (Rohfritsch, 1992). Galls are induced by active compounds, cecidogens, excreted during feeding (saliva) or oviposition (Raman, 2011). Although the exact mechanisms of gall initiation and development are not fully known, some active molecules have been identified, including hormones, effector proteins and small RNAs (Little *et al*., 2007; Petanović & Kielkiewicz, 2010a; Medina *et al*., 2017; Harris & Pitzschke, 2020). Accumulation of growth‐regulating hormones contributes to morphological changes (cell hypertrophy and tissue hyperplasia) during gall development (Petanović & Kielkiewicz, 2010b; Giron *et al*., 2016; Oliveira *et al*., 2016; Harris & Pitzschke, 2020).

The chemical composition of the galls differs from the surrounding host tissue, and it is manipulated to benefit the galler. Most of the studies on gall chemical modifications investigated the accumulation and distribution of primary and secondary metabolites. Galls developing on photosynthetically active tissues act as newly formed sinks, with inhibited photosynthetic activity but with the active import of photoassimilates (Zorić *et al*., 2019; Jiang *et al*., 2021). Tissue‐specific accumulation of phenolic compounds has been observed as well, mostly in the outer layers of the galls with decreased content in the nutritive tissue, as well as increased lignification (in line with their antioxidative properties and structural support, respectively; Nyman & Julkunen‐Tiitto, 2000; Guedes *et al*., 2019).

However, although essential for all organisms, the metabolism of macro‐ and micronutrients in plant–galler interactions remains underexplored. Transition metals (Fe, Cu, Zn, Ni, Mn and Mo) are required for plant (and arthropod) development and survival. They have a broad range of functions in overall metabolism, including gene regulation, post‐translational protein modifications, enzyme activation and redox reactions (Andresen *et al*., 2018; He *et al*., 2021; Arriola *et al*., 2024). Among the available literature, the majority is related to measuring the total metal concentrations in the galls (e.g. Bagatto & Shorthouse, 1991, 1994a; Arriola *et al*., 2024). Mineral distribution in insect galls has been demonstrated in only a few studies so far, not quantitatively because of the methods used, and without deeper insight into their functional role, transport or binding (Bagatto & Shorthouse, 1994b; Anand & Ramani, 2021).

Highly specialised mites (genera from families Phytoptidae and Eriophyidae) can induce galls in different organs – leaves, buds, stems or fruits – by feeding (Chetverikov *et al*., 2015). These mites are economically important pests of crops (apple, pear, walnut, cherries, maple and grapevine), grasses and ornamental crops, including plants in urban ecosystems such as linden, poplar, willow, ash, alder and elm. They can considerably affect crop physiology and production (De Lillo *et al*., 2018; Jiang *et al*., 2021). The type of mite leaf galls varies from erinea (felt‐like masses due to abnormal hair development), blister galls (pocket or warty galls), to marginal leaf roll galls, vein galls and nail (pouch) galls (Westphal & Manson, 1996). The mechanisms of eriophyid mite gall formation and their interaction with the host are not well documented. It is known that gall initiation is triggered by the saliva of overwintering female eriophyid mites (Petanović & Kielkiewicz, 2010a,b; Chetverikov *et al*., 2015). These deutogyne females overwinter beneath the bud scales. They start feeding and inducing galls for laying eggs in emerging leaves in early spring. Inside the galls, several generations of male and female mites are present during summer, but in early autumn overwintering females are produced, which will not lay eggs until the spring of the following year. Early mite gall development includes intense proliferation of epidermal cells and de‐differentiation of cells from parenchyma to meristematic cells. With gall maturation the proliferation decreases, and lignification occurs (Petanović & Kielkiewicz, 2010a,b; Chetverikov *et al*., 2015).

Recent reports show that trace metals (e.g. Zn, Fe) are essential in plant–pathogen interactions. The mechanisms of metal‐based immunity involve metalloproteins, low molecular weight (LMW) ligands and phytohormone accumulation (Morina *et al*., 2021; Morina & Küpper, 2022; Kuvelja *et al*., 2024). We hypothesise that micronutrients have an important role in plant response to the galler and gall development and that micronutrients have differential tissue distribution in the galls related to their function to accommodate the needs of the mites. This is different from the defence response to pathogens because gallers can hijack the plant proteasome, minimise/manipulate the defence responses of the host and suppress the immune system (Ithal *et al*., 2007; Tooker *et al*., 2008).

## Materials and Methods

### Plant material and soil sampling

Eighteen *Tilia cordata* Mill. trees were used to collect samples of healthy (HL) and infested leaves (INFL) with nail galls (G) in the period 2019–2023. The galls were produced by *Eriophyes tiliae* (Pgst. 1857). The trees were located in the town of České Budějovice, Czech Republic (48.997373, 14.452757; 48.993876, 14.453670; 48.979251, 14.448508; 48.972160, 14.473485; and 48.986123, 14.437683; Supporting Information Fig. S1). The gall initiation period started around the end of April to the middle of May every year. The healthy and infested leaves with mature galls were collected in June–July every year, placed in plastic dishes with moist paper towels to retain freshness and immediately transferred to the lab. Both, healthy and infested leaves were taken from the same, light‐exposed branches, at *c*. 2 m aboveground (lower part of the tree crown). The leaves were thoroughly rinsed with ddH_2_O to remove dust from both leaf sides and tap‐dried with cleanroom wipes before further handling. Intact leaves with galls were used for *in vivo* analyses, and for all other analyses the galls were separated from the leaves.

Soil samples were collected adjacent to the trees at three representative locations from the topsoil at 0–40 cm depth with 10 cm fragments. The soil samples were dried at 60°C for 1 d, then air‐dried for 4 d and sieved through a 2‐mm sieve before further processing. Soil pH was measured in a 1 : 1 w/v suspension in ddH_2_0. The pH was, on average, lower in locations 1 and 2 (pH 5.9 ± 0.2 and 5.8 ± 0.9, respectively) and the highest in location 3 (7.4 ± 0.3).

### Bulk element content in plants and soil

The acid digests and analyses of bulk element content in the plants and soil are described in Methods S1.

### Light microscopy and transmission electron microscopy analyses

For semi‐thin sections and transmission electron microscopy (TEM) analyses, young galls (Stage 2), mature galls (Stage 4) and healthy leaf tissue (samples from infested leaves from four individual trees) were fixed in 2.5% glutaraldehyde in 0.1 M phosphate buffer, 4% sucrose, pH 7.2 for 24 h at 4°C. After washing three times in 0.1 M phosphate buffer, the samples were postfixed with 2% osmium tetroxide for 2 h, dehydrated in an acetone series and embedded in Spurr resin (EMS, Hatfield, PA, USA). Semi‐thin sections were stained with toluidine blue and observed with an OPTIKA microscope equipped with a PRO8 Digital Camera C‐P8 (Optika, Ponteranica, Italy). Ultra‐thin (90 nm) sections were stained with uranyl acetate and lead citrate and examined in a TEM JEOL 1400 (JEOL Ltd, Tokyo, Japan) at 120 kV.

Proanthocyanidins and lipids in the mature galls were visualised after staining fresh hand‐sectioned galls from six different trees. Vanillin‐HCl staining was used to visualise proanthocyanidins according to Gardner (1975). Sudan Black B (in 70% ethanol) was used to detect lipids, and ruthenium red was used to detect acidic mucilages and pectins (Demarco, 2017). The sample preparation is described in detail in Methods S2. The ruthenium red sections were visualised using a LEICA DM IRB inverted microscope (Leica Microsystems, Wetzlar, Germany) equipped with an ILCE‐7M2 camera (Sony, Tokyo, Japan). Vanillin‐HCl and Sudan Black stained sections were observed using an OPTIKA microscope with the Optikam PRO8 Digital Camera C‐P8 (Optika, Ponteranica, Italy).

### Sample preparation and analyses by benchtop micro‐XRF (micro X‐ray fluorescence) and fast Chl fluorescence kinetics (OJIP)

For *in vivo* analyses of metal distribution in the leaves and galls (mature and young), infested leaves with nail galls at two developmental stages were analysed by benchtop μXRF (Tornado; Bruker Nano GmbH, Berlin, Germany) as described in Mijovilovich *et al*. (2020) in three seasons, 2020, 2022 and 2024 (eight different trees). The samples were mounted in the measuring chamber, covered with a slightly moistened cotton pad, fixed with a mesh and aerated by water‐saturated air using a pump. Before μXRF, the leaves with mature galls and healthy leaves were used for OJIP analyses as previously described by Küpper *et al*. (2019) using the Fluorcam 7 software (PSI, Drasov, Czech Republic).

### Synchrotron micro‐XRF and XANES

The synchrotron‐based μXRF tomography of K, Ca, Mn, Fe, Ni, Cu and Zn distribution in the galls and leaves, as well as micro X‐ray absorption near edge structure (μXANES) tomography, was performed at the DESY PETRA III beamline P06. Additional bulk‐level XANES spectra were recorded at the EXAFS beamline P65 at DESY.

#### Sample preparation

Three samples of nail galls and three leaves from three different trees collected in 2020 and 2021 were measured for (sub)cellular distribution. The galls were glued on top of 1 mm diameter polyimide capillaries. The leaves were cut excluding the main vein with a sharp blade and mounted in 10% glycerol in the same type of 1 mm diameter polyimide capillaries. The following standards were prepared: MnCl_2_ in water as Mn(II)‐aquo; Mn(III) and Mn(IV) dry powder were mixed separately with polyvinylpolypyrrolidone (PVPP) to a final concentration of 1000 ppm. Soluble complexes with LMW ligands were prepared in a 1 : 10, metal : ligand ratio using 5 mM MnCl_2_ with the following ligands: malate (pH 5); phytate (pH 5); nicotianamine (pH 7); and reduced glutathione (pH 7). The pH was adjusted either to 5 with 0.6 M MES‐KOH or to 7 with 0.6 M HEPES‐KOH. A thylakoid standard for Mn in an oxygen‐evolving complex (OEC) was prepared according to Chen *et al*. (2016) from fresh *T. cordata* leaves. To obtain the mucilage, fresh *T. cordata* leaves were homogenised in liquid N_2_, mixed with ddH_2_O and incubated in a shaker for 24 h at 50°C. The mucilage extract was filtered through a mesh and centrifuged at 10 000 ***g*** for 30 min (similarly as in Koc‐Bilican, 2024). The supernatant was incubated with 5 mM MnCl_2_ for 24 h and used as a standard. Cell wall samples were prepared according to Vojvodić *et al*. (2020) from fresh *T. cordata* leaves. The standards for bulk XANES measurement at P65 were prepared in cuvettes with polyimide windows, and those for μXANES measurement at P06 were prepared in 1 mm diameter polyimide capillaries. All standards except the Mn(III) and Mn(IV) dry powder were prepared with 10% glycerol. All standards and all plant samples were manually shock‐frozen in supercooled isopentane at −140°C to minimise the formation of ice crystals and de‐mixing during freezing (Mishra *et al*., 2013), and stored in liquid N_2_.

#### Development of an improved sample environment for μXRF/μXANES cryo‐tomography

**Fig. 1:**
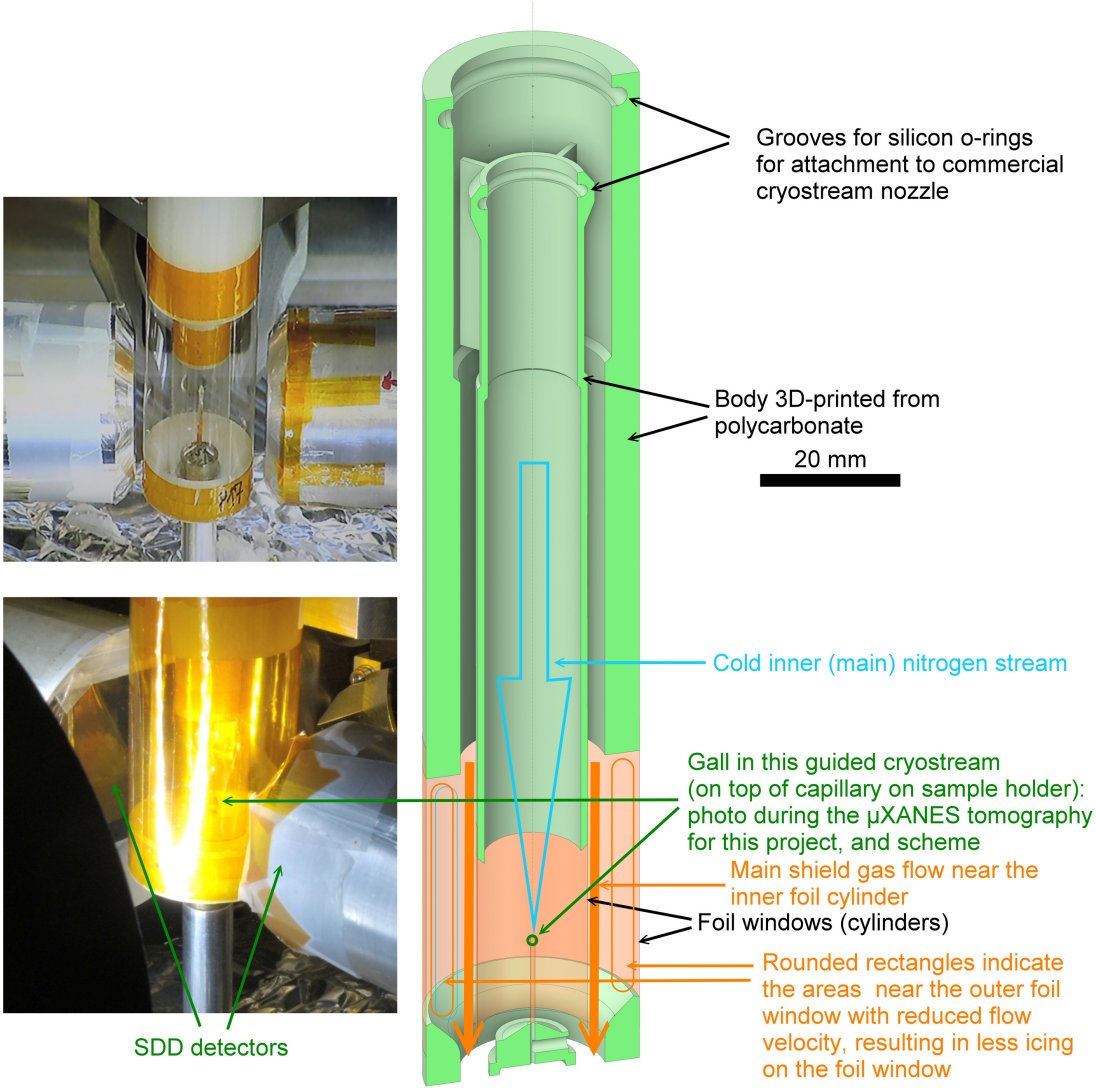
Photo and scheme of the guided cryostream developed to eliminate problems of vibration and icing during long μXANES and μXRF tomography measurements. Left: photos of the final version (upper) and the one used for the μXANES beamtime lower). For the final version, the polyimide (Kapton®) foil windows were replaced by Flexio 220175 foil for better visibility of the sample and to reduce X‐ray absorption, in particular for Cu that is always present as contamination in polyimide. Right: scheme showing the details of the internal structure of the cryostream guide. The foil windows are glued to the body (also connecting the upper and lower part of the body) after 3D printing by Kapton® tape.

For the μXRF and μXANES tomography at P06, a new sample environment was developed to minimise the vibrations and icing that had previously caused problems with long μXANES and μXRF tomography measurements (Mijovilovich *et al*., 2019). This sample environment, named ‘guided cryostream’, was attached to the end of a commercial cryostream (here: Oxford Cryosystem 700) and is based on two cylinders of polycarbonate with windows made of clear plastic foil (Flexio 220175, Flexio as. Prague, Czechia) where the actual cryostream flows in the inner cylinder and the guard stream in the outer cylinder (Fig. 1). The shape of the cylinders was designed such that the flow inside is laminar, but in the section with windows a stagnant gas layer is formed next to the window. This prevented the temperature of the outer window from falling below the condensation point, thus preventing the condensation of water at the height of the sample. In this way, measurements with no observable vibration and thermal drift < 1 μm became possible, allowing for the high‐resolution μXRF and μXANES tomograms shown here.

The bulk XANES at P65 (most of the standards) was measured at 20 K in a closed‐cycle helium cryostat.

#### μXRF and XANES measurements and data acquisition

This is described in Methods S3.

#### XANES data analysis

In each μXANES tomography of the biological samples, 4–12 representative cells were chosen in the μXANES tomograms and their XANES spectra were individually exported. This was done in the Fiji version of ImageJ (1.54f). Data reduction of raw XANES data was done in the athena software (Ravel & Newville, 2005), in a strictly consistent way to make it traceable and free of bias despite the different data sources (P06 vs P65). All spectra were cropped to the available consensus energy range (6520–6734 eV), a linear fit of the pre‐edge background from 30 to 15 eV before the edge was combined with a 2^nd^ order polynomial fit of the post‐edge background between 85 and 185 eV above the edge. After data reduction including normalisation, all spectra of the same tissue type and biological replicate were averaged. Then, linear combination fitting was performed in SigmaPlot 14 (Systat Software Inc., San Jose, CA, USA), first with all available standards to prevent bias by a too‐narrow choice, and then only with those models that were > 0 in the first fit for at least one biological sample. Standard errors in the data are from three independent biological replicates (separately collected galls).

#### μXRF data analysis

In principle, the procedure described by Mishra *et al*. (2013) was followed for absorption correction and full quantification of the tomograms. However, the not perfectly round shape of the galls and the fact that they are hollow forced a modification of the procedure, which was also further optimised for ease of use, and is therefore described in detail in Methods S4.

### Electron paramagnetic resonance measurements

All electron paramagnetic resonance (EPR) measurements were performed using a Bruker ELEXSYS‐II X/L spectrometer (Rheinstetten, Germany) with an R4123SHQE X‐band resonator. The EPR spectra were analysed using Xepr software (Bruker BioSpin GmbH, Rheinstetten, Germany). Redox probes 3CP (3‐Carbamoyl‐2,2,5,5‐tetramethyl‐1‐pyrrolidinyl‐*N*‐oxyl), and 3CxP (3‐Carboxy‐2,2,5,5‐tetramethyl‐pyrrolidinyl‐*N*‐oxyl) were purchased from Sigma‐Aldrich (Steinheim, Germany), and spin trap DEPMPO (5‐(Diethoxyphosphoryl)‐5‐methyl‐1‐pyrroline‐N‐oxide) was purchased from Focus Biomolecules (Plymouth Meeting, PA, USA). Lignin (dealkaline) was purchased from TCI (USA).

#### Mn^2+^ detection in intact tissues

Healthy (HL) and infested leaves (INFL) with nail galls (G) were collected from three trees and immediately transferred to the lab under moist conditions. After rinsing with ddH_2_O, sections of each tissue type were placed on a quartz tissue cell and fixed with a quartz glass. The following conditions were used for Mn^2+^ determination: field centre at 3490 G; sweep width of 4300 G; microwave power at 10 mW; microwave frequency of 9.8 GHz; modulation frequency of 100 kHz; and modulation amplitude of 5 G. The following standards were measured in gas‐permeable Teflon tubes (Zeus Industries, Letterkenny, Ireland) under the same conditions (20 μl): MnSOD dissolved in 0.6 mM HEPES buffer pH 7 (1 mg ml^−1^); an aqueous solution of 5 mM MnCl_2_; and a mixture of MnCl_2_ and ethylenediaminetetraacetic acid (EDTA) in water was prepared in a 1 : 10 ratio and dried to simulate the bound Mn^2+^ signal. For quantitative analyses of the ratio of the different types of Mn^2+^ binding, the peak heights of the first and sixth Mn^2+^ peaks were taken, as well as the height of the starting and end points of the broad Mn^2+^ peak. The intensities of the spectra were normalised to fresh weight after conversion in Xepr software (Bruker).

#### Measurements of reactive oxygen species (ROS) generation in isolated cell walls using spin trap DEPMPO

Cell walls (CWs) from HL, INFL, and nail galls (three biological replicates – different trees) were isolated according to Morina *et al*. (2010). For the measurements, isolated CWs were placed on a tissue cell and measured before and after the addition of 10 μl of 100 mM DEPMPO. The spectra were recorded after 20 accumulations. Computer simulations were done in SpinFit (Bruker BioSpin). The quantitative analyses were done after normalisation to dry weight (after drying the pellets for 48 h at 70°C).

#### 2D (imaging) real‐time detection of the capacity to reduce extra‐ and intracellular pyrrolidine spin probes

To reveal physiologically relevant dynamics of leaf and gall redox status, and to avoid risks of extraction artefacts, spatiotemporal visualisation was performed. This was achieved by measuring the capacity of the intact leaves and nail galls to reduce pyrrolidine spin probes: the neutral, membrane‐permeable 3CP and negatively charged membrane‐impermeable 3CxP. The intact galls and leaf sections were infiltrated in 80 mM 3CP or 80 mM 3 CxP and three time points were chosen to follow the dynamics of 3CP and 3CxP reduction. Three biological replicates of galls and leaves were measured for both probes. The EPR signals were measured under the following conditions similarly as described by Milutinović *et al*. (2023): microwave power, 10 mW; microwave frequency, 9.8 GHz; modulation frequency, 100 kHz; modulation amplitude, 2 G; magnetic field gradient, 20 G cm^−1^. The 2D images were recorded at three intervals (21, 45 and 75 min for 3CP; 21, 45 and 95 min for 3CxP).

### *De novo* transcriptome assembly

For total mRNA sequencing, RNA was extracted with the CTAB method (Chang *et al*., 1993) from three tissue types (healthy leaves, infested leaves and galls) collected from four trees. Subsequently, the RNA was treated with DNase and RNeasy® MinElute clean‐up kit (both Qiagen). The RNA purity was determined spectrophotometrically via absorption ratios at 260/280 nm and 260/230 nm measured using Traycell cuvettes for ‘nanodrop’ measurement (Hellma, Müllheim, Germany) and Lambda 750 spectrometer (Perkin‐Elmer, Waltham, MA, USA). Integrity was verified by gel electrophoresis. RNA sequencing and differential gene expression (GE) analyses were done by Novogene (Novogene Co., European branch: Cambridge, UK). Sequencing libraries were prepared from 1 μg of RNA per sample (12 in total) using the Illumina HiSeq platform (150‐bp paired‐end reads). High‐quality reads were selected via fragment filtering with a base call accuracy of 99.9% (Q30). All analyses from assembly, gene functional annotation, mapping and quantification, differential expression analysis and Gene Ontology (GO) and Kyoto Encyclopedia of Genes and Genomes (KEGG) enrichments were done by the Novogene Co. as explained in detail in Notes S1.

### Metabolomics

Untargeted and targeted metabolomic analyses (liquid chromatography mass spectrometry, lipidomics and gas chromatography mass spectrometry) were done using three tissue types (healthy leaves, infested leaves and galls) collected from four trees. Extraction of metabolites and analyses were done by MSOmics metabolomic facilities at the University of Copenhagen, Denmark. Metabolites were extracted from *c*. 100 mg of frozen tissues and the details are described in Notes S2. In the analyses, metabolites that were not of plant origin were excluded, while the possible contribution of the mites to the metabolomic pool was considered minimal due to a much lower weight (abundance) compared to the plant material.

### Statistical analyses

Comparison of groups for finding statistically significant differences regarding the leaf tissues (HL, INFL, G) and bulk element content, and for photosynthetic parameters, was done using the Mann–Whitney *U* test method in OriginPRO (v.2019b; Originlab, Northampton, MA, USA). Statistics of the *de novo* transcriptomic analyses and quality control were part of the RNA‐seq approach with the Benjamini and Hochberg correction for *P*‐values. Metabolomics data were analysed with the Wilcoxon test using viime software (Choudhury *et al*., 2020). Statistical analyses of XANES data were done in SigmaPlot (v.14; Grafiti LLC, Palo Alto, USA) following Mishra *et al*. (2013). Briefly, a set of model compounds was fitted to the sample spectra as a linear combination, with linear correction for baseline drift. This yielded proportions of each ligand, with SE for each fit. To see the variability between biological replicates (galls from different trees, *n* = 3), SE was also calculated from the results of these replicates; this is the SE shown in the graphs.

## Results

### Bulk element contents in leaves, galls and soil

The analyses of total metal concentrations in leaves and galls showed large variability in the Mn, Zn, Cu and Fe content among individual trees (Fig. S2). Mn concentrations were the highest among the micronutrients, ranging from 30 to 800 mg kg^−1^, Fe from 12 to 110 mg kg^−1^, Cu from 1 to 14 mg kg^−1^ and Zn from 4 to 28 mg kg^−1^. The fold‐change in metal concentrations for each tree between the three tissue types showed a higher accumulation of P and K in the galls (Fig. S3). The analyses of bioavailable (ammonium acetate‐extractable) metal content in the soil adjacent to the infested trees showed that Mn concentrations varied up to 10‐fold, while the variability of Cu, Fe and Zn was lower (Table S1).

### Leaf and gall morphology and ultrastructure

**Fig. 2:**
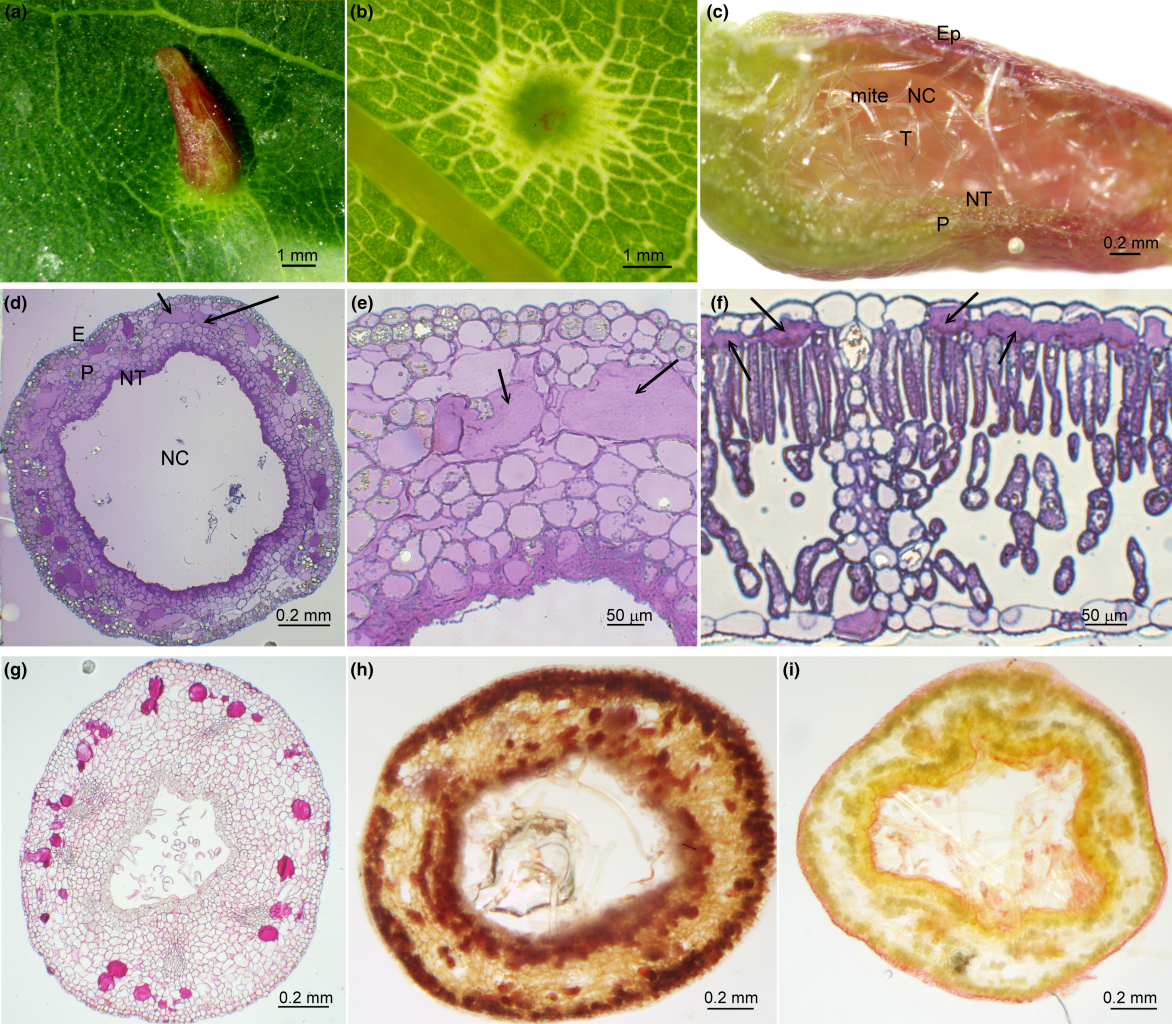
Light microscopy images of a mature nail gall induced by eriophyid mites on *Tilia cordata* leaves. (a) Abaxial, (b) adaxial leaf side, (c) longitudinal section of a typical gall showing the nutritive chamber (NC), nutritive tissue surrounding the chamber (NT), parenchyma (P), epidermis (Ep), trichomes in the nutritive camber (T) and a mite inside the chamber. Cross‐sections stained with toluidine blue; arrows mark idioblasts. (d) Nail gall with ×10 magnification. (e) Enlarged section of the previous gall image. (f) Leaf section showing idioblasts just below the epidermal cells. (g–i) Histochemical analyses: (g) detection of pectin/acidic mucilage by ruthenium red; (h) detection of proanthocyanidins by vanillin‐HCl; (i) detection of lipids by Sudan Black.

The macroscopic morphology of a typical nail gall is shown in Fig. 2(a–c). The feeding site and gall initiation occur on the adaxial leaf side (Fig. 2b). A longitudinal gall section (Fig. 2c) shows the larval chamber in the middle filled with trichomes, the nutritive tissue lining the chamber, parenchyma cells further from the chamber and the epidermal tissue. Semi‐thin sections of mature galls (Fig. 2d,e) compared to a healthy leaf (Fig. 2f) showed that the secretory cells (idioblasts), which are directly below the epidermis in the leaf, move several layers below the epidermis in the gall and become much larger. Further, a lining of the nutritive tissue develops surrounding the chamber, while the mesophyll degenerates.

### Histochemical characterisation of gall idioblasts – staining for mucilage, lipids and proanthocyanidins in the galls

To reveal the content of secretory cells, mature galls were stained to detect acidic mucilage and pectin with ruthenium red, vanillin‐HCl for proanthocyanidins and Sudan Black for lipids (Fig. 2g). Large secretory cells (idioblasts) were found to be filled with mucilage/pectin visible as a strong pink colouration. In addition, the cell walls and several other smaller cells were stained but to a much lower intensity. Proanthocyanidins accumulated mostly in the epidermis and outer parenchyma forming a ring and in the cell layers close to the larval chamber lining (Fig. 2h). Lipids were also detected in the epidermis and the nutritive tissue lining the larval chamber (Fig. 2i).

Ultrastructural analyses of *T. cordata* mature galls by TEM showed that idioblasts have a denser content than the surrounding cells (Fig. S4). At higher magnification, the micellar structure of idioblasts was observed (Fig. S4K). Parenchyma cells had numerous tannin deposits visible as black circles, while in several gall sections, raphide crystals were present. In the nutritive tissue, cells with disorganised chloroplasts and numerous starch grains were visible. In young galls, which are developed on young, emerging leaves, a large number of nuclei and enlarged vacuoles were visible, no typical leaf structure could be observed and there were much less tannin deposits. Idioblasts were not as clearly distinguishable as in mature galls and no cells with dense content were observed. The idioblasts in the (mature) leaves had a similar dense content as in the mature galls.

### Chlorophyll fluorescence kinetics

**Fig. 3:**
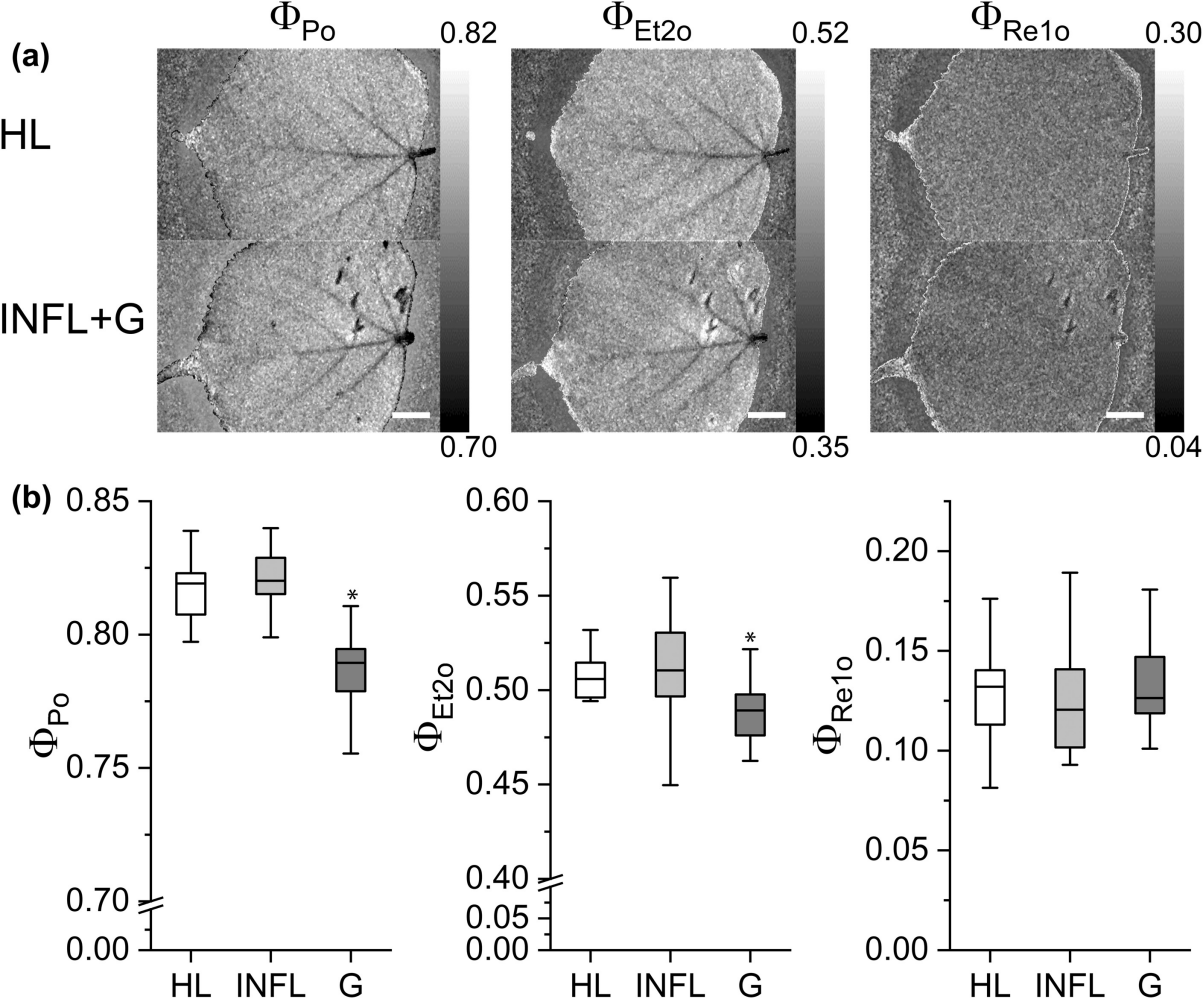
Heterogeneity of photosynthetic light reactions induced by mites on *Tilia cordata* leaves as measured by Chl fluorescence kinetics. (a) Macroscopic images for OJIP parameters (Φ_Po_, Φ_Et2o_ and Φ_Re1o_) in *T. cordata* leaves: HL, healthy leaf; INFL, infested leaf; G, nail gall; Φ_Po_, maximum quantum yield of primary PSII photochemistry; Φ_Et2o_, quantum yield of electron transport flux from Q_A_ to Q_B_; Φ_Re1o_, quantum yield of electron transport flux until PSI acceptors. Bars, 1 cm. (b) Differences in the OJIP kinetics between HL, INFL and G (*n* = 5). The line presents the median, the box shows the values between the 0.25–0.75 percentiles, and the bars show whiskers with 1.5 coefficient for outliers. Significant differences according to Mann–Whitney test (*, *P* < 0.05) between galls compared to INFL and HL are marked.

Direct imaging of fast Chl fluorescence kinetics (OJIP) was used to determine the photosynthetic capacity of the galls compared to leaf tissue. Strong inhibition of photosynthetic activity was observed in the nail galls, as measured by the maximum dark‐adapted PSII quantum yield (Φ_Po_) and the electron transport efficiency (Φ_et2o_), while there were no differences between the infested and healthy leaves. This shows the very localised effect of the mite infestation (Fig. 3). For electron transport towards PSI (Φ_re10_), a decreased activity was observed in individual leaves, but variability between the trees contributed to noisy data and no statistical significance (Fig. 3).

### Tissue and cellular distribution of elements in leaves and mite galls

**Fig. 4:**
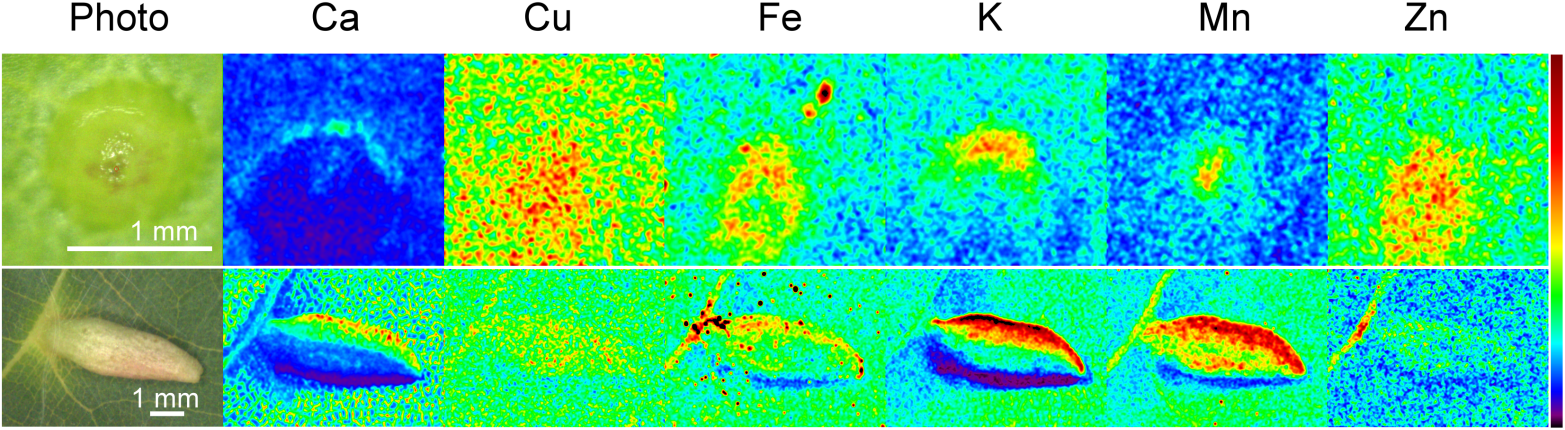
Benchtop μXRF maps showing element distribution (Ca, Cu, Fe, K, Mn, Zn) in young and mature nail galls on *Tilia cordata* leaves. The colour scale is from low (black) to the maximum observed for each element (dark red). It should be noted that some artefacts from the geometry of the gall exist and are caused also by the angle of the beam (coming in at 45°) during the measurements. These effects cause, for example, the stronger signals of elements at opposite sides of the galls, most visible for K as an element with low energy K‐edge. However, the elements have a different distribution (e.g. Zn compared to Mn and Fe) in particular in young galls despite identical geometry and similar energy range, which already indicated that the differential signals of elements in the galls were not due to geometry or thickness effects. To be able to reveal the distribution and concentration in galls and leaves fully quantitatively, μXRF tomography with absorption correction was performed (shown in Fig. 5).

Benchtop μXRF imaging was used to analyse the tissue distribution of several elements in the infested leaves with two developmental stages of galls, young and mature (developed). In both developmental stages, regardless of possible artefacts due to the geometry of the galls, strong Mn accumulation was observed compared to the surrounding leaf tissue and compared to other trace elements like Cu (excluding that this was only due to different tissue thickness). Further, in young galls, Ca, Cu, Fe, K and Zn accumulated to a higher extent than in the surrounding leaf tissue (Figs 4, S5). This indicated that metal metabolism already changes in the early phase of gall development before the tissue is fully differentiated. In mature galls, Ca, Mn and K accumulated to a higher level than in the surrounding leaf tissue, while the differences in Cu, Fe and Zn accumulation were not as pronounced as in young galls, maybe due to the dilution effect.

**Fig. 5:**
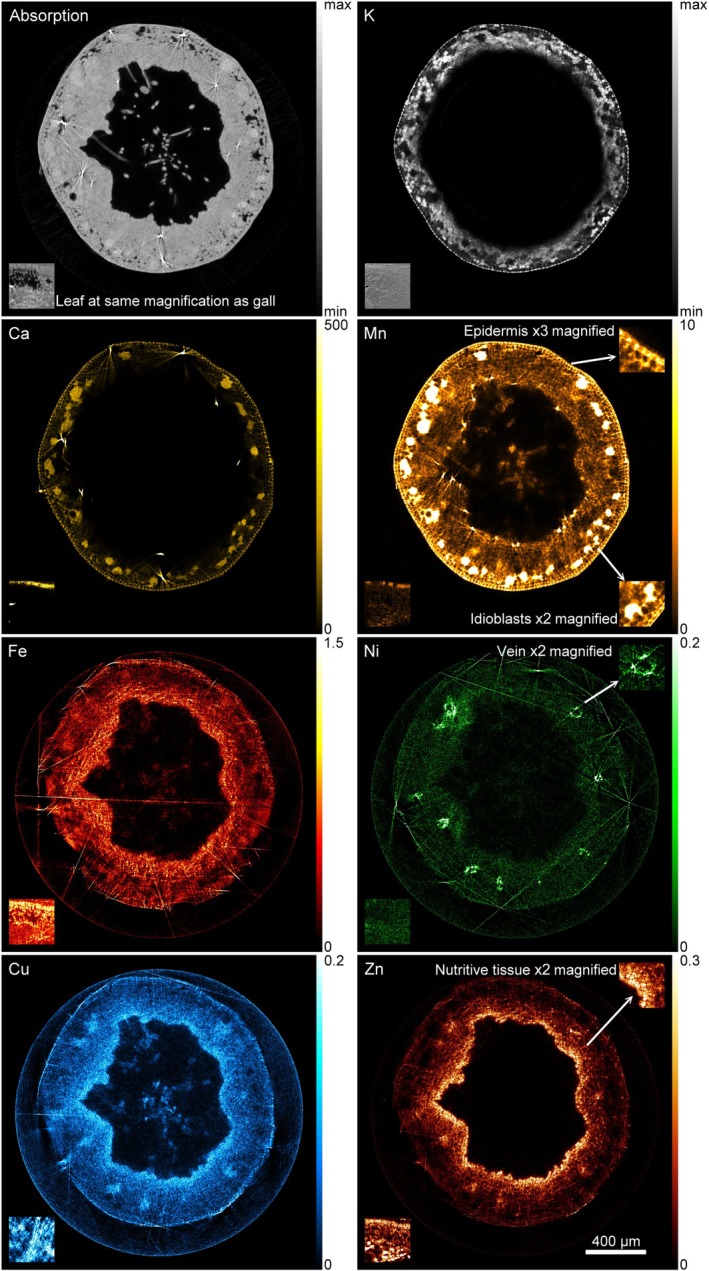
Synchrotron μXRF tomography of a shock‐frozen hydrated gall on a *Tilia cordata* leaf showing absorption contrast compared to the distribution of K, Ca, Mn, Fe, Ni, Cu and Zn. Bars, for K, 0 to a relative upper end (not quantitative due to too strong absorption), and fully quantitative including absorption correction for Ca (0–500 mM), Mn (0–10 mM), Fe (0–1.5 mM), Ni (0–0.2 mM), Cu (0–0.2 mM) and Zn (0–0.3 mM). Insets with a pointing arrow show enlarged details of gall tissues, in each case shown for the element where they are most visible. Insets in the right/left corner show the tomograms of the leaf for the same elements and the same scale bars. Typical images, each representative for the three replicate galls from different trees are shown. The other replicate galls and leaves measured are shown in Supporting Information Figs S6‐S8.

To further reveal element accumulation at the cellular level, shock‐frozen mature galls and leaves were analysed with synchrotron μXRF tomography (Figs 5, S6, S7). Potassium was visible in the epidermal cells and parenchyma cells further from the larval chamber, but not in idioblasts. Ca and Mn were strongly co‐localised in the idioblasts and both were accumulating in the epidermis to a lower extent. While in the epidermis and idioblasts Mn was clearly intracellular, in all other tissues of the gall Mn accumulated mostly in the cell walls. Intracellular Mn hot spots were observed in the nutritive tissue lining the chamber. Iron was distributed throughout the gall tissue; however, its accumulation was the highest in the nutritive tissue. The magnified view, showing a more structured filling of the cells than typical vacuolar sequestration, suggests cytoplasmic localisation. Nickel hot spots were found in the veins that were evenly distributed in the parenchyma. Cu and Zn hot spots were co‐localised inside the veins and in the nutritive tissue lining the chamber, where the accumulation of both Zn and Cu was the highest. None of the elements except Ca and Mn were increased in the idioblasts. The trichomes inside the larval chamber accumulated Mn, Fe and Cu. In one of the gall tomograms, mites were detected and visualised in the Zn map (Fig. S6).

The μXRF tomograms of leaves (Fig. S8) showed Ca and Mn accumulation mostly in subepidermal idioblasts on the abaxial and adaxial sides, similarly to the galls, but the cells were smaller as already seen in the LM and TEM. Further, Mn was found in photosynthetically active tissue, mostly in the palisade mesophyll and to a lower extent in the spongy mesophyll, as well as in the vascular tissues. Fe, Cu and Zn had a more homogeneous distribution mostly in the palisade and spongy mesophyll and vascular tissue. Zinc hot spots were also visible in epidermal cells. Copper maps were noisier due to low concentrations and absorption by the capillary but showed higher accumulation in the palisade mesophyll than in the remainder of the leaf. Nickel maps were too noisy to see the distribution at the cellular level, due to low concentration in the leaves regardless of the tissue type.

### Micro‐XANES tomography, Mn ligands

**Fig. 6:**
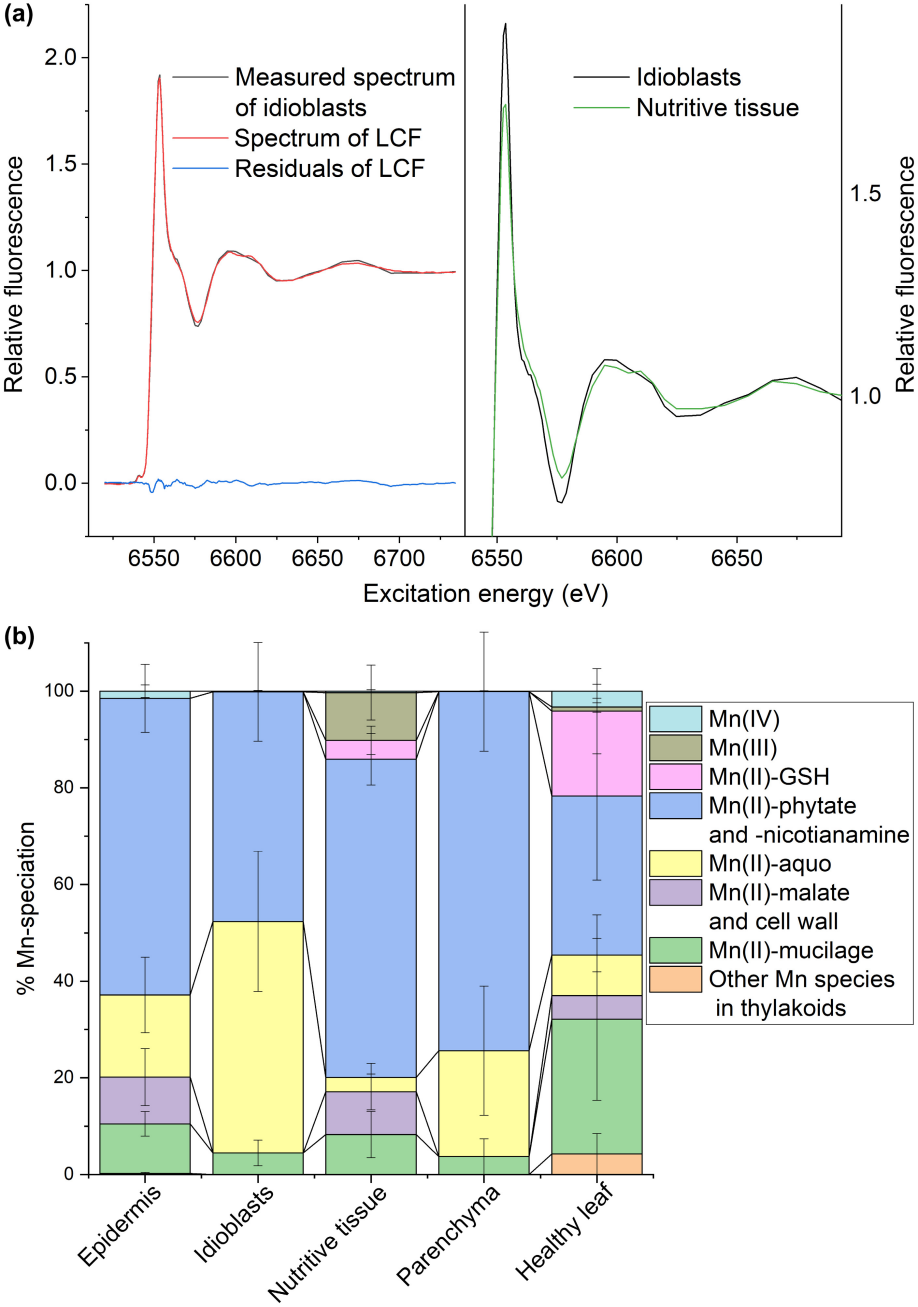
Analysis of Mn speciation by X‐ray absorption near edge structure (XANES) on selected tissues of galls and entire *Tilia cordata* leaves. (a) Left: data quality of micro‐XANES tomography spectra and quality of the linear combination fitting (LCF) of a set of model compounds to the spectra of idioblasts in galls. Right: comparison of the μXANES spectra of idioblasts and nutritive tissue. (b) Tissue‐dependent differences in ligand environment. Results of the XANES LCF showing the percentage contribution of different Mn species in gall tissues and the healthy leaf (average ± SE, *n* = 3). Gall and leaf XANES spectra were fitted with the following model compounds: aqueous (MnCl_2_), organic acids (Mn(II)‐malate), Mn(II) bound to isolated cell walls, Mn(II)‐nicotianamine and Mn(II)‐phytate, Mn(III) (oxide as model), Mn(IV) (oxide as model), Mn(II) bound to isolated mucilage, thiols (Mn(II)‐GSH), OEC (measured as native thylakoids).

Manganese accumulation in both young and mature galls seen by benchtop microXRF and its overall abundance in the bulk tissue compared to other micronutrients prompted us to further investigate its function. The synchrotron‐based μXRF tomography showed clear differences in cellular Mn distribution in leaves and galls. To identify the Mn speciation, XANES was applied in tomographic imaging and bulk mode (Fig. 6). The XANES spectra of the samples were fitted with a linear combination of several model complexes, with different Mn oxidation states. Micro‐XANES tomography was done on the same gall samples as the micro‐XRF measurements so that Mn distribution and speciation could be compared. The model complexes were chosen as likely Mn ligands and as representatives of O and S (glutathione, GSH) ligands. An example of a fit of the idioblasts spectrum is shown in Fig. 6(a), and for the other tissues in Fig. S9. The high quality of the XANES data and very small deviation between fits and measurements allowed for distinguishing even between spectroscopically relatively similar models, such as aquo, mucilage and organic acids (the latter represented by malate). However, since the spectra of Mn(II)‐malate and Mn(II)‐cell walls were almost identical, these species were fitted as a single contribution for the quantification. The same applied to Mn(II) phytate and Mn(II) nicotianamine.

In the plant samples, the shape of the XANES spectra showed that Mn complexation differs between the nutritive tissue compared to Mn‐loaded idioblasts (Fig. 6a, Table S2). The differences were generally most visible in the white line and the first minimum afterwards, but also in the next oscillation. Differences between other gall tissues were smaller. The semi‐quantitative analysis showed overall a high contribution of Mn(II)‐phytate/Mn(II)‐nicotianamine (47–74%) in all samples and tissues, but to a different extent. Idioblasts contained the highest proportion of Mn(II)aquo (*c*. 48%), equal percentage Mn(II)‐phytate/NA, and a much smaller contribution of Mn(II)‐mucilage (*c*. 5%). In the gall epidermis and parenchyma, Mn(II)aquo was lower than in the idioblasts (*c*. 20%), while Mn(II)‐phytate/NA was higher (61% and 74%, respectively). Additionally, the epidermis contained Mn(II)‐malate/CW and Mn(II)‐mucilage to a higher extent than parenchyma. The nutritive tissue of galls and the healthy leaf had the lowest contribution of Mn(II)aquo. Instead, these tissues contained more Mn(II)‐mucilage (*c*. 8 and 28% for the nutritive tissue and the leaf, respectively), as well as Mn(II)‐thiol bound (GSH). It should be noted that the contribution of thiol ligands may be underestimated due to their sensitivity to radiation. This became a more severe problem with higher redox states, which became obvious when comparing data from the μXANES measurement of models with bulk XANES measurements. The latter, originally meant for a full EXAFS analysis, became completely photoreduced to Mn(II) during those long measurements (data not shown). For this reason, EXAFS analysis became impossible, even for the bulk samples only XANES could be measured, and still, it is likely (cannot be finally determined) that a partial photoreduction decreased the percentage of higher oxidation states in our data. Nevertheless, Mn(III) (simulated with Mn(III) acetate) was reproducibly detected in the nutritive tissue (*c*. 10%), and small proportions of Mn(IV) (simulated by oxide as a model) were found in the epidermis. As expected, leaves contained the highest portion of Mn that could be best fitted with the mixed‐valence cluster of the OEC in thylakoids (*c*. 4%), and like in the gall epidermis a small proportion of Mn(IV) was detected. Further, leaves contained a much higher proportion of thiol‐bound Mn(II) than all tissues of the galls (Fig. 6b).

### EPR analyses

#### EPR detection of Mn^2+^

**Fig. 7:**
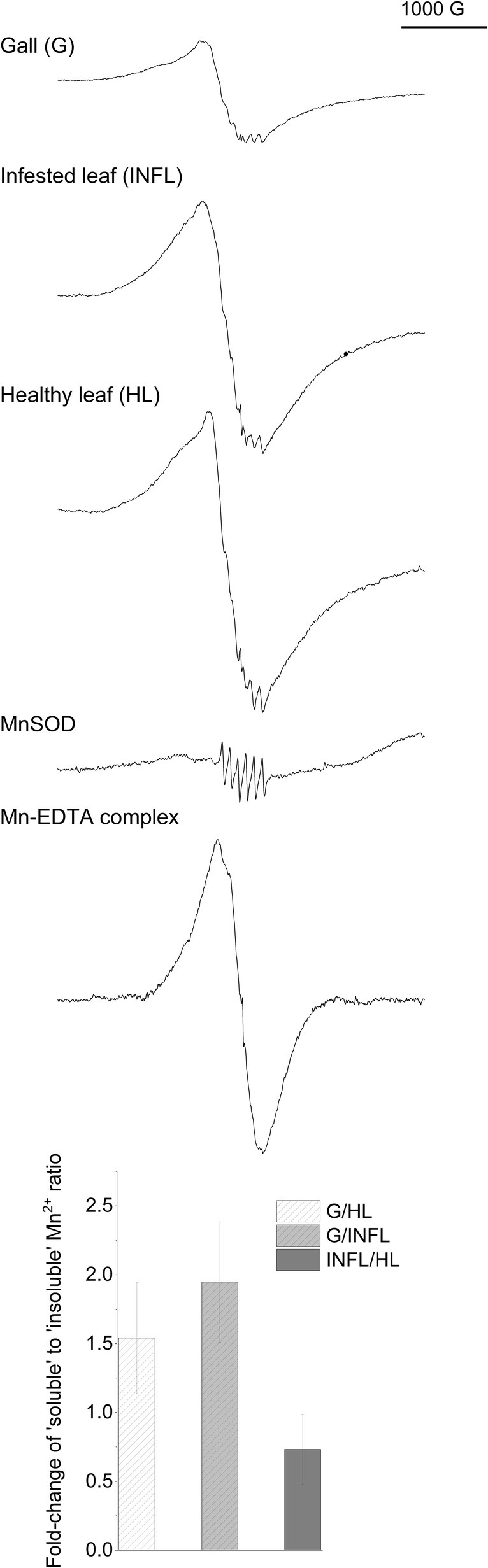
Analyses of Mn(II) speciation in mite nail galls on *Tilia cordata* leaves by electron paramagnetic resonance (EPR) spectroscopy. EPR spectra of Mn^2+^ from the intact mite galls, infested (INFL) and healthy leaves (HL), MnSOD (1 mg ml^−1^ in 0.6 mM HEPES buffer pH 7), and Mn‐EDTA complex (1 : 10, after drying). The characteristic six hyperfine lines of ‘soluble’ Mn^2+^ are visible, as well as the broad signal from ‘insoluble’ Mn^2+^ bound to cell wall macromolecules. The fold‐change in the ratio of ‘soluble’ to ‘insoluble’ Mn^2+^ is shown between the three tissue types ± SE (*n* = 3). Representative EPR spectra are shown.

The EPR signal of aqueous Mn^2+^ consists of a six hyperfine line spectrum due to freely rotating Mn^2+^ symmetrically coordinated by six molecules of water. This type of spectrum was detected not only for the aquo complex of ‘free’ Mn^2+^ but also for all soluble forms of strongly bound Mn^2+^ complexes, including Mn‐superoxide dismutase. Therefore, in the following discussion, we refer to this type of spectrum as ‘soluble’ Mn^2+^. The ‘soluble’ Mn^2+^ signal is superimposed with a broad Mn^2+^ signal, which was detected not only in tissues but also after drying an EDTA standard that initially displayed the ‘soluble’ spectrum. Therefore, we refer to it as ‘insoluble’ Mn^2+^, as it most likely originates from Mn complexes that are immobile or restricted in rotation, such as those associated with cell wall components and membrane proteins. This way it is possible to obtain information about Mn ‘availability’ in the galls and correlate it with its distribution. Fig. 7 shows the representative EPR spectra of intact galls and leaves measured at room temperature, the spectra of ‘soluble’ Mn incorporated in MnSOD and the spectra of ‘insoluble’ Mn in dry Mn^2+^‐EDTA complex. In galls, the ratio of ‘soluble’ to ‘insoluble’ Mn^2+^ was the highest, while the contribution of ‘insoluble’ Mn^2+^ was the highest in the healthy leaf.

#### EPR measurements of free radical generation in cell wall isolates

**Fig. 8:**
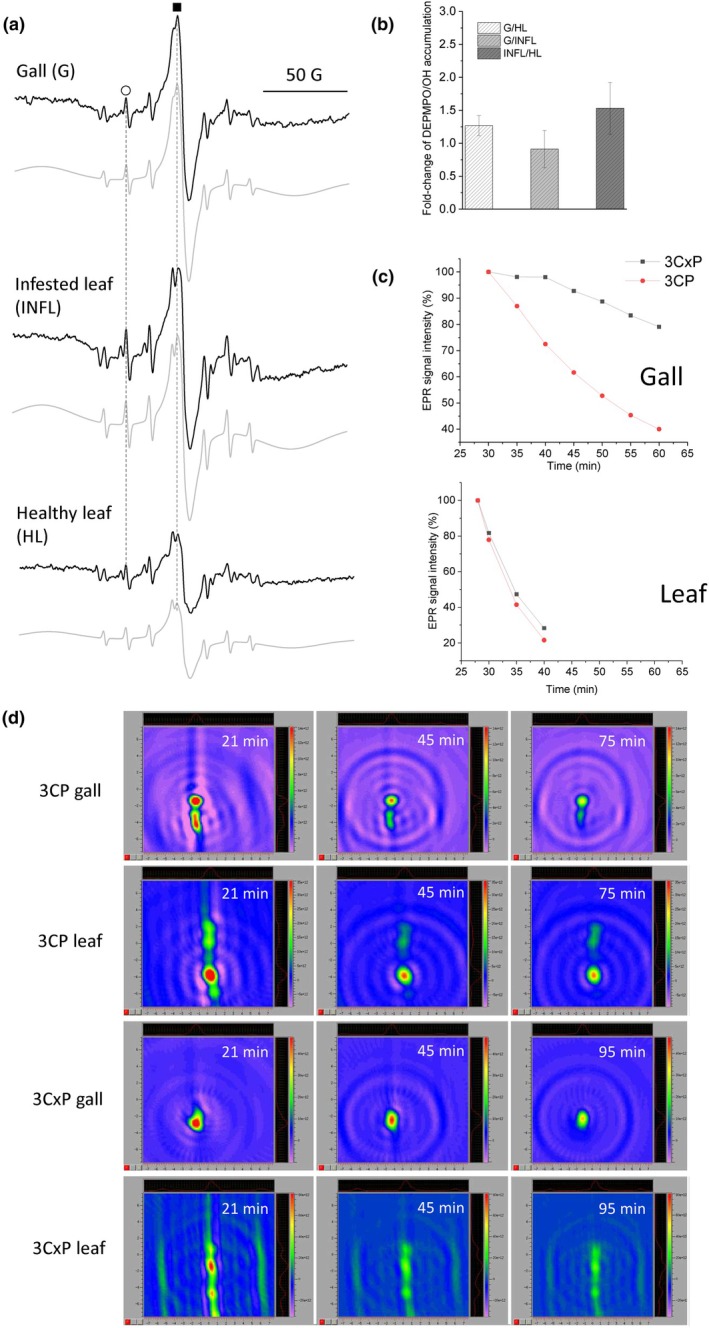
Analyses of reactive oxygen species (ROS) metabolism in galls vs leaves by electron paramagnetic resonance (EPR). (a) EPR spectra of DEPMPO/OH adducts (circle) and central quinhydrone peak (square) in isolated cell walls of mite galls (G), infested (INFL) and healthy (HL) leaves of *Tilia cordata* normalised to dry weight. (b) Fold‐change in DEPMPO/OH accumulation in the cell wall (CW) of different tissues ± SE (*n* = 5). (c) Kinetics of the EPR signal intensity reduction of spin probes 3CP (red circles) and 3CxP (black squares) in the intact galls and leaves obtained using 1D X‐band gradient imaging. (d) 2D X‐band EPR images of intact gall and infested leaf sections recorded 20, 45 and 75/95 min after administration of the spin probes 3CP and 3CxP. The colour scale shows the signal amplitude in arbitrary units, which represent the intensities/concentrations of the 3CP or 3CxP spin probe (red, high; dark blue and violet, low).

Gall growth and development can lead to changes in ROS accumulation and redox state in the tissues. To investigate ROS generation in the cell walls of galls and leaves, we used spin trap DEPMPO which makes long‐lived adducts with ·OH (Morina *et al*., 2010, Fig. 8). The computer simulation of EPR spectra showed that, besides the presence of the DEPMPO/OH spin‐adduct (a^P^ = 46.66 G, a^N^ = 13.78 G, a^H^[1H] = 13.32 G), the composite spectrum also includes a stable carbon‐centred radical (derived from quinhydrone structure) with *g* = 2.0042 and a signal corresponding to Mn^2+^, which is only partially visible within the observed 200 G scan range. In several spectra, a low signal corresponding to the DEPMPO carbon‐centred adduct (DEPMPO/CH_3_) was also detected, a phenomenon commonly observed in similar plant systems. The accumulation of the DEPMPO/OH adduct was the highest in the infested leaf, while it was similar between galls and the healthy leaf (Fig. 8a,b).

#### X‐band 1D gradient and 2D EPR imaging of extra/intracellular reduction of the pyrrolidine spin probes in intact galls and leaves

Pyrrolidine spin probes such as neutral membrane‐permeable 3CP and negatively charged membrane‐impermeable 3CxP can be used to investigate the variations in ROS concentration *in vivo* with spatiotemporal resolution. The EPR signal of 3CP and 3CxP decreases due to the one‐electron reduction of the probes to hydroxylamine. In all samples, depletion of 3CP and 3CxP demonstrated the presence of extra‐ and intracellular antioxidants. The kinetics of 3CP and 3CxP in the galls and infested leaves showed faster spin probe clearance in leaves than in galls, especially regarding the 3CxP decay rate, indicating a higher concentration of antioxidants in the apoplast (Fig. 8c). Moreover, the decay of extracellular 3CxP was slower than that of intracellular 3CP in galls.

Two‐dimensional temporal imaging of the capacity of galls and infested leaves to reduce the pyrrolidine spin probes was done after incubating the intact galls and leaf sections with the same concentrations of the probes (Fig. 8d). At the first measurable time point, the highest concentration of the probes was in the veins (leaf) and larval chamber (gall) – ‘hollow’ structures. 2D imaging showed that the EPR signal of the 3CP in the leaves decreased from 21 to 45 min and remained at a similar level in the mesophyll until the end of the measurement (75 min), while it continued decreasing in the veins. The EPR signal of 3CxP in the leaves, on the other hand, showed faster clearance in the veins than in the mesophyll between 21 and 45 min and slightly decreased at the end of the measurement (95 min).

In galls, these reactions were slower and the 3CP and 3CxP EPR signals continued decreasing both in the nail gall tip (largely occupied by parenchyma) and in the lower part with the larval chamber until the last time point measured. It followed similar kinetics as obtained with the 1D gradient (Fig. 8c), indicating a generally lower amount of antioxidants compared to the leaf tissue.

### Transcriptome *de novo* sequencing

*De novo* mRNA sequencing of *T. cordata* healthy and infested leaves and nail galls was used to determine the extent to which mites manipulate metal‐related GE and to identify metal‐related pathways in plant–mite interaction.

All three types of tissue (G, HL, INFL) had a similar number of clean reads (50 226 460 average), and *c*. 72% of reads were mapped. The *de novo* assembly of *T. cordata* transcriptome resulted in 140 119 unigenes, out of which 56.6% were annotated based on the nr NCBI database with the e‐value threshold set at < 10–5. About 78% of all unigenes were annotated in at least one database (nr, nt, SwissProt, PFAM, GO, KO and KOG), and the annotated genes are shown in Table S3.

The differences between galls compared to both leaf types were supported by the number of significantly differentially expressed genes (DEGs). About 24% of all the genes in the transcriptome were differentially expressed in galls compared to both healthy and infested leaves. Out of this, 78% (*c*. 28 000 genes) were upregulated in galls. The differences between the HL and INFL were much lower; only *c*. 0.1% DEGs of the total gene number, out of which 18% genes (*c*. 500) were upregulated in the INFL, indicating that the effect of gall formation was very localised and did not affect the surrounding tissue to a great extent (Fig. S10).

### Metal‐related DEGs in response to mite infestations

Here we have focused on the genes that are involved in metal metabolism, namely Cu, Fe, Mn, Ni and Zn to explain the role of these micronutrients in plant–galler interactions. All genes with any evidence of expression (FPKM > 0 in any of the tissue types) were considered, with a significant adjusted *P*‐value (*P*_adj_) < 0.05. In the case of several genes, FPKM values > 0 were obtained only in galls (induced by mites).

**Fig. 9:**
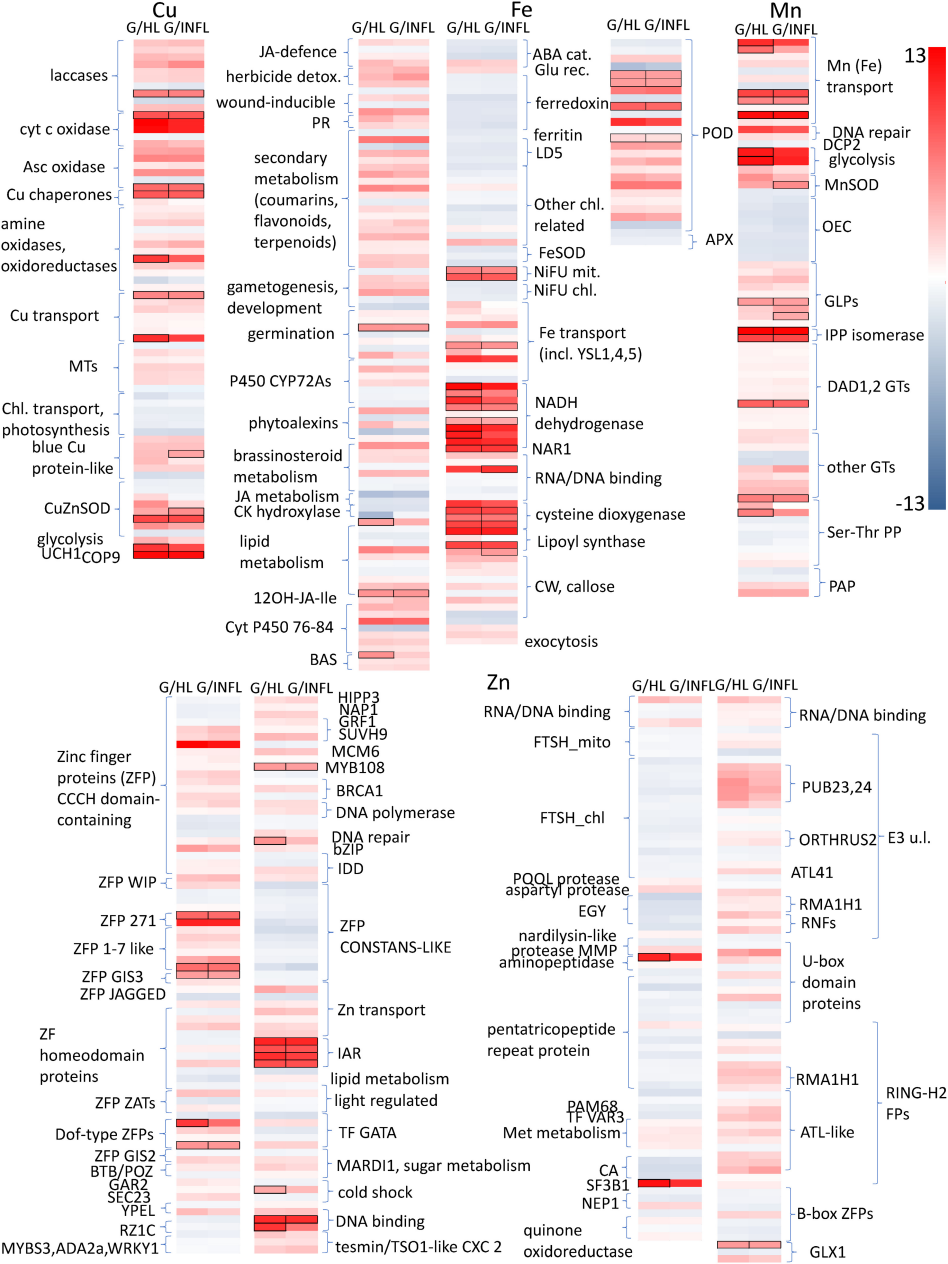
Transcriptomic changes in metal‐related genes induced by gall formation on *Tilia cordata* leaves. Heatmaps of DEGs with adjusted *P*‐value (*P*_adj_) < 0.05 related to Cu, Fe, Mn and Zn‐binding proteins, transporters and transcription factors based on log_2_ fold value between nail galls (G) compared to healthy (HL) and infested leaves (INFL) and between INFL and HL *T. cordata* leaves after *de novo* mRNA sequencing (*n* = 4). Genes with expression marked with borders were inducible only in galls (FPKM was > 0 only in galls). MTs, metallothioneins; JA, jasmonic acid; PR, pathogen response; CK hydroxylase, cytokinin hydroxylase; BAS, brassinosteroid oxidase; ABA, abscisic acid; LD5, protein LUTEIN DEFICIENT 5; NiFU, NifU‐like domain‐containing proteins; NAR1 iron, sulphur cluster protein; CW, cell wall; PODs, peroxidases; APX, ascorbate peroxidase; DCP2, mRNA‐decapping complex; OEC, oxygen evolving complex; GLP, germin‐like proteins; IPP isomerase, isopentenyl diphosphate delta isomerase; DAD1,2, dolichyl‐diphosphooligosaccharide‐protein glycosyltransferase subunits 1 and 2; other GT, glycosyltransferases; Ser‐Thr PP, serine/threonine‐protein phosphatases; PAP, purple acid phosphatases; IAR‐IAA, alanine resistance protein 1; FTSH, ATP‐dependent zinc metalloprotease FTSH; E3 u. l., E3 ubiquitin ligase; CA, carbonic anhydrase; GLX1, glyoxalase I.

Common for all four metals was the downregulation of genes related to chloroplasts and photosynthesis regulation, OEC, chloroplastic FTSH and NiFU, ferredoxin, ferritin and chloroplastic Cu transport (Fig. 9).

The main classes of Cu‐related DEGs were laccases, ascorbate oxidase, other blue copper proteins, cytochrome *c* oxidase and amino oxidases (mostly upregulated). The expression of genes regulating Cu transport (COPT1 and COPT5), metallothioneins (MTs type 1, 2, 3) and two Cu chaperons were strongly upregulated. The main Fe‐binding proteins differentially regulated in the galls compared to the leaves were from the diverse family of cytochromes P450, which have been grouped according to their functions where known (hormone signalling, detoxification, metabolite biosynthesis). P450 genes involved in herbicide detoxification (CYT P450 714 family), wounding (P450 81D11), pathogen response and secondary metabolism (CYT P450 71 family, P450 706, P450 736) and lipid metabolism (CYT P450 86A22) were mainly upregulated. P450 genes involved in jasmonic acid metabolism (P450 94C1), as well as abscisic acid (ABA) catabolism (abscisic acid 8′‐hydroxylase 4) and cytokinin hydrolase were mainly downregulated.

Gene expression of ferredoxin, and all chloroplast‐related Fe‐binding proteins was downregulated, as well as the iron storage protein ferritin and *FeSOD*. Pronounced changes in GE were observed in mitochondria, that is, upregulation of NifU‐like domain‐containing proteins and NADH dehydrogenases (mostly detected after infection; contain FeS‐active site). Transporters including *YSL1* and *YSL5*, and nicotianamine synthase were upregulated in the galls. Overall, GE of peroxidases (*POD*s) was mostly upregulated in the galls, while ascorbate peroxidase activity was slightly downregulated.

Among the DEGs related to Mn metabolism, several Mn transporters (including VIT family transporters) and germin‐like proteins (*GLPs*) and *MnSOD*, were upregulated. Further, genes involved in DNA repair were upregulated, as well as phosphatase 2C, which is involved in the regulation of growth, development and stress acclimation. Gene expression of isopentenyl diphosphatase (IPP) was inducible by mites and upregulated in the galls, as well as genes regulating N‐glycosylation of proteins, dolichyl ‐diphosphooligosaccharide‐protein glycosyltransferases (*DAD*s) and other glycosyltransferases (*GT*s) such as xyloglucan *GT*s. Besides the transporters mentioned above that can use both Ca and Mn as a substrate, as well as Fe (e.g. VIT), vacuolar Ca transporters *CAX3* and *CAX5* were mostly downregulated. Plasma membrane Ca ATPases were slightly upregulated (one was highly inducible by the mites, *ATPase 12*), as well as endoplasmic reticulum‐type‐like Ca ATPases (Table S4).

Changes in the expression of Zn‐related genes were the most abundant, in accordance with the high abundance of Zn‐proteins in plants. Zinc finger proteins (ZFPs) with the CCCH domain were mostly upregulated, as well as ZFP *WIP*, ZFP 271, 1‐7 like, *GIS3*, dof‐type ZFPs, *GAR2* and *SEC23*, and tesmin/*TSO1 CXC2*. All *FTSH* and *EGY* metalloproteases in both mitochondria and chloroplasts were downregulated, as well as pentatricopeptide repeat proteins. Methionine, lipid and sugar metabolism‐related Zn‐enzymes and quinone oxidoreductase were upregulated. From the class of Zn binding E3 ubiquitin ligases, *PUB23*, *PUB24* and *ORTHRUS2*, *RMA1H1*, *RNF*s and *ATL41* were upregulated, as well as several U‐box domain proteins, and RING‐H2 FPs, while B‐box ZFPs were mainly downregulated. Genes involved in Zn transport, *ZNT1*, *ZNT5*, *ZNT4* and *ZNT6*, were upregulated in galls. Two glyoxylate isoforms were upregulated in galls.

Gene expression of inositol polyphosphate 5‐phosphatase (Mg as cofactor), involved in the biosynthesis of phytic acid, was both up‐ and downregulated in galls, and two isoforms were inducible by mites. Gene expression of urease (Ni as active centre) was slightly but statistically significantly higher in galls.

### Metabolomics

**Fig. 10:**
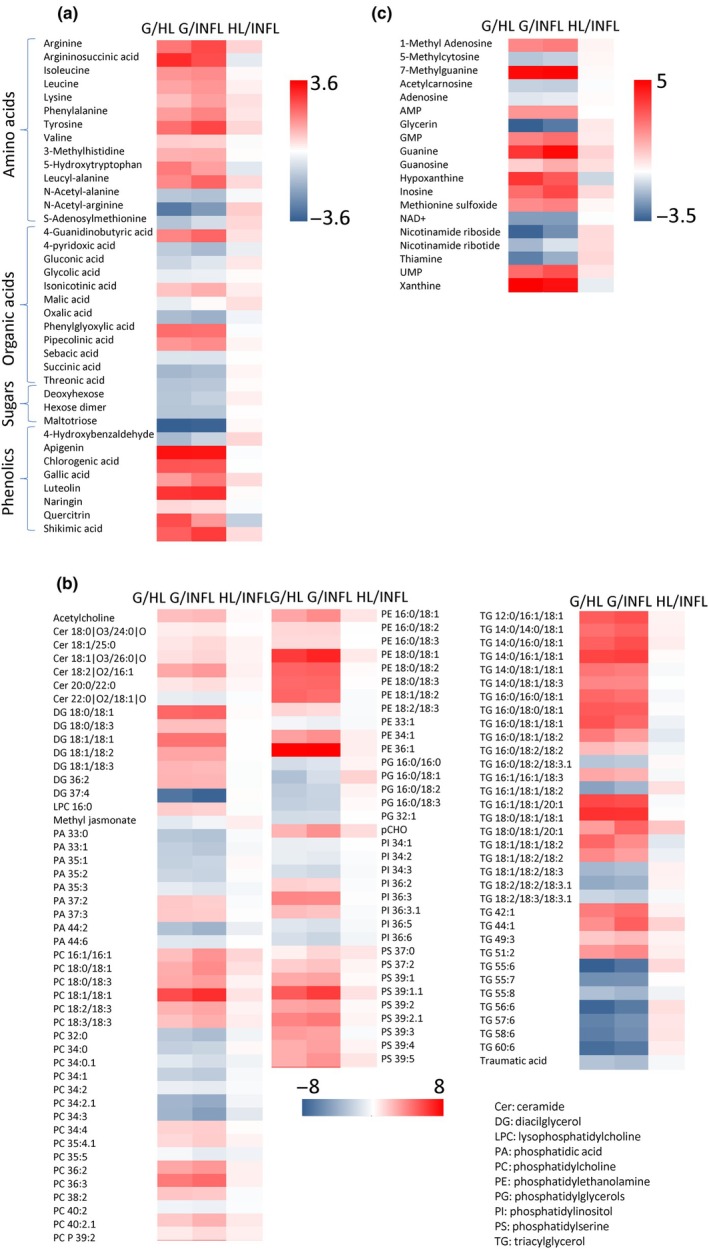
Effects of gall formation on the *Tilia cordata* leaf metabolome. Log_2_ fold‐change in significantly differentially accumulated metabolites in galls (G) compared to healthy (HL) and infested leaves (INFL) and between HL and INFL (*n* = 4). (a) Amino acids, organic acids, sugars and phenolics; (b) lipidomics; (c) other compounds.

Metabolomic analyses revealed an increased accumulation of several amino acids in galls compared to both leaf tissues except acetylated ones and S‐adenosylmethionine (Fig. 10). Among organic acids, the most upregulated ones were 4‐guanidinobutyric acid, phenylglyoxylic acid, pipecolinic acid and isonicotinic acid. Phenolics (mainly apigenin, luteolin, chlorogenic acid, gallic acid, quercitrin and shikimic acid) were also upregulated in galls compared to both leaf types.

Lipidomic analyses revealed that compounds from two phospholipid classes, both membrane constituents, accumulated in galls compared to HL and INFL (phosphatidylserines (PSs) and phosphatidylethanolamines (PEs)). In line with inhibited photosynthesis and disturbed chloroplast structure, the level of thylakoid membrane lipids, phosphatidylglycerols (PGs) decreased in galls. By contrast, ceramides (sphingolipid class) were upregulated; they support membrane structure and mediate cell signalling events. Diacylglycerols (DGs), short‐chain triacylglycerols (TGs), and TGs with 42–51 carbon atoms generally accumulated in galls, while the amount of TGs with 55–60 total carbon atoms decreased.

The content of phosphatidic acid, precursor of DGs, TGs and PCs generally decreased in galls compared to both leaf types. Furthermore, the levels of methyl jasmonate and traumatic acid slightly decreased in galls compared to leaves.

The contribution of mite metabolism (especially in the case of lipids) was considered low because of a very small weight, but histochemical staining confirmed increased lipid accumulation in galls, and leaves (described in Guedes *et al*., 2023).

## Discussion

Evolutionary advantages of gall formation include nutrition, protection and microenvironment for galling mites. Among the nutrients, trace metals are still underexplored when gall development is in question. Here, we reveal the increased accumulation and distinct distribution of several essential metals, Zn, Cu, Fe, Mn, Ni and Ca at the cellular level, in neoformed nail galls induced by eriophyid mites on the leaves of *T. cordata*. We explain their function based on our data of GE of metalloproteins, speciation of Mn and profile of metabolites including ROS. By contrast, previous work mostly dealt with the accumulation of sugars and amino acids in galls, or the total metal content (Koyama *et al*., 2004; Huang *et al*., 2015; Zorić *et al*., 2019; Arriola *et al*., 2024).

### Gall differentiation dramatically changes Mn distribution and speciation

Following gall initiation by active compounds in the saliva of mites, extensive transformation of leaf tissue takes place, through cell hypertrophy, hyperplasia and re‐differentiation (Petanović & Kielkiewicz, 2010a, 2010b; Guedes *et al*., 2023). The current study revealed that these morphological changes are directly linked to fundamental changes in micronutrient distribution and speciation. Metal mobilisation occurred in both young and developed galls pointing to their crucial role in the interactions with the host during tissue differentiation and in mature galls with an established nutritive chamber. The initiation of mite galls starts on emerging young leaves with different metabolites than the mature ones. The involvement of metals in these early stages of development is yet to be explored.

In comparison with a healthy leaf, radial growth of a nail gall occurs between the epidermis and subepidermal (hypodermal) layer of secretory cells, idioblasts, developing outer parenchyma cells. Idioblasts were found to be hot spots of Mn and Ca accumulation in both galls and leaves. Moreover, they were *c*. 10× enlarged in mature galls compared to leaves, and their Mn accumulation was more obvious than in leaves because of lowered Mn in the other cells (parenchyma in galls vs mesophyll in leaves). Although not a Mn hyperaccumulator, *T*. *cordata* contains a higher concentration of Mn than other microelements, which is why its homeostasis has to be tightly regulated. In the Mn hyperaccumulator *Maytenus fournieri*, Mn was also sequestered in large non‐photosynthetic hypodermal cells, while in other Mn‐hyperaccumulating species it was mostly accumulated in the palisade mesophyll (Fernando *et al*., 2012).

Increased Mn detoxification in gall idioblasts may be crucial for maintaining Mn homeostasis. To reveal this, we employed complementing spectroscopy techniques to study the speciation (including ligands) of Mn in nail galls and leaves. EPR analyses showed a higher contribution of ‘soluble’ Mn^2+^, bound to LMW ligands and soluble proteins than ‘insoluble’ Mn^2+^ (bound to nondissolved ligands such as cell walls) in intact galls than in leaves. The contribution of Mn^2+^ in the OEC in the thylakoids did not contribute to the EPR spectra under our experimental conditions. While only EPR could make this distinction between states of Mn^2+^ solvation, XANES analysis could reveal changes between redox states and chemically or structurally different ligand types. It clearly showed a higher contribution of Mn(II)‐aquo complexes in idioblasts than in the other gall cells or the leaf, followed by Mn^2+^ complexation with NA and phytate. This type of speciation indicates a homeostatic role of idioblasts. Mn transport and sequestration to the vacuoles, endoplasmic reticulum and Golgi is done by binding to LMW ligands, including organic acids, NA and phytate (Alejandro *et al*., 2020). Most of the Mn in plants is in Mn^2+^ oxidation state, with Mn^3+^ and Mn^4+^ only present in enzymes and OEC (Robblee *et al*., 2001; Zhu & Richards, 2017). We showed that idioblasts accumulate not only mucilage/pectin but also other compounds; presumably, LMW Mn ligands are secreted as well. Co‐localisation of Mn and Ca is not surprising considering that they share a number of transporters (He *et al*., 2021). The increased vacuolar transporter GE (*VIT*, *CAX*, *ZIP*) and large volume of these cells may be crucial for safe storage of high Mn concentrations that are potentially toxic for mites. Altogether, based on our results, Mn accumulation in idioblasts likely has a detoxification function.

### The role of trace metals in the nutritive tissue

While Mn accumulation in galls was the highest in idioblasts, some accumulation was also found in the nutritive tissue lining the gall chamber. Mn, as well as Cu, Fe, Ni and Zn, is essential for both the plant and the galler (Arriola *et al*., 2024). Here, XANES data show different Mn chemistry and therefore its different function in the nutritive tissue compared to idioblasts, with higher contribution in particular of Mn(III), and thiol ligands (simulated by GSH) that are typical for Mn binding in enzymes. GE of Mn‐dependent *GT*s was upregulated in galls pointing to enhanced post‐translational N‐glycosylation of proteins. Among them were xyloglucan *GT*s, which are involved in re‐modelling of the cell wall (Pauly & Keegstra, 2016), and seem to be essential for the actively dividing and growing cells. Furthermore, glycoproteins are important for cell recognition, differentiation and development, signal transduction and immune response (Nagashima *et al*., 2018). In this way, we conclude that in the nutritive tissue, Mn is actively involved in cell metabolism and interactions with mites.

Even more than Mn, Cu, Fe and most of all Zn were highly enriched in the nutritive tissue. Upregulation of genes regulating several metal transporters, and Cu chaperones, MTs, NA and phytic acid in galls shows active mobilisation of essential Cu, Fe, Zn and Ni to galls. The active uptake of these nutrients is not only to support the nutrition of the mites but also for the extensive metabolic processes discussed further. The majority of metal‐related DEGs were those regulating the expression of Zn‐proteins, including CCCH domain‐containing ZFPs, E3 ubiquitin ligases, RING H2 FPs and numerous other enzymes. Our findings point to the crucial role of Zn in gall formation and maintenance, especially in the nutritive tissue, metabolically intensively active, in which it was accumulated to the highest extent.

However, ROS regulation and the redox environment in nail galls were also regulated by metal accumulation, most likely by the strong accumulation of Cu and Fe in the nutritive tissue. ROS are essential messengers of redox and energy state from different cellular compartments to the nucleus (Noctor *et al*., 2018). ROS homeostasis in the apoplast is crucial for the regulation of cell wall relaxation (cell expansion) on one side, and cell wall stiffening on the other, and both processes are involved in gall initiation and development (Kärkönen & Kuchitsu, 2015). Increased GE of apoplastic enzymes that regulate ROS homeostasis in the galls included *POD*s, amine oxidases and *GLP*s (H_2_O_2_ generating), and ascorbate oxidase. EPR analyses using the spin trap DEPMPO showed lower ·OH generation capacity in the isolated cell walls of the gall, while the decay rate of membrane‐impermeable pyrrolidine spin probe 3CxP was lower than in the infested leaf. These results indicate that the reducing capacity of galls in the apoplast may be related to the depletion of reduced ascorbate and other antioxidants, and to elevated H_2_O_2_ levels.

Furthermore, our work strongly indicates another important role in particular of Cu and Fe accumulation in the nutritive tissue and to some extent in the outermost cell layers of the galls. This is the synthesis of phenolic compounds by Cu‐ and Fe‐dependent enzymes. Phenylalanine accumulates in nail galls and serves as a precursor to phenolic compounds like flavonoids, tannins and finally lignin (Barros & Dixon, 2020). This accumulation of secondary metabolites in our study, including phenolic acids and isoprenoids, is associated with the upregulation of cytochrome P450 enzymes, IPP isomerases, and phenoloxidases (*PPO*‐Cu, *POD*‐Fe) that utilise aromatic compounds as substrates. The upregulated GE of Cu‐dependent *POD*s, amine oxidases and laccases points to cell wall maturation and lignification of the gall to provide mechanical support. In line with this, histochemical staining revealed proanthocyanidin accumulation in outer parenchyma cells and cells around the nutritive tissue, matching the distribution of Fe, Mn and Cu. This gradient of proanthocyanin accumulation in galls has been linked to antioxidative functions and protection against abiotic stress (Carneiro & Isaias, 2015). Healthy *Tilia* leaves have less tannins (mostly in epidermal cells and vascular tissue) and lipids (cuticle, cell wall, epidermal cells) than a nail gall, as described by Guedes *et al*. (2023).

Increased H_2_O_2_ accumulation and lignification have been observed in other gall systems (Ferreira *et al*., 2018; Takeda *et al*., 2019). The slower rate of 3CP decay in the gall compared to the leaf observed here may point to lower total reducing capacity of the gall and/or increased levels of ROS than the leaf, although tissue‐specific differences in the reducing capacity can be expected, but were not considered in this study. Furthermore, more dramatic dynamics of ROS accumulation may be expected in the earlier phase of gall initiation and development. Furthermore, two negative regulators of plant immunity, *PUB23* and *PUB24* (Zn), were upregulated in galls, while the jasmonate pathway was supressed, indicating that, in contrast to the plant–pathogen response, the galler suppressed pattern‐triggered immunity (PTI) in the host. These two proteins downregulate the early oxidative burst and regulate ROS generation in later stages of the immune response (Trujillo *et al*., 2008).

### Metabolic shifts from photosynthesis to supply energy and nutrients to gall‐inducing mites

In galls, mesophyll cells, to a large extent, lose their photosynthetic function (Huang *et al*., 2015) and are re‐differentiated to inner parenchyma and nutritive tissue, while the inner epidermis is lining the gall chamber and has nonglandular trichomes. Likewise, in *T. cordata* nail galls, major metal‐related genes involved in photosynthesis were downregulated (OEC, ferritin, pentatricopeptide repeat proteins, chloroplastic Cu‐transporting ATPases). This was correlated with decreased capacity for photosynthesis visible in OJIP parameters (Φ_Po_, Φ_et2o_) and a low concentration of Fe and Mn in the parenchyma of galls compared to healthy leaf mesophyll. A shift towards glycolysis was observed in our study, through a decrease in the tricarboxylic acid cycle. Glycolysis is enhanced in low‐oxygen environments such as galls (Nabity *et al*., 2013; Kerpen *et al*., 2019), with compromised photosynthesis. Furthermore, depletion of NAD^+^ and its precursors nicotinamide riboside and nicotinamide ribotide indicates active glycolysis in high energy demand state galls so that NAD^+^ is consumed faster than it is regenerated.

The metabolomic profile of nail galls shows higher accumulation of free amino acids and specific lipids that aid gall development and the galler's growth. Free amino acids are more digestible than polypeptides and are essential for the galler's metabolic activity. Arginine, the main nitrogen storage in the galls, is converted to urea and ammonia by upregulated urease (Ni), supporting tissue growth (Winter *et al*., 2015). This could explain the specific accumulation of Ni in the bundle sheath of veins in galls.

Significant changes in cell growth and division in galls can affect membrane composition and fluidity, and a shift in lipid metabolism. The content of thylakoid PGs on nail galls decreased in line with changes in chloroplast ultrastructure and inhibition of photosynthesis. On the other hand, ceramides, DGs, PSs, PEs and the majority of TGs accumulated to a higher level than in leaves. Further, the GE of fatty acyl‐CoA hydroxylase (Fe), enzymes that modify fatty acyl‐CoA derivatives and thus play a significant role in lipid metabolism, was upregulated in galls. Glycerophospholipids play vital roles in cell membranes and cellular signalling. The shift in lipid composition in the galls towards energy storage (TGs) may be linked to enhanced glycolysis, while other functions of upregulated lipids such as membrane integrity, structure and growth, as well as stress response and signalling (Yang & Benning, 2018) indicate their important role in plant–galler interactions. Lipid detection in nail galls is in line with these findings, as lipids were detected in membranes and in cells lining the gall chamber.

The findings of our study provide new insight into plant–galler interactions in relation to metal distribution and function. The distribution of metals indicates that excessive Mn is sequestered in idioblasts, while Zn accumulates in the nutritive tissue to accommodate the needs of the gallers. In addition, the distribution of Cu and Fe, and increased accumulation of secondary metabolites, support the protection hypothesis. Based on our results, metals have a crucial role in metabolic processes in galls, beyond their nutritive function.

## Competing interests

None declared.

## Author contributions

FM was involved in conceptualisation, project administration, writing – original draft, writing – review & editing, supervision, methodology and investigation, visualisation, data curation and funding acquisition. AK was involved in visualisation, investigation, formal analysis and writing – review & editing. DB was involved in visualisation, writing – review & editing, methodology, investigation, data curation and formal analysis. MM contributed to methodology, investigation, formal analysis and writing – review & editing. DN contributed to investigation, formal analysis and writing – review & editing. SNHB did the writing – review & editing and formal analysis. BV contributed to methodology and writing – review & editing. GF took part in methodology, investigation and writing – review & editing. HK was involved in conceptualisation, methodology, visualisation, investigation, formal analysis, data curation, writing –review & editing, supervision, resources and funding acquisition.

## Disclaimer

The New Phytologist Foundation remains neutral with regard to jurisdictional claims in maps and in any institutional affiliations.

## Supporting information


**Fig. S1** The locations of the *Tilia cordata* leaves used in the study.
**Fig. S2** Total element content measured by ICP‐MS in the nail galls induced by mites.
**Fig. S3** Fold‐change in the element concentrations in the tissues.
**Fig. S4** Ultrastructure of *Tilia cordata* leaves, mature and young galls.
**Fig. S5** Benchtop micro‐XRF maps showing element distribution in mature and young nail galls.
**Fig. S6** Synchrotron micro‐XRF tomography of a shock‐frozen gall (replicate 2).
**Fig. S7** Synchrotron micro‐XRF tomography of a shock‐frozen gall (replicate 3).
**Fig. S8** Synchrotron micro‐XRF tomography of shock‐frozen leaves.
**Fig. S9** μXANES linear combination fits of all plant samples analysed for this work.
**Fig. S10** Volcano plot of DEGs in the infested leaves compared to healthy ones.

**Methods S1** Bulk element content in the plants and in the soil.
**Methods S2** Histochemical staining.
**Methods S3** Details of the measuring conditions for X‐ray spectroscopy.
**Methods S4** Quantifying micro‐XRF tomograms with absorption correction for nail galls as elliptical hollow objects.

**Notes S1** mRNA analyses.

**Notes S2** Untargeted and targeted metabolomics.

**Table S1** Concentrations of bioavailable elements in the topsoil.
**Table S2** Statistics of XANES LCF fits.
**Table S3** mRNA sequencing, all annotated genes.

**Table S4** mRNA DEG metal metabolism.
Please note: Wiley is not responsible for the content or functionality of any Supporting Information supplied by the authors. Any queries (other than missing material) should be directed to the *New Phytologist* Central Office.

## Data Availability

Data are available as Supporting Information. Raw read data of mRNA‐seq are deposited in the European Nucleotide Archive (ENA) under the project accession no. PRJEB82870.
