# Supplementary material for: How eriophyid mites shape metal metabolism in leaf galls on Tilia cordata
Source: New Phytol. 2025 Apr 16;246(5):2222–42. doi: 10.1111/nph.70103 (PMC12059528; doi:10.1111/nph.70103)
Supplement: Supplementary file 1 — Fig. S1 The locations of the Tilia cordata leaves used in the study. Fig. S2 Total element content measured by ICP‐MS in the nail galls induced by mites. Fig. S3 Fold‐change in the element concentrations in the tissues. Fig. S4 Ultrastructure of Tilia cordata leaves, mature and young galls. Fig. S5 Benchtop micro‐XRF maps showing element distribution in mature and young nail galls. Fig. S6 Synchrotron micro‐XRF tomography of a shock‐frozen gall (replicate 2). Fig. S7 Synchrotron micro‐XRF tomography of a shock‐frozen gall (replicate 3). Fig. S8 Synchrotron micro‐XRF tomography of shock‐frozen leaves. Fig. S9 μXANES linear combination fits of all plant samples analysed for this work. Fig. S10 Volcano plot of DEGs in the infested leaves compared to healthy ones. [file NPH-246-2222-s006.pdf]

***New Phytologist* Supporting Information**

Article title: How eriophyid mites shape metal metabolism in leaf galls on Tilia cordata

Authors: Filis Morina[1*], Anđela Kuvelja[1,2], Dennis Brückner[3], Miloš Mojović[4], Đura Nakarada[5], Syed Nadeem Hussain Bokhari[1], Bojan Vujić[1], Gerald Falkenberg[3], Hendrik Küpper[1,2*]

Article acceptance date: 06 March 2025

The following Supporting Information is available for this article:

Fig. S1 The locations of the *Tilia cordata* leaves used in the study in České Budějovice, Czechia. Aerial maps were obtained from mapquest.com (January 2025). Red dots show individual trees used for sample collection (n=18).

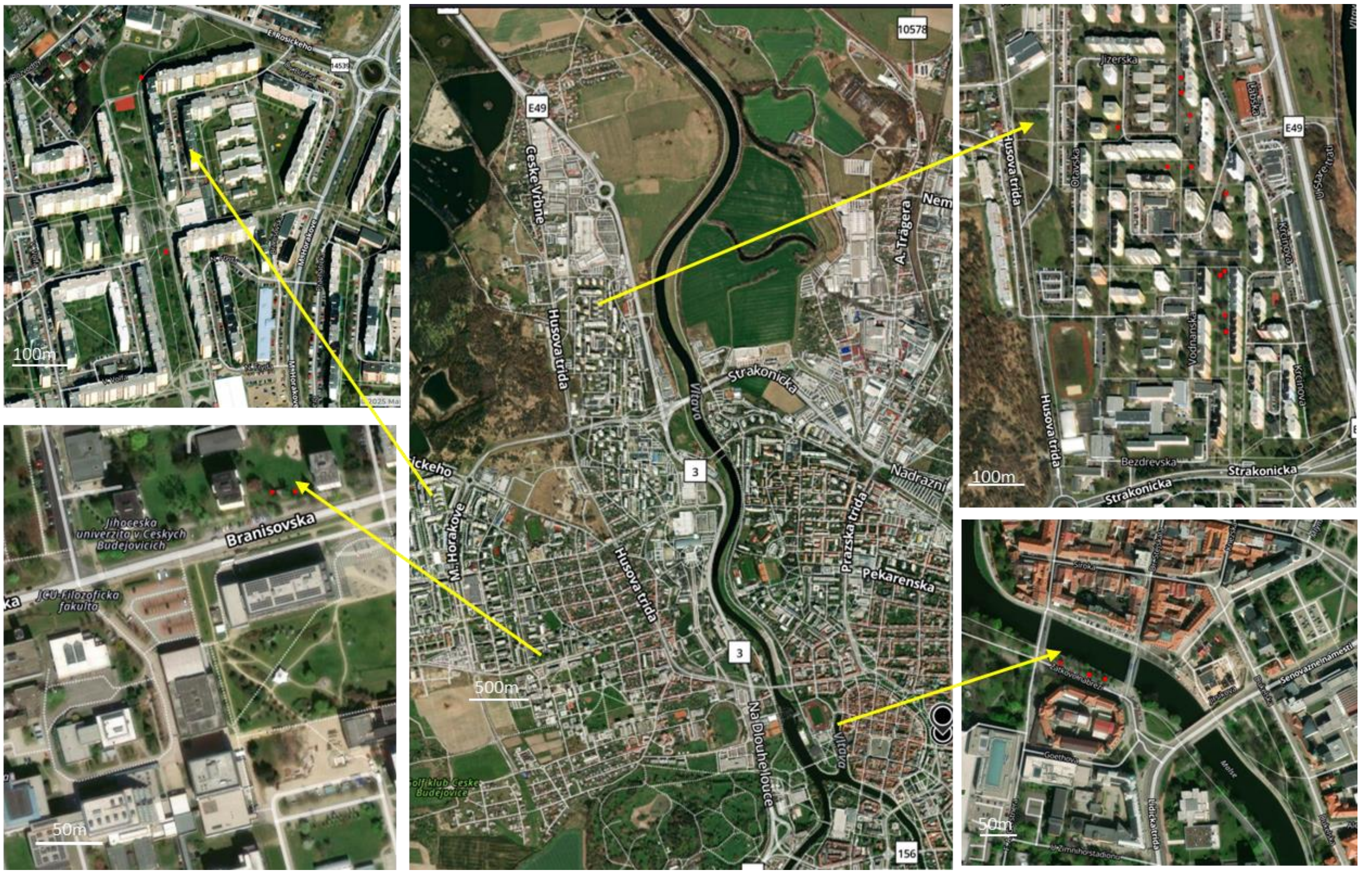

Fig. S2 Total element content measured by ICP-MS in the nail galls induced by mites on *Tilia cordata* (G), healthy leaves (HL) and infested leaves (INFL). The samples were collected from 18 trees. Each dot represents one sample.

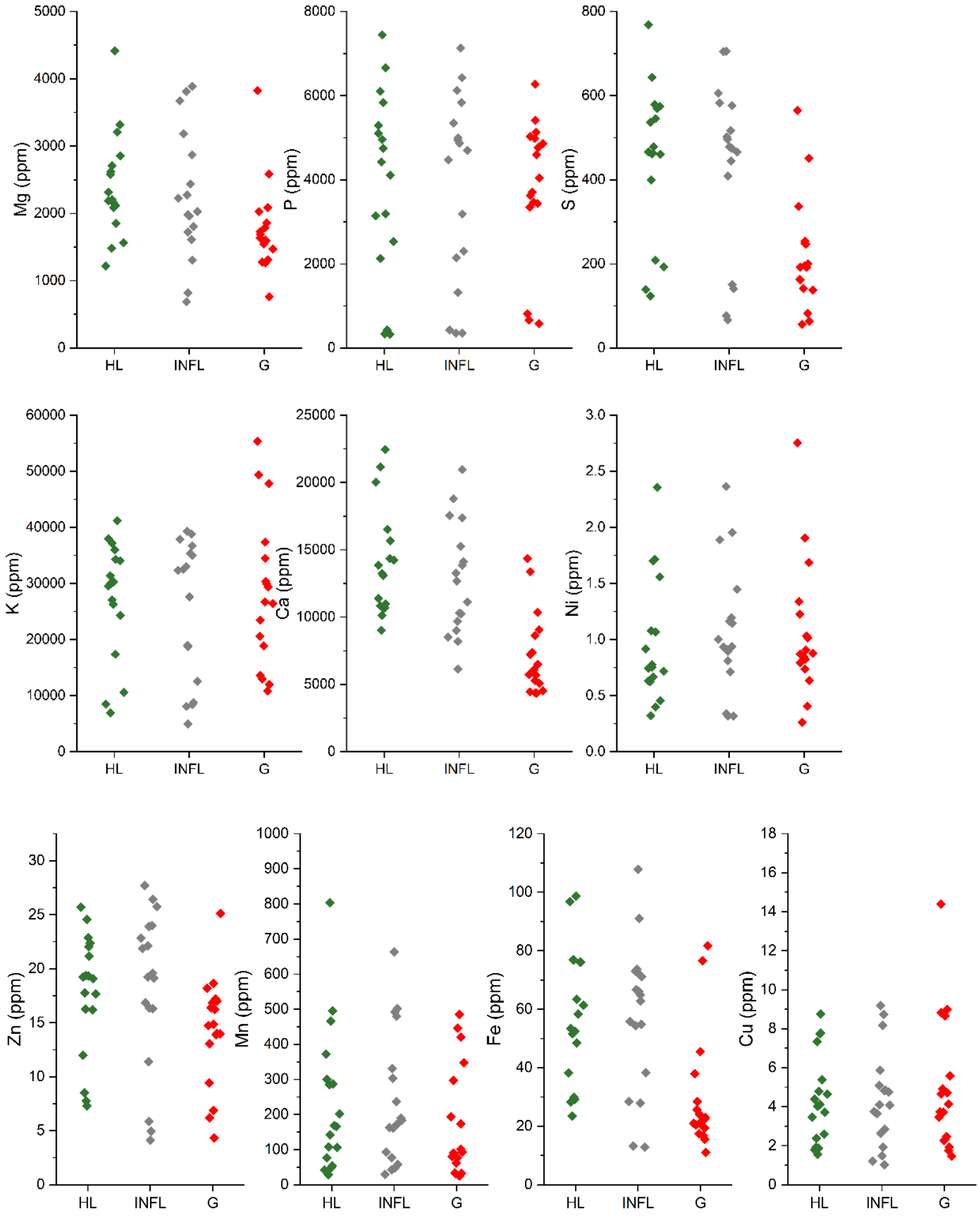

Fig. S3 Fold-change in the element concentrations in the tissues between galls and healthy leaves (G/HL), galls and infested leaf (G/INFL) and infested and healthy leaves (INFL/HL). The fold-change values were determined per tree and averaged. The values show average ± SE (n=18).

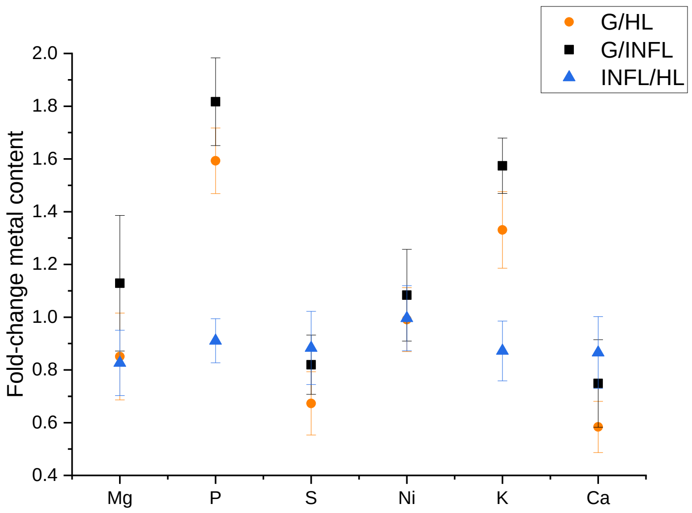

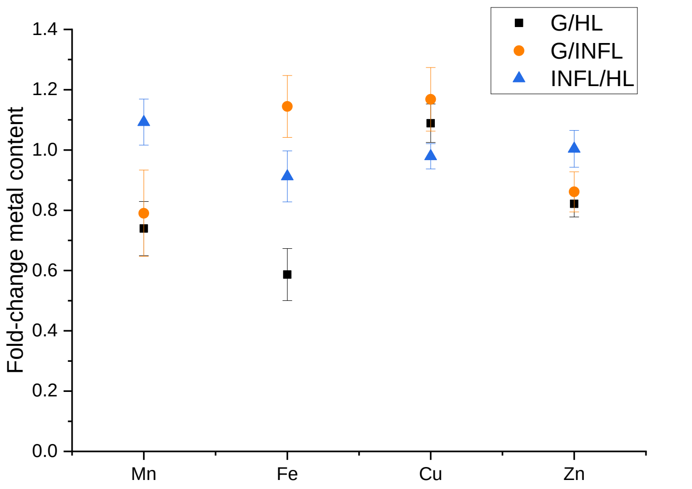

Fig. S4 Ultrastructure of *T. cordata* leaves, mature and young galls (A-H), galls (mature I-P, and young galls (R-U) obtained by TEM. A) cross section of a leaf showing epidermis, idioblasts, and mesophyll; B) abaxial epidermis with idioblasts, C) detail from the previous image showing enlarged epidermal cell, D) palisade cells, E) detail from previous image-chloroplast, F) spongy mesophyll, G) detail from previous image, H) chloroplast in spongy mesophyll. I) Mature gall cross section showing abaxial epidermis, parenchyma cells and vascular bundle. J) Detail of parenchyma showing large idioblasts, tannin deposits and raphide crystals, K) detail from the previous image, enlarged idioblast, L) cells in the nutritive tissues, M) abaxial epidermis showing tannin deposits, N) detail from the previous image, O) detail of vascular bundle, P) enlarged chlorophyll in the nutritive tissue showing large starch granules. R) young gall cross-section, S) detail from the previous image, abaxial epidermis and cell with large nuclei, T) middle of a young gall-future nutritive tissue, U) details from the previous image, disorganized chloroplasts with starch grains. Ecw-epidermal cell wall, ep-epidermis, c-cuticle, pa-palisade mesophyll, chl-chloroplast, g- grana (thylakoids), pg-plastoglobuli, sp-spongy mesophyll, pr-parenchyma, ta-tannin deposits, id-idioblasts, v-vacuole, vb- vascular bundle, s-starch, n-nucleus.

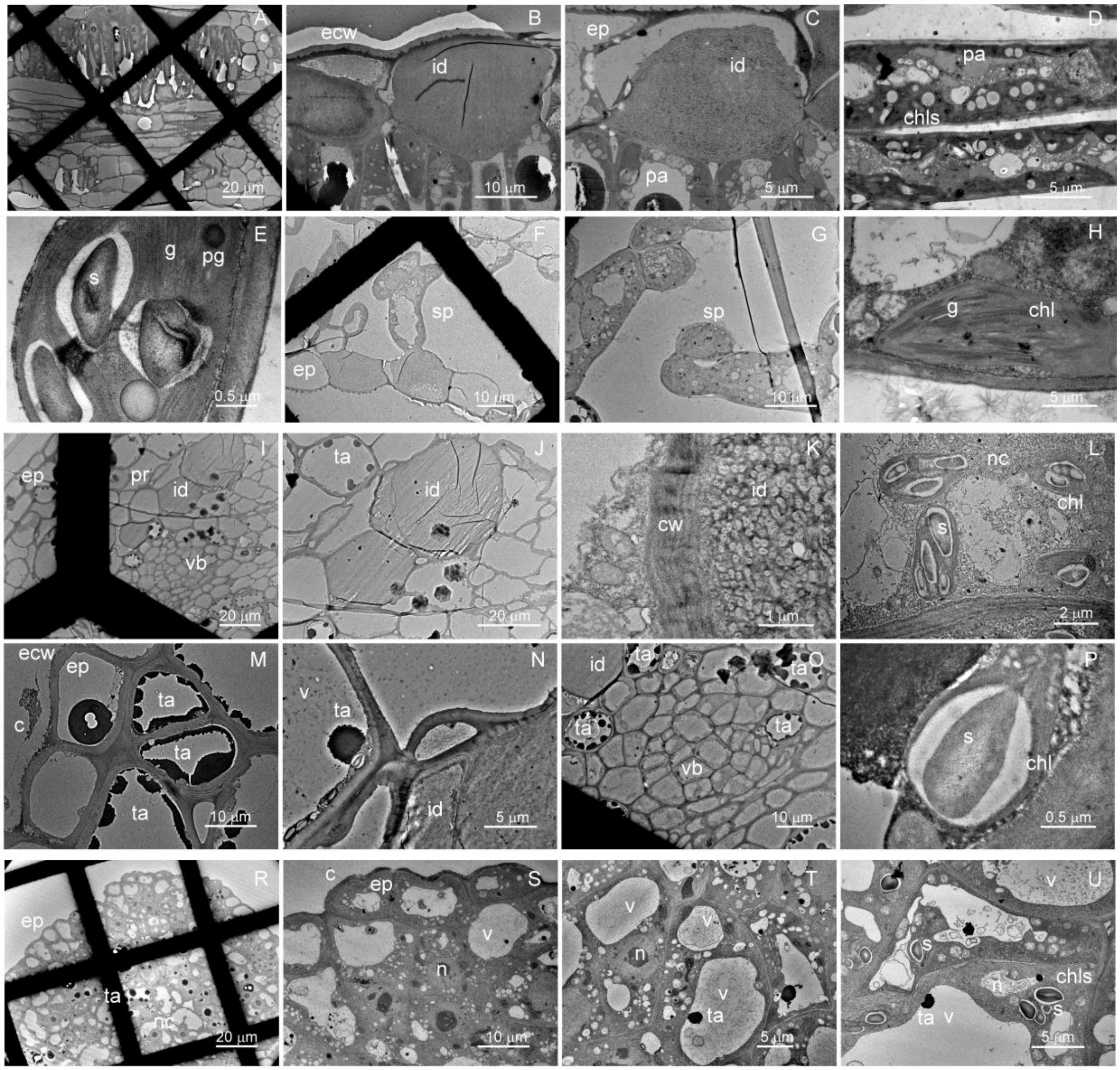

Fig. S5 Benchtop micro-XRF maps showing element distribution (Ca, Cu, Fe, K, Mn, Zn) in mature A) and young B) nail galls on *T. cordata* leaves. The colour scale is from low (black) to the maximum observed for each element (dark red).

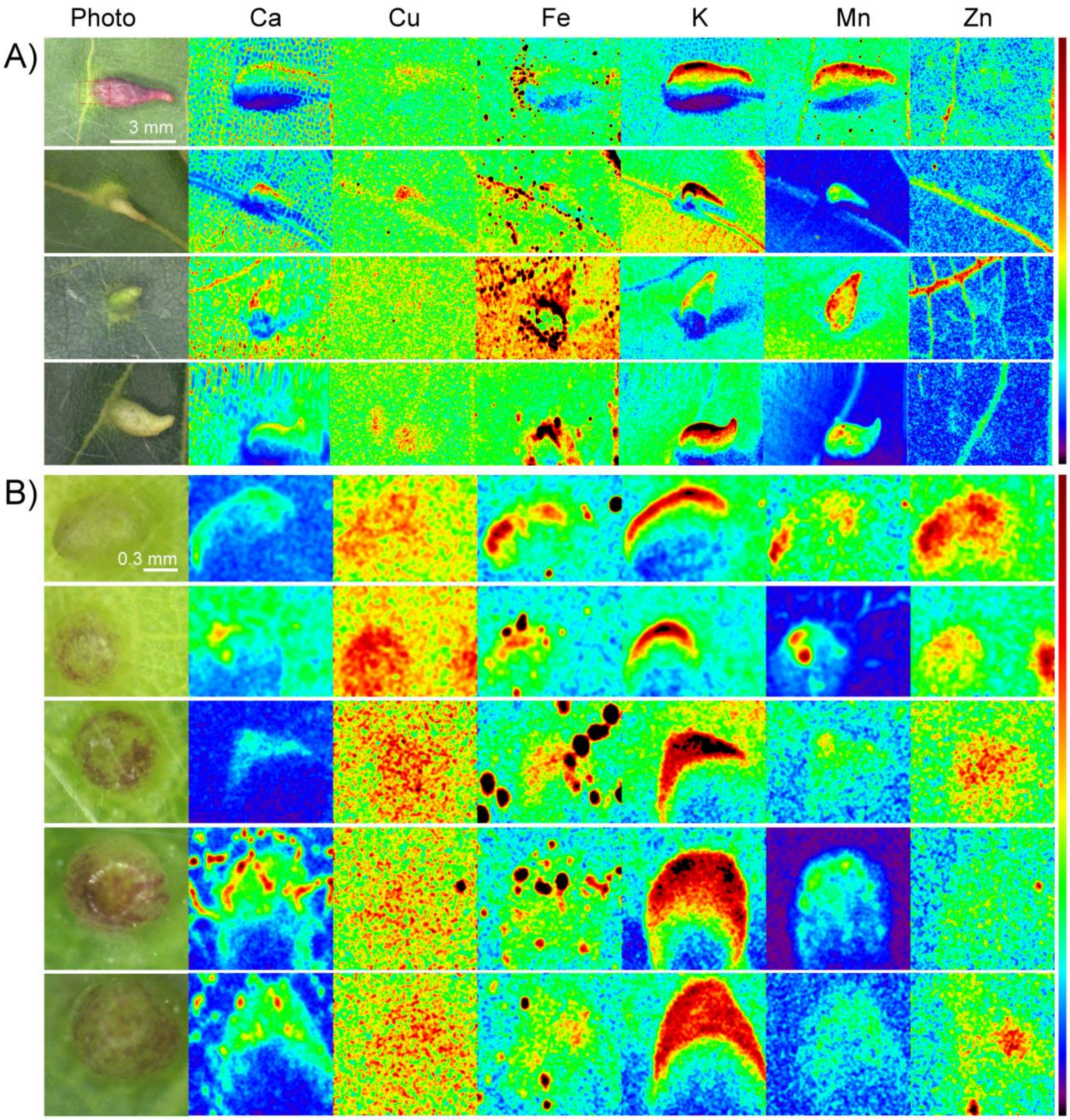

Fig. S6 Synchrotron µXRF tomography of a shock-frozen gall (replicate 2, sample RG3) showing absorption, and K, Ca, Mn, Fe, Ni, Cu and Zn distribution. The scale bar for K is from 0 to a relative upper end, and fully quantitative incl. absorption correction for Ca (0-500 mM), Mn (0-10 mM), Fe (0-1.5 mM), Ni (0-0.2 mM), Cu (0-0.2 mM) and Zn (0-0.3 mM). Inserts with a pointing arrow show enlarged details of gall tissues, in each case shown for the element where they are the most visible. Inserts in the left bottom corner show the tomograms of the leaf for the same elements and the same scale bars. The size scales in the lower right corner of the Ca and Cu tomograms refer to both the leaves and the main tomograms (not inserts) of the galls.

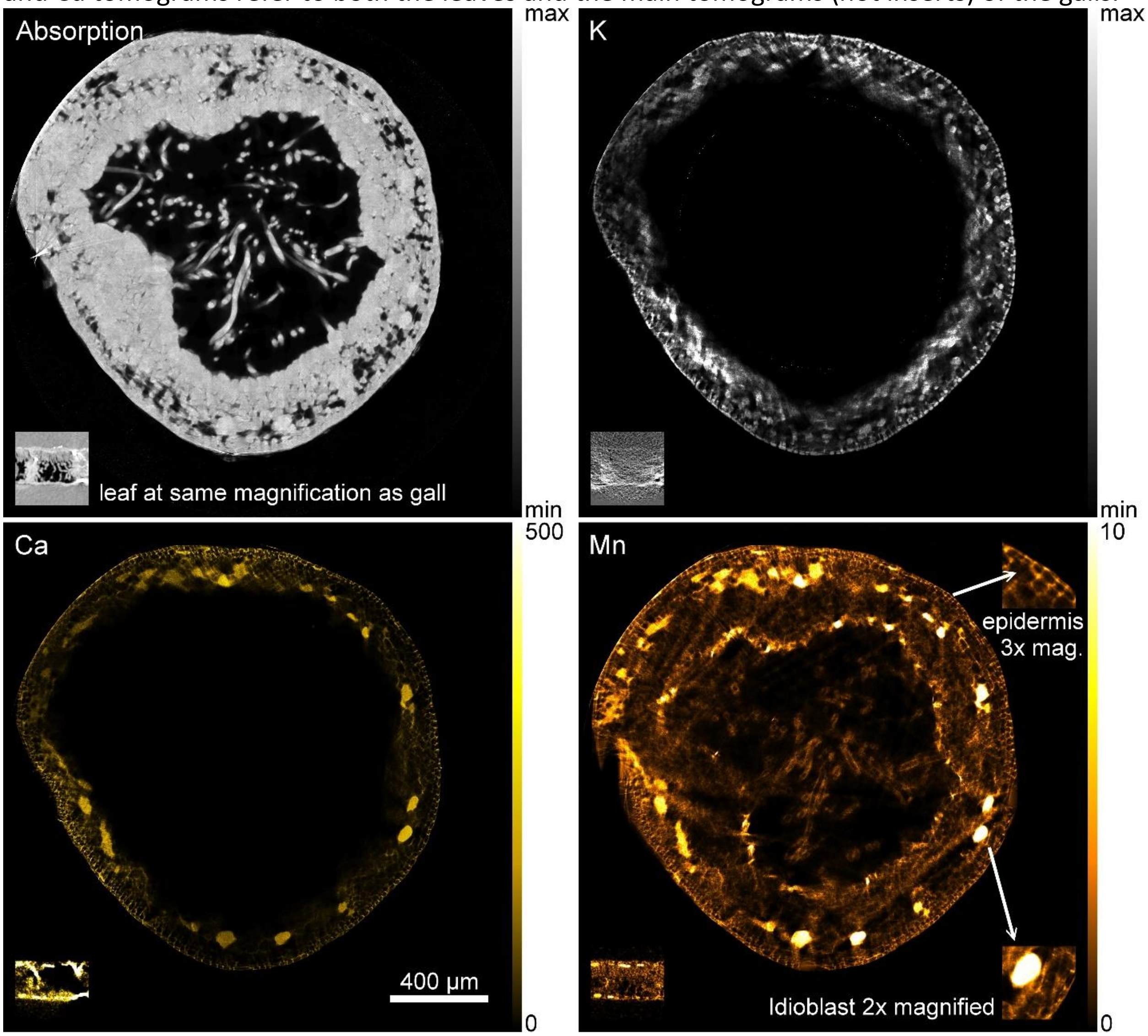

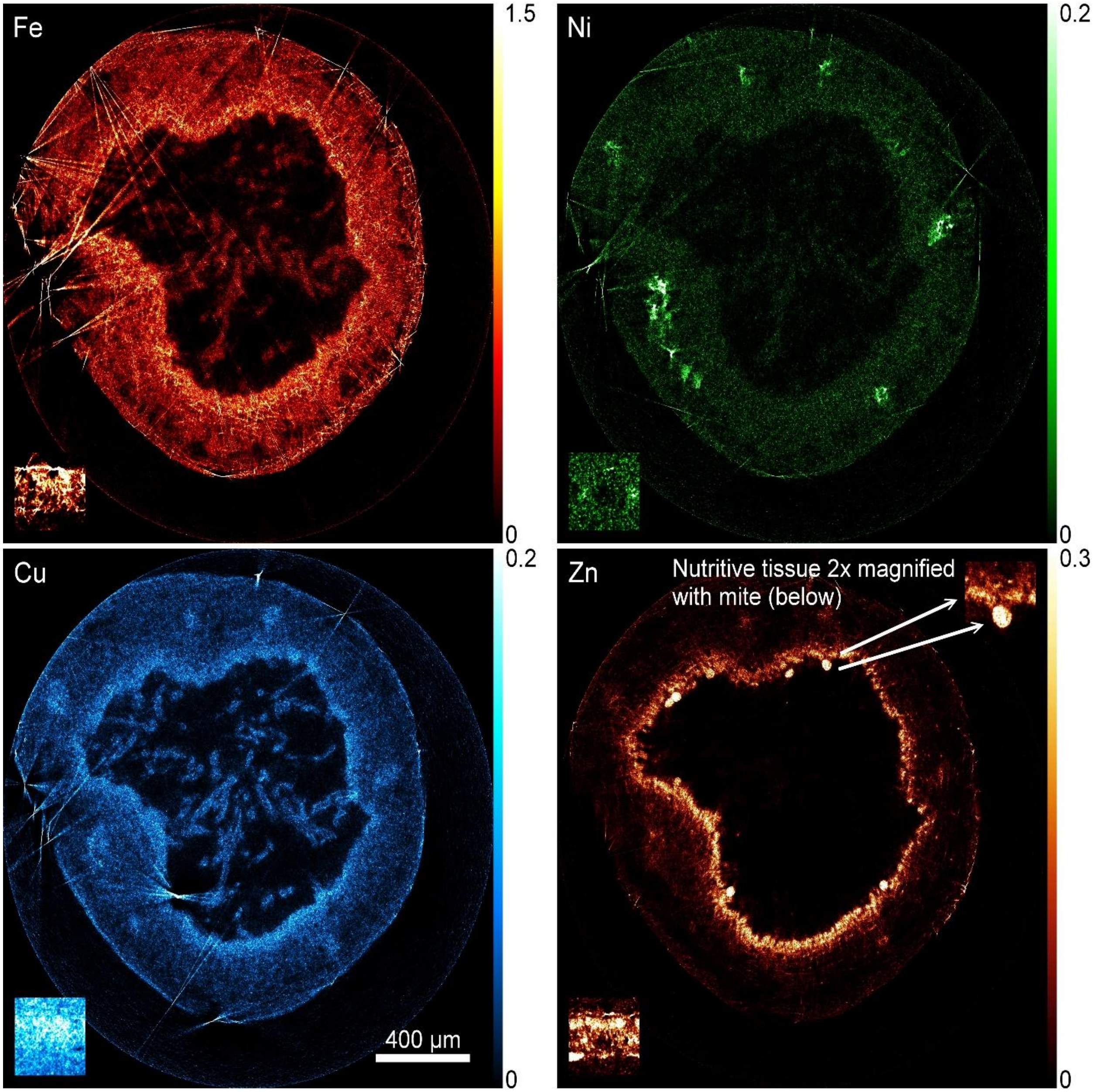

Fe
1.5
0
Ni
0.2
0
Cu
0.2
0
400 µm
Zn
Nutritive tissue 2x magnified
with mite (below)
0.3
0

Fig. S7 Synchrotron µXRF tomography of a shock-frozen gall (replicate 3, sample GG1) showing absorption, and K, Ca, Mn, Fe, Ni, Cu and Zn distribution. The scale bar for K is from 0 to a relative upper end, and fully quantitative incl. absorption correction for Ca (0-500 mM), Mn (0-10 mM), Fe (0-1.5 mM), Ni (0-0.2 mM), Cu (0-0.2 mM) and Zn (0-0.3 mM). Inserts with a pointing arrow show enlarged details of gall tissues, in each case shown for the element where they are the most visible. Inserts in the left bottom corner show the tomograms of the leaf for the same elements and the same scale bars. The size scales in the lower right corner of the Ca and Cu tomograms refer to both the leaves and the main tomograms (not inserts) of the galls.

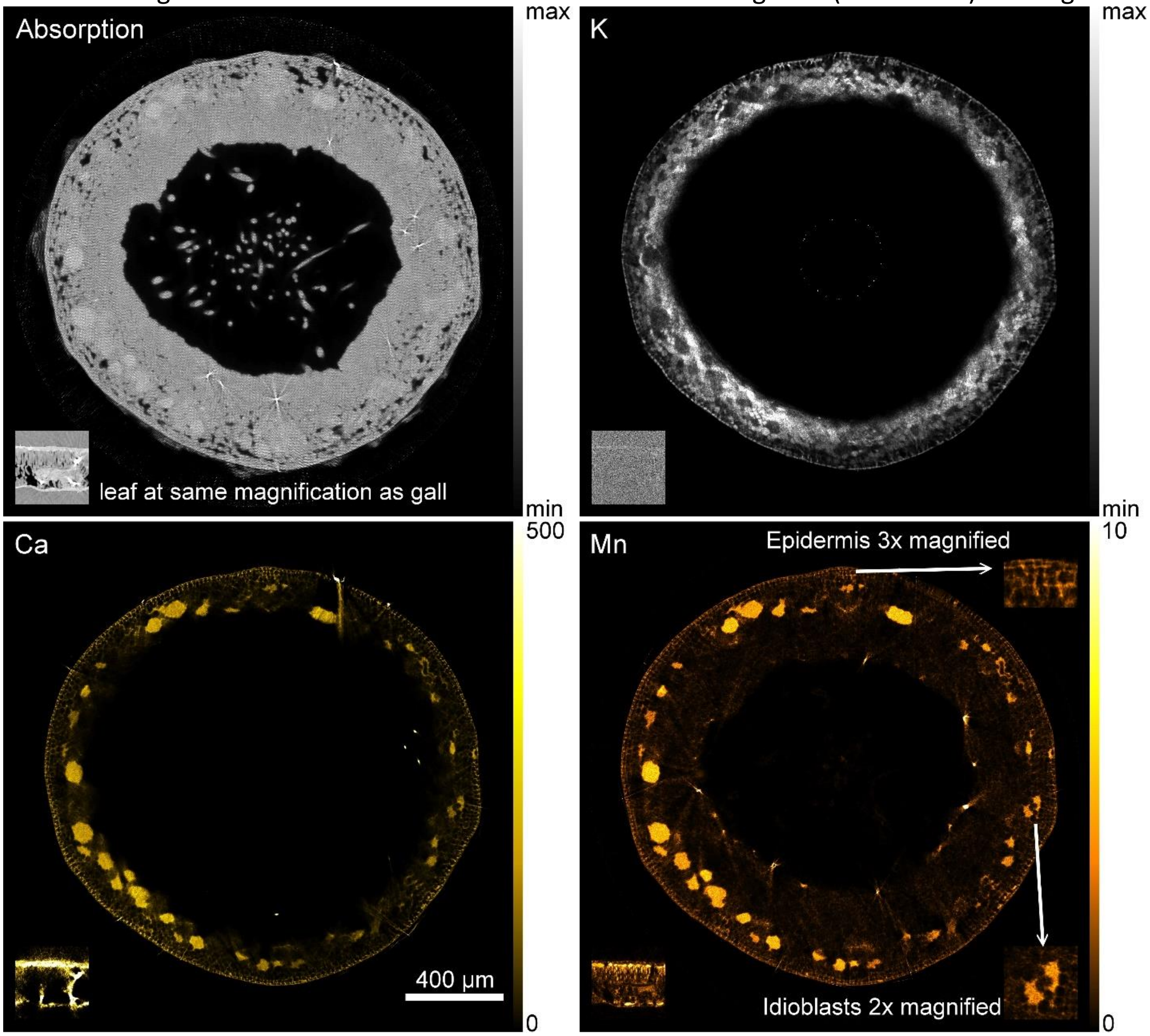

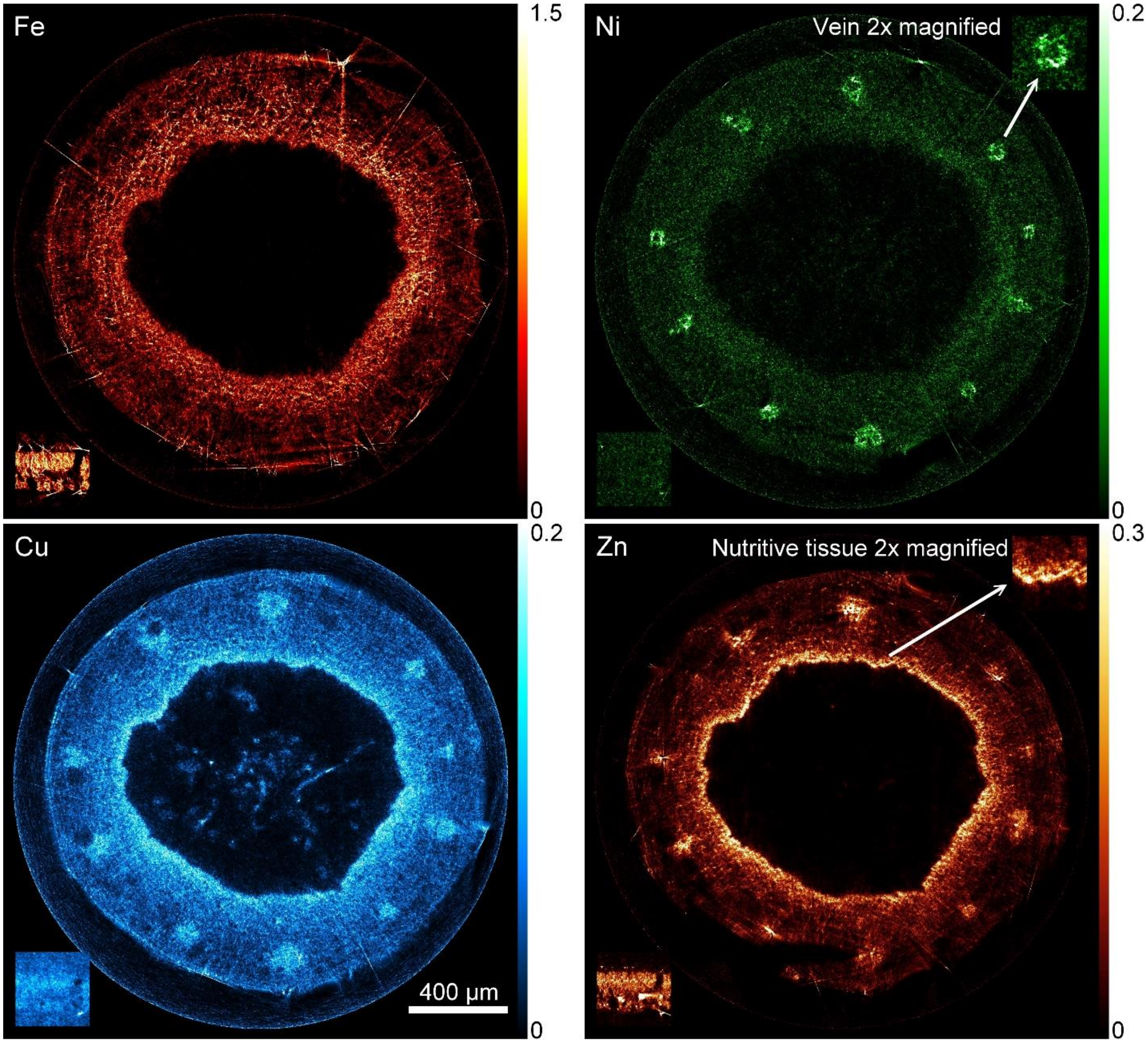

Fe
1.5
0
Ni
Vein 2x magnified
0.2
0
Cu
0.2
400 µm
0
Zn
Nutritive tissue 2x magnified
0.3
0

Fig. S8 Synchrotron µXRF tomography of shock-frozen leaves showing absorption, and K, Ca, Mn, Fe, Ni, Cu and Zn distribution. The scale bar for K is from 0 to a relative upper end, and fully quantitative incl. absorption correction for Ca (0-500 mM),Mn (0-10 mM), Fe (0-1.5 mM), Ni (0-0.2 mM), Cu (0-0.2 mM) and Zn (0-0.3 mM).

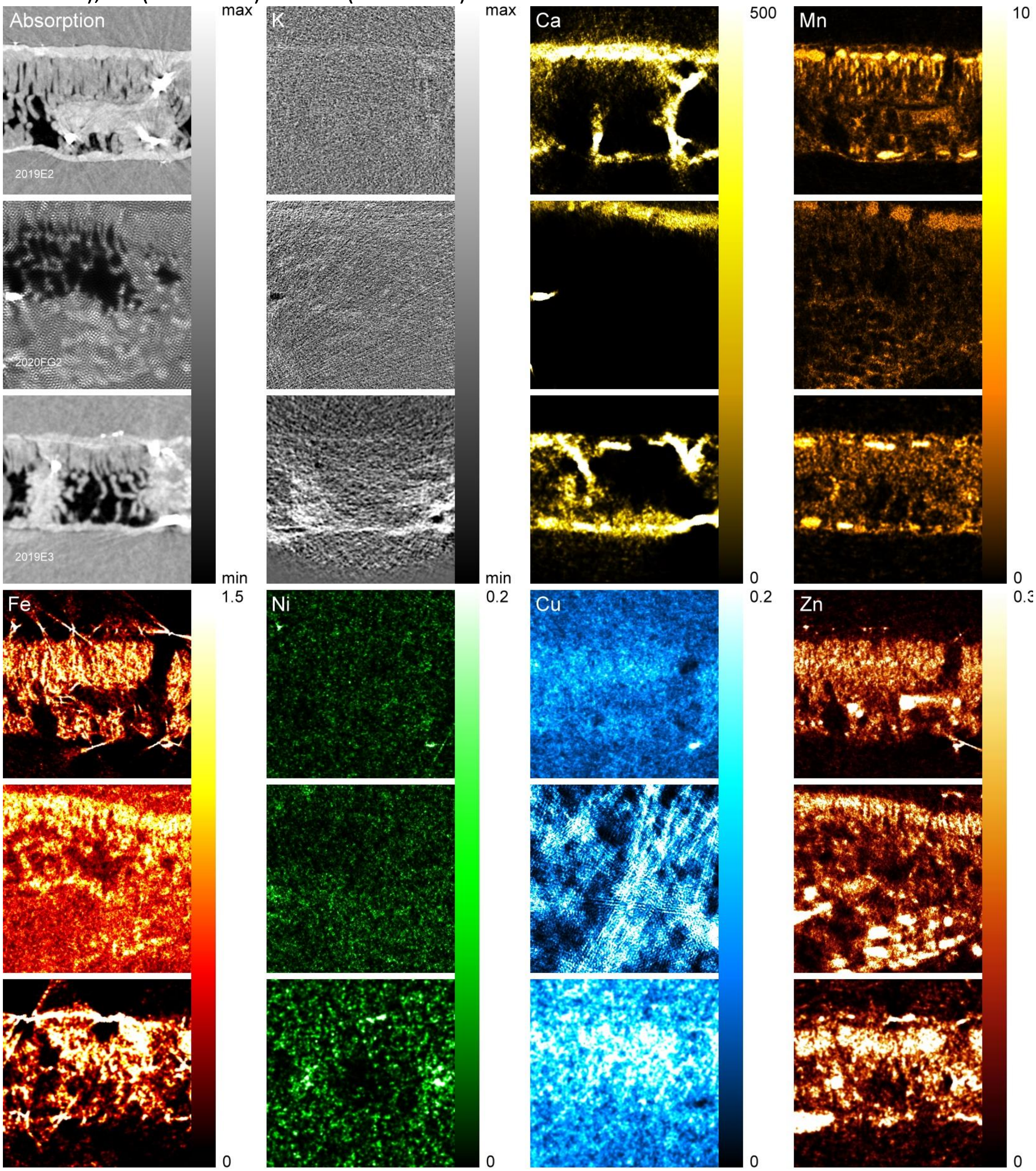

Fig. S9. µXANES linear combination fits of all plant samples analysed for this work. The equation used for all was the following, the columns 2-12 contained the data of the models to be fitted to the spectrum of the biological sample:
f = offset + slope * x + thylakoids*col(2) + Mn2mucilage*col(4) + Mn2malate*col(5) + Mn2aquo*col(7) + Mn2phytate*col(9) + Mn2GSH*col(10) +Mn3acetate*col(11) +Mn4oxide*col(12)
with the constraint that all components had to be >0

A) The XANES spectra of all model compounds after data reduction (background removal and normalisation)

B) Each panel shows the LCF with the original data, fitted curve (=prediction) and residuals of one tissue of one biological replicate

A) All models

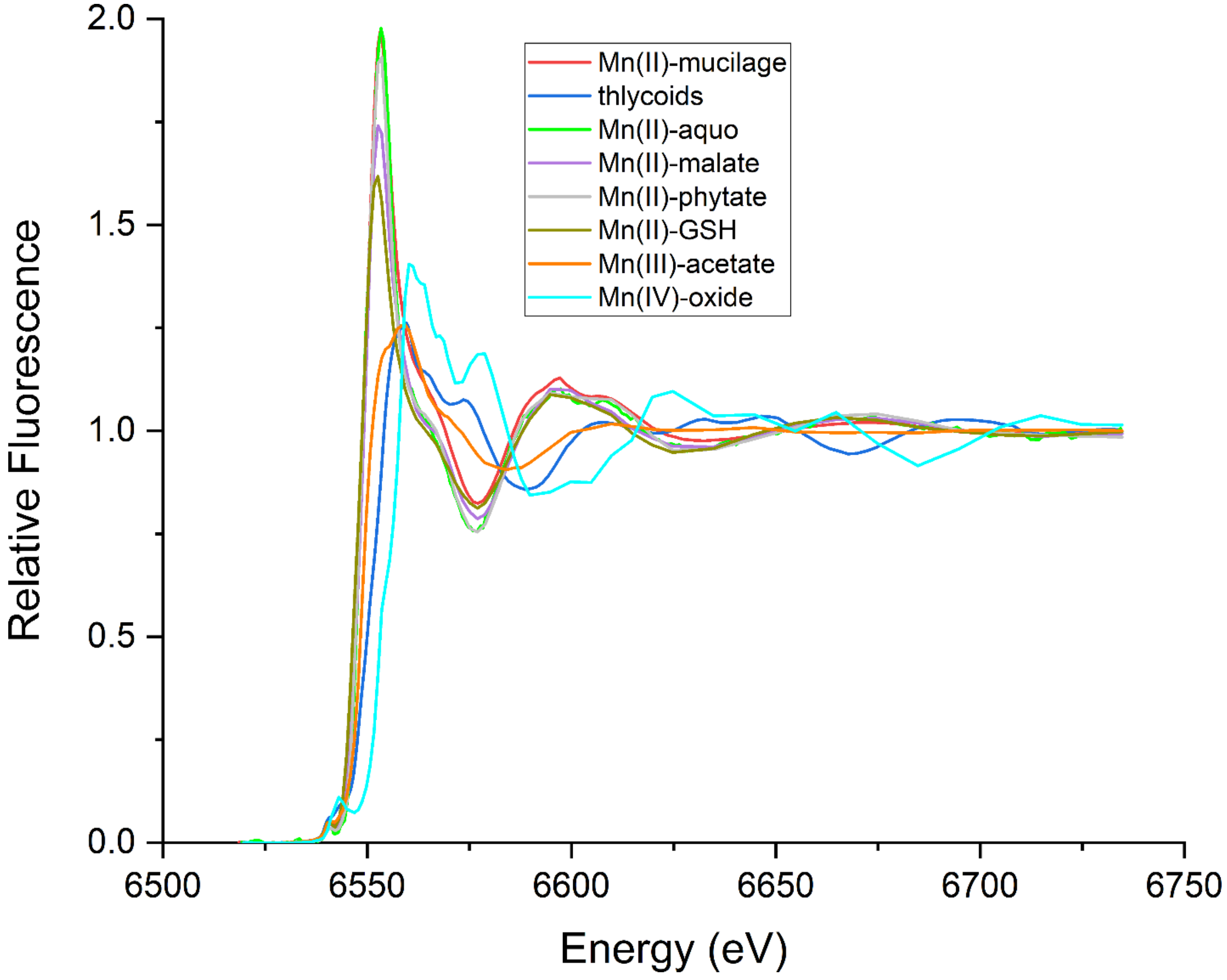

Relative Fluorescence
Energy (eV)
0.0
0.5
1.0
1.5
2.0
6500
6550
6600
6650
6700
6750
Mn(II)-mucilage
thlycoids
Mn(II)-aquo
Mn(II)-malate
Mn(II)-phytate
Mn(II)-GSH
Mn(III)-acetate
Mn(IV)-oxide

B) LCF of biological samples

Gall 2, epidermis

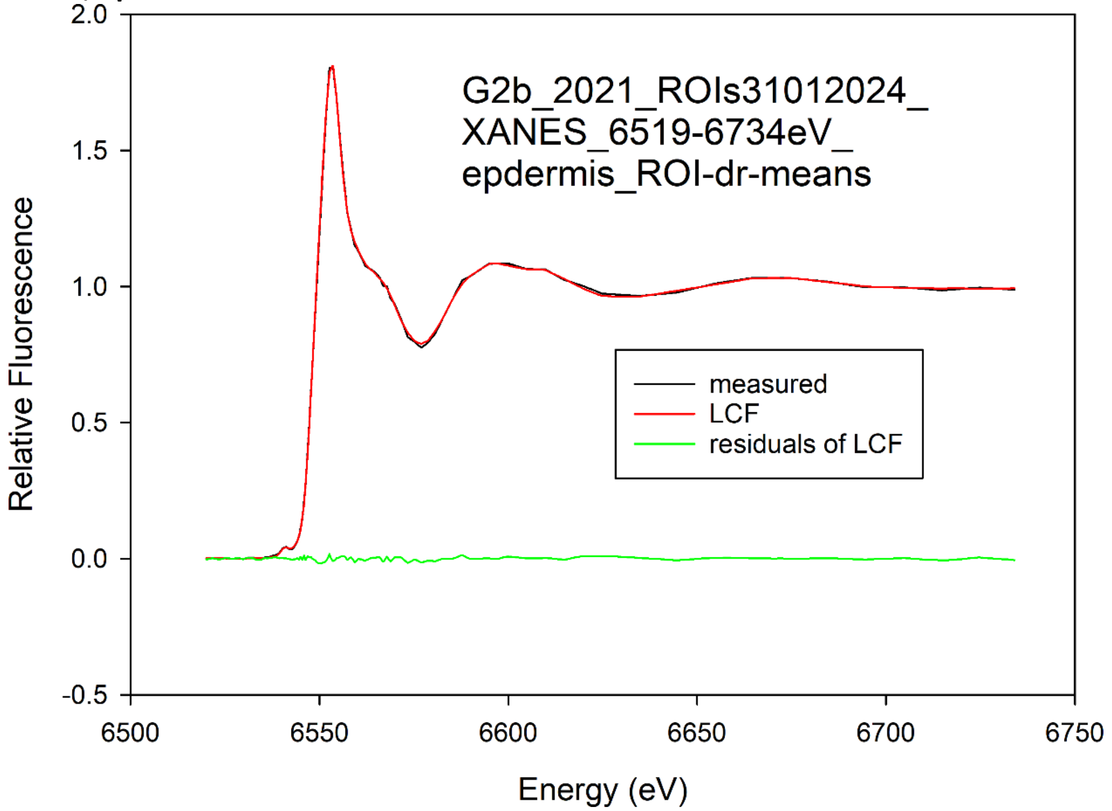

Gall 2, idioblasts

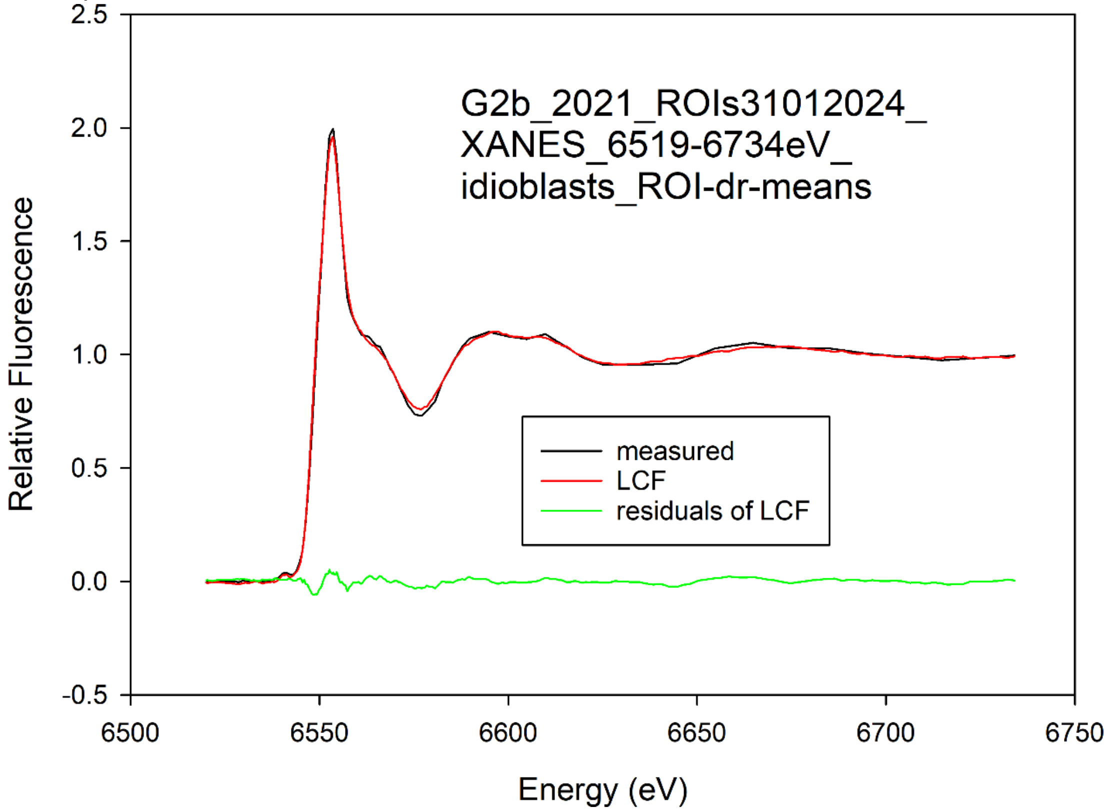

Gall2, nutritive tissue

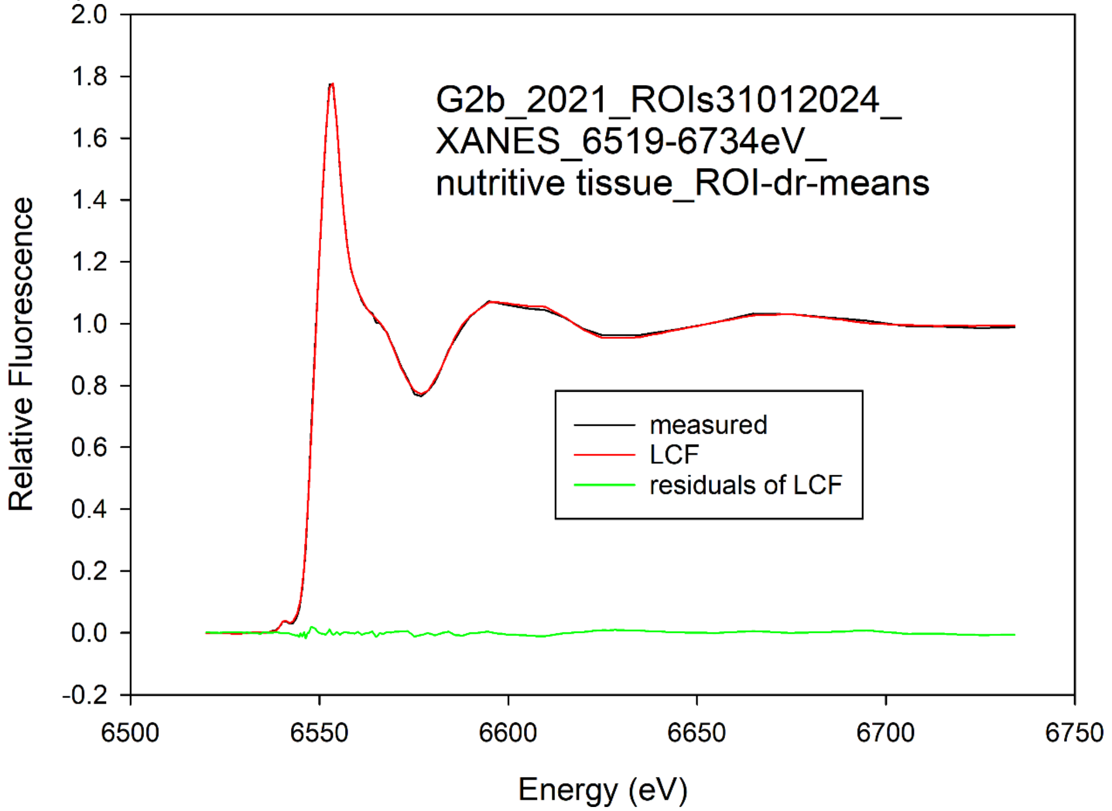

Gall 2, parenchyma

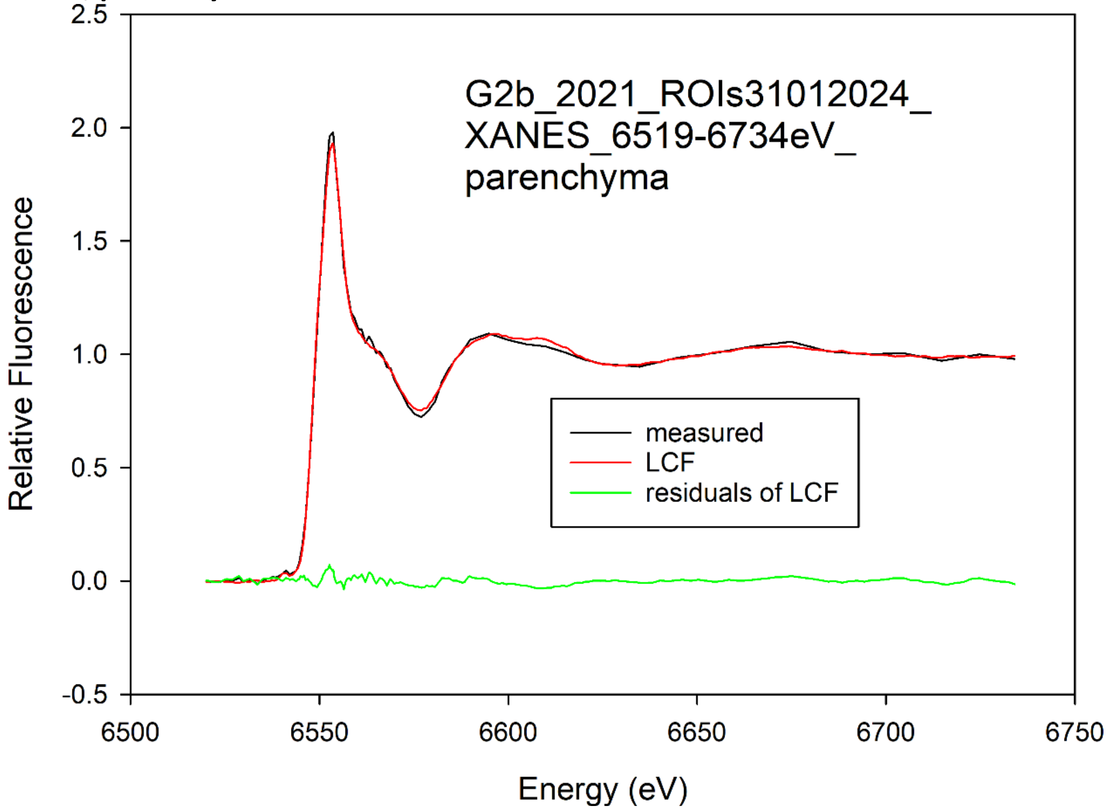

Gall 3, epidermis

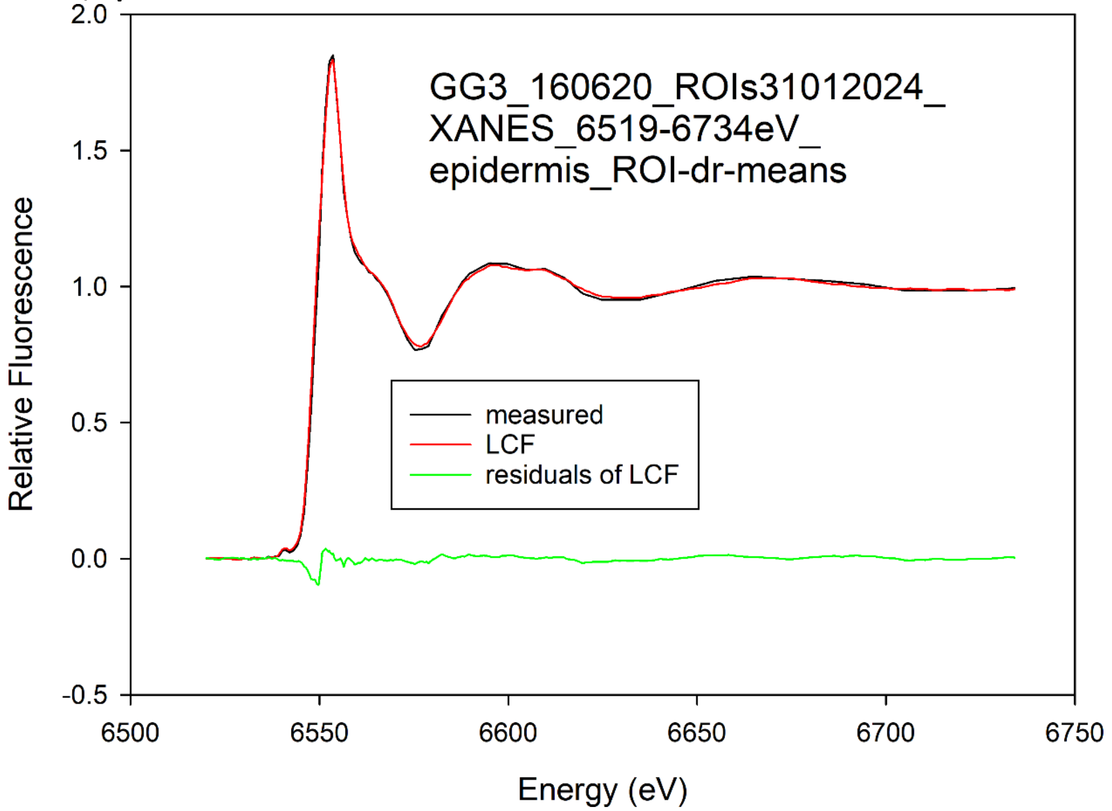

Gall 3, idioblasts

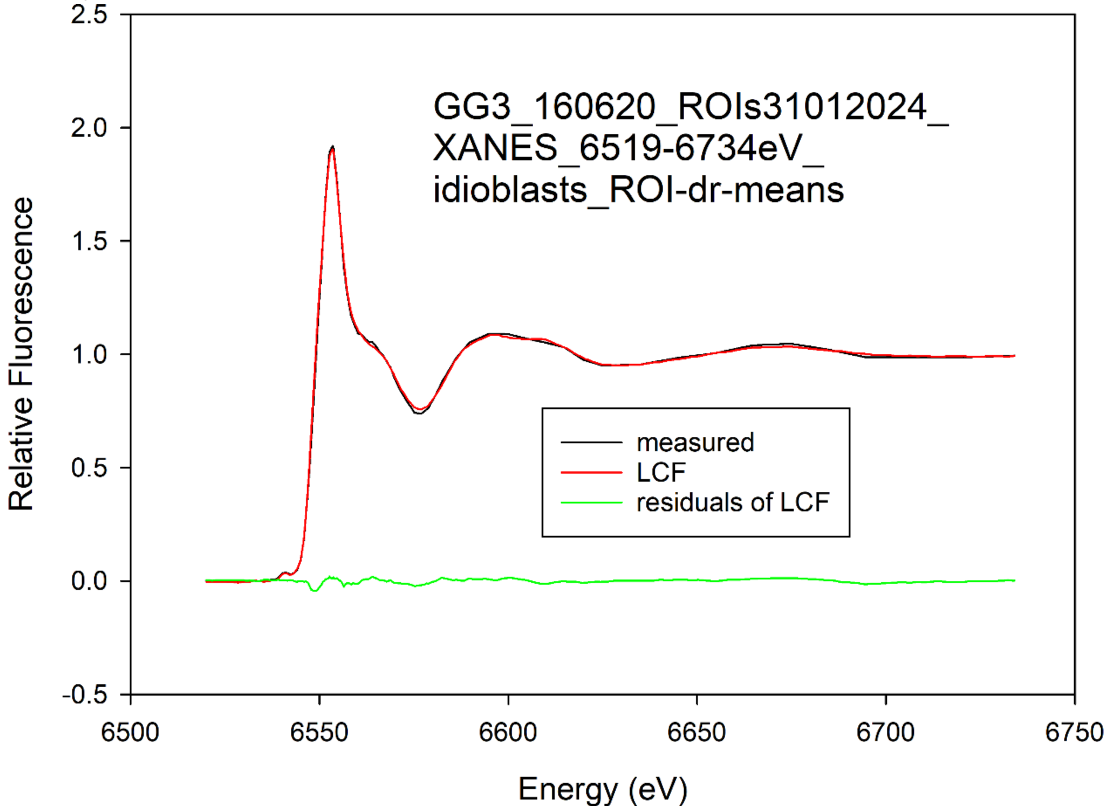

Gall 3, nutritive tissue

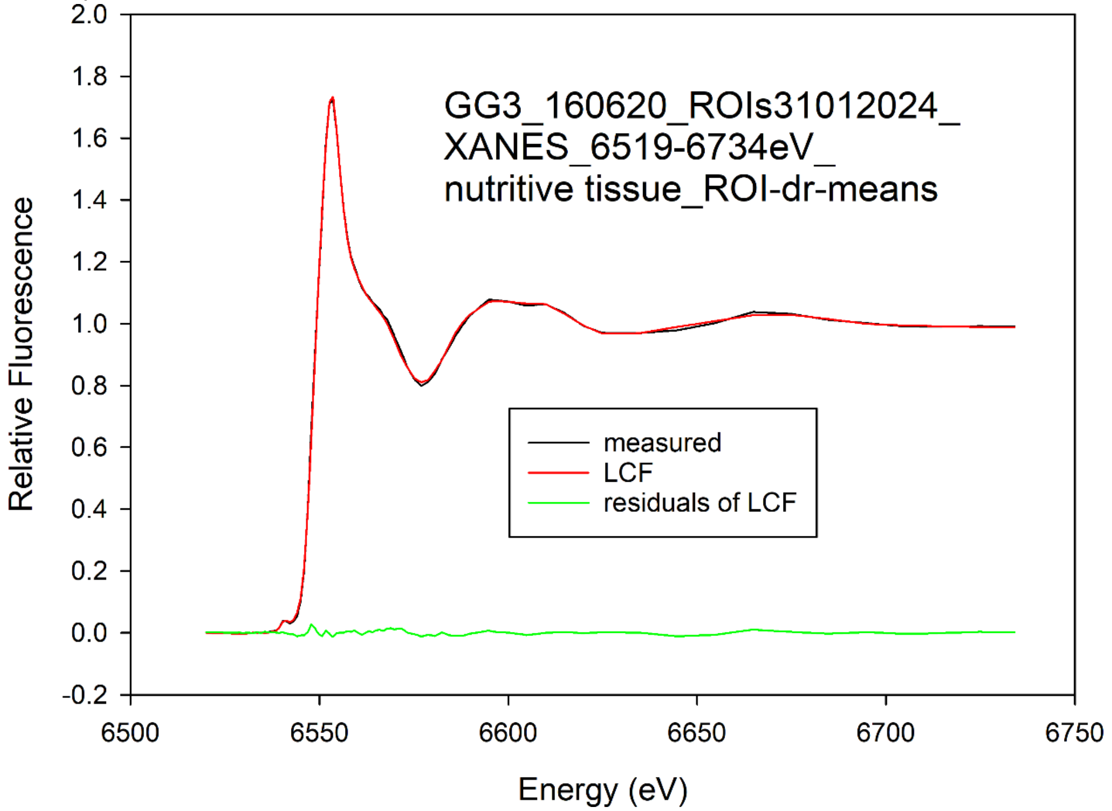

Gall 3, parenchyma

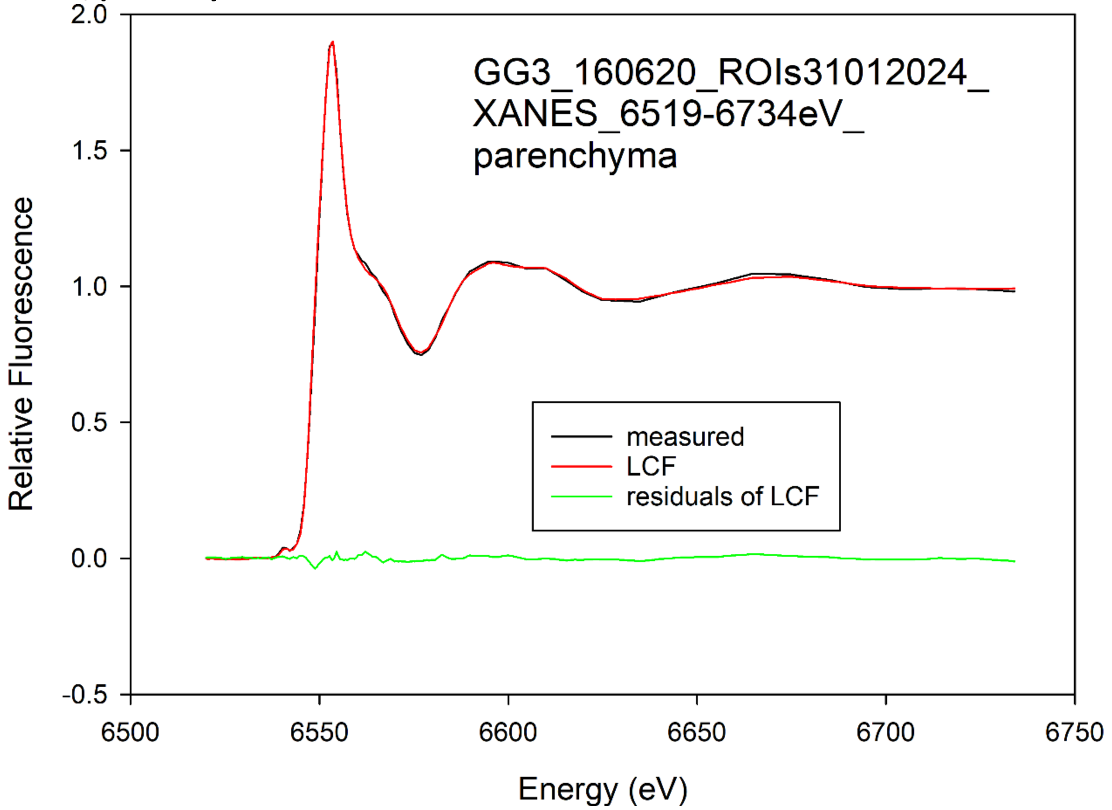

Gall 4, epidermis

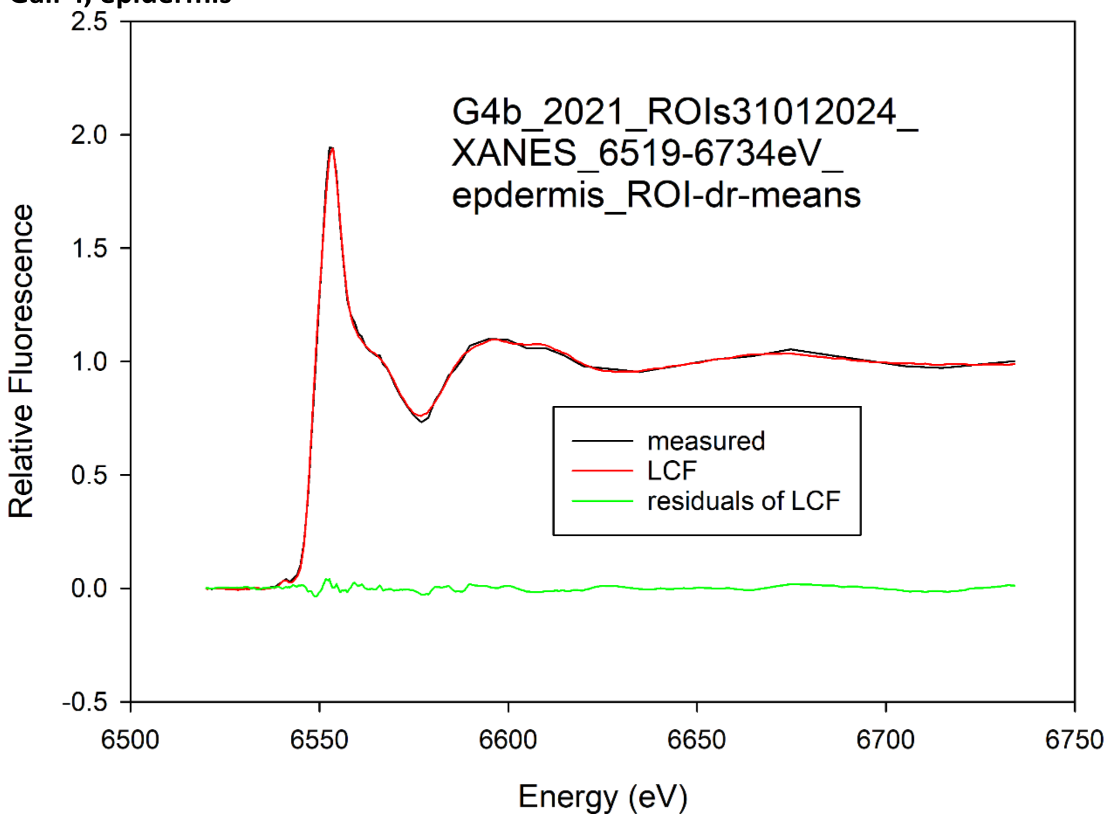

Gall 4, idioblasts

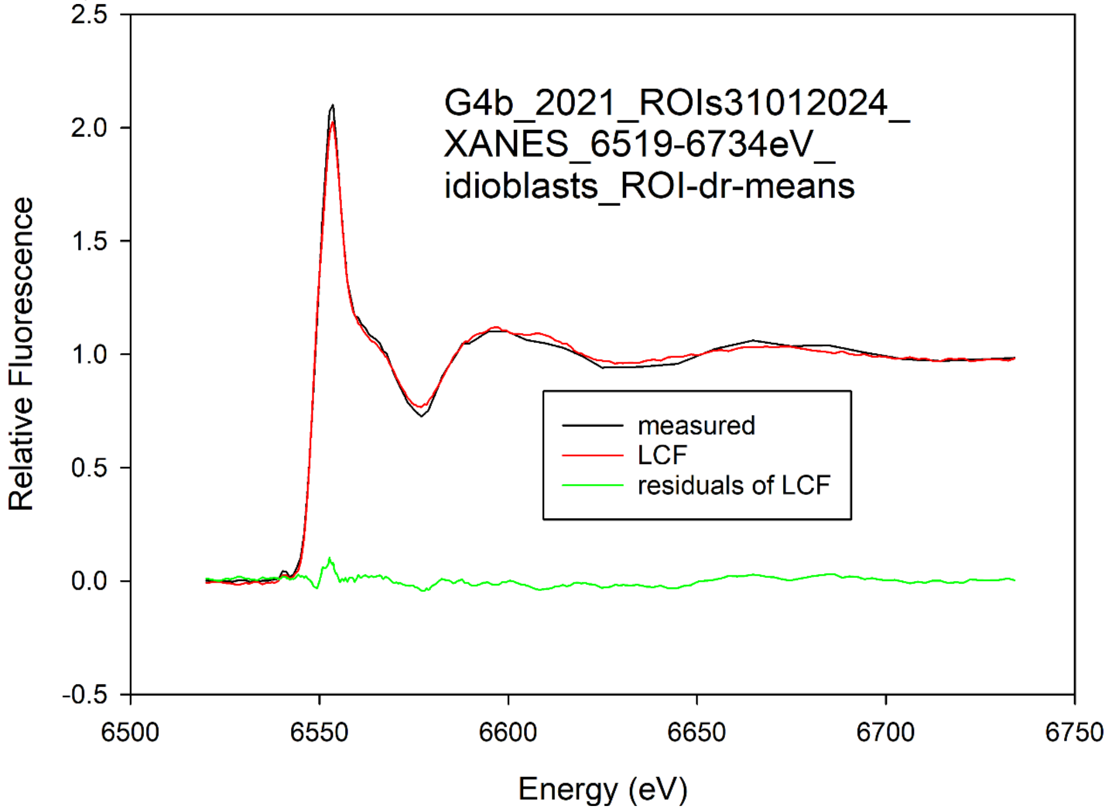

Gall 4, nutritive tissue

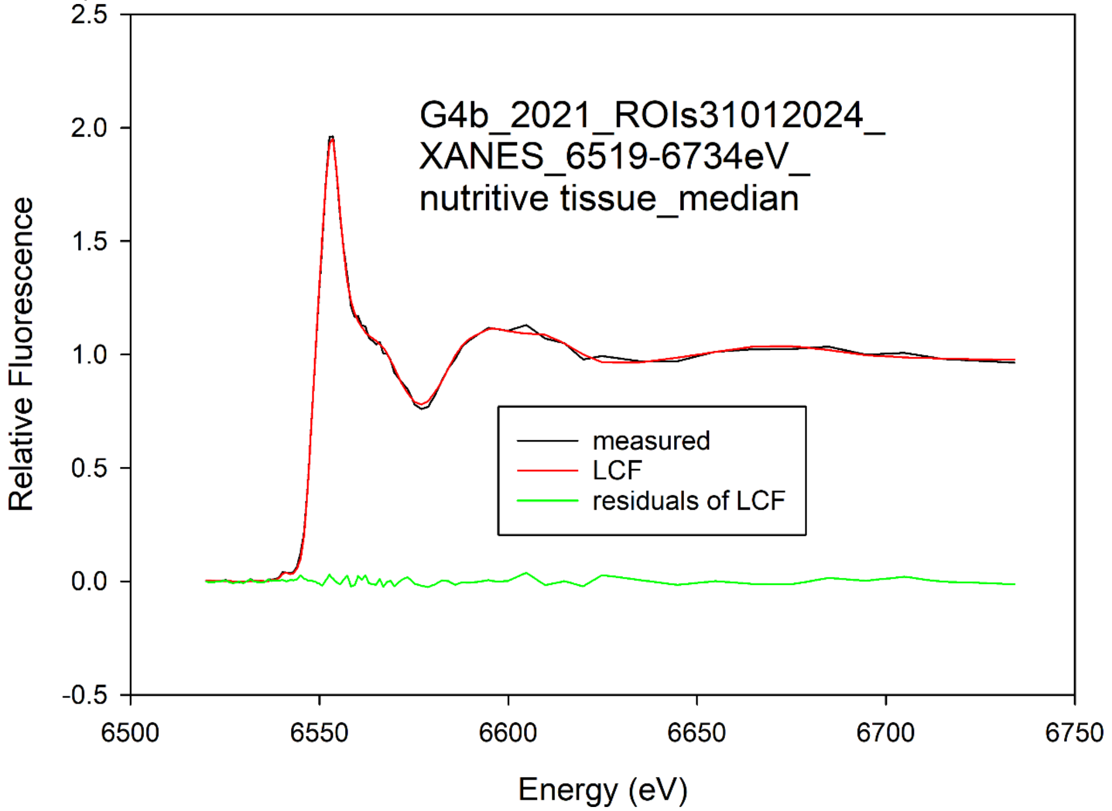

Gall 4, parenchyma

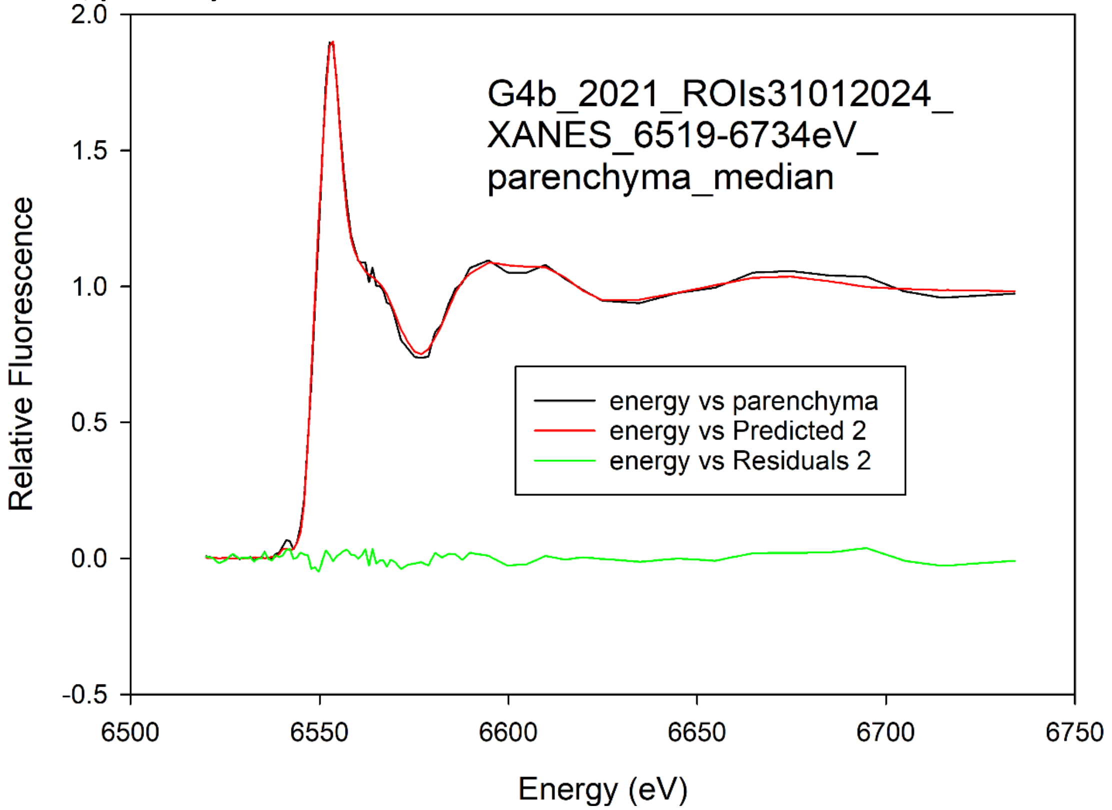

Healthy leaf 1

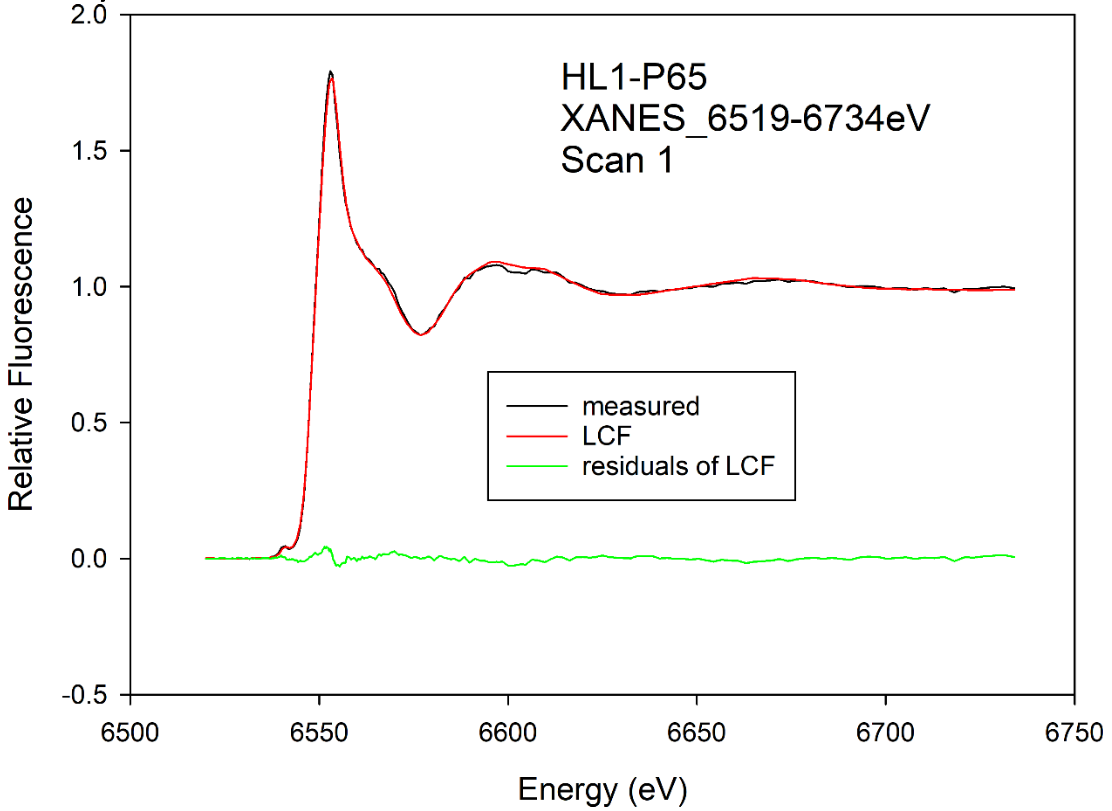

Healthy leaf 2

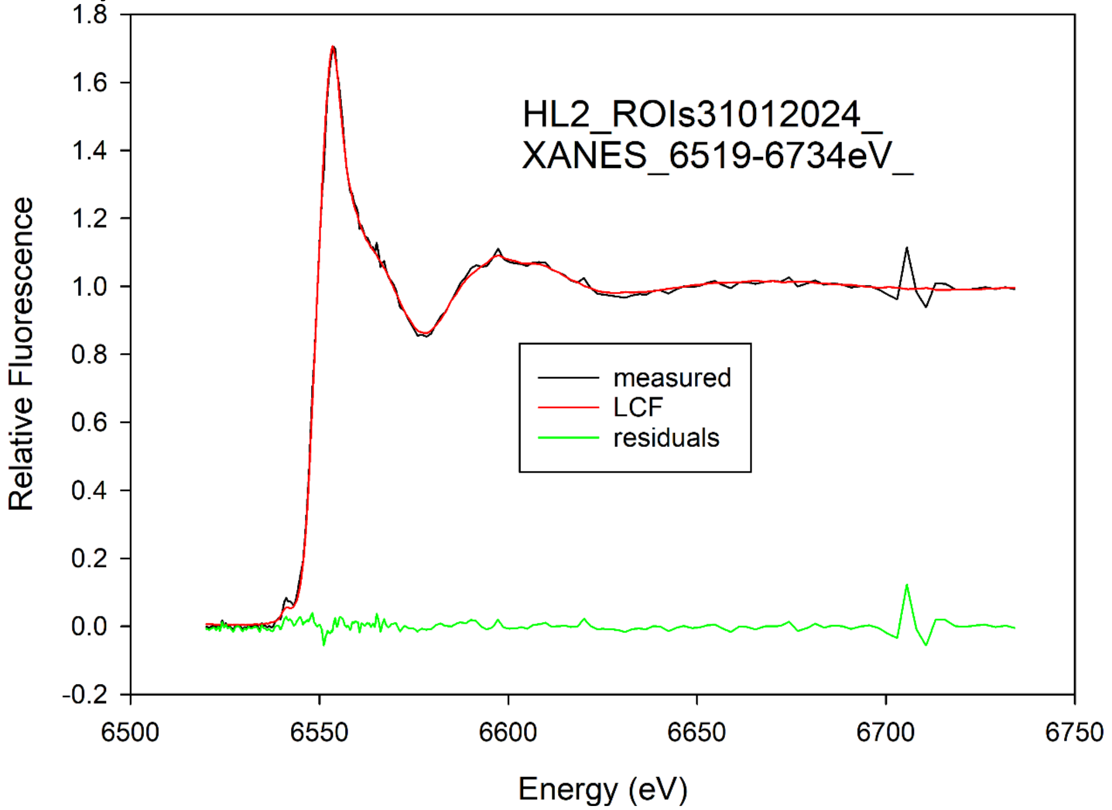

Healthy leaf 3

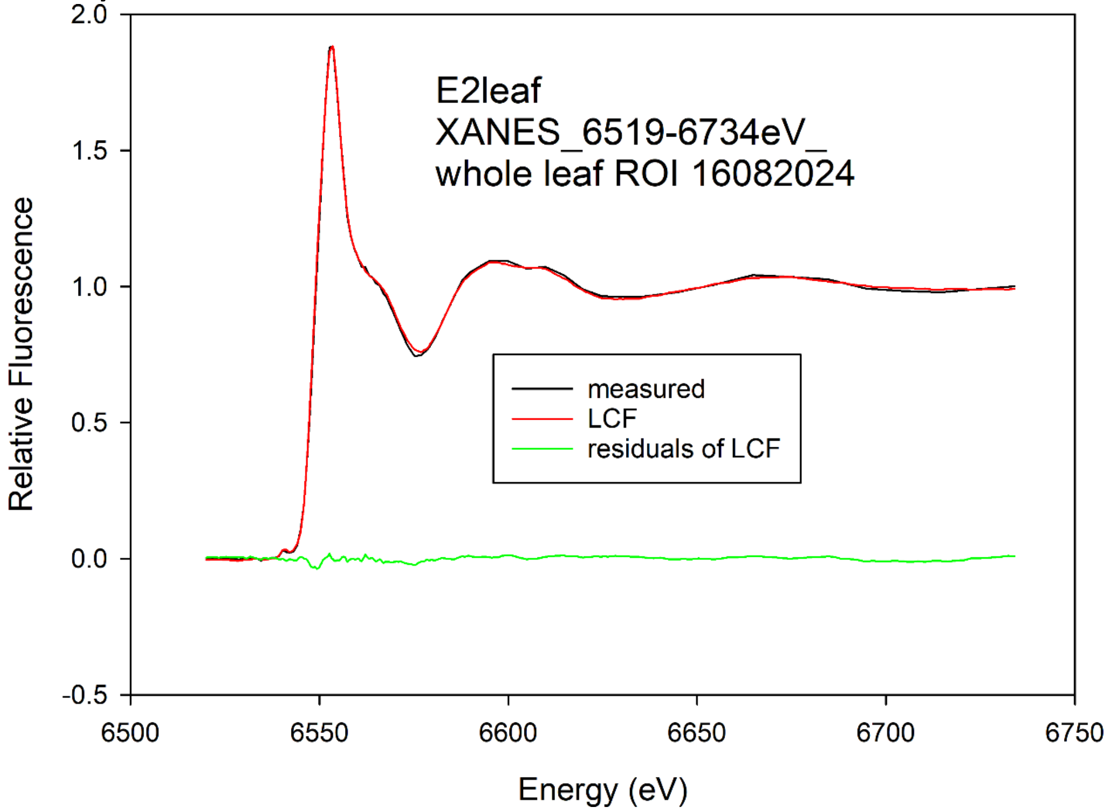

Fig. S10 Volcano plot of DEGs with padj < 0.05 in the infested leaves compared to healthy ones (n=4).

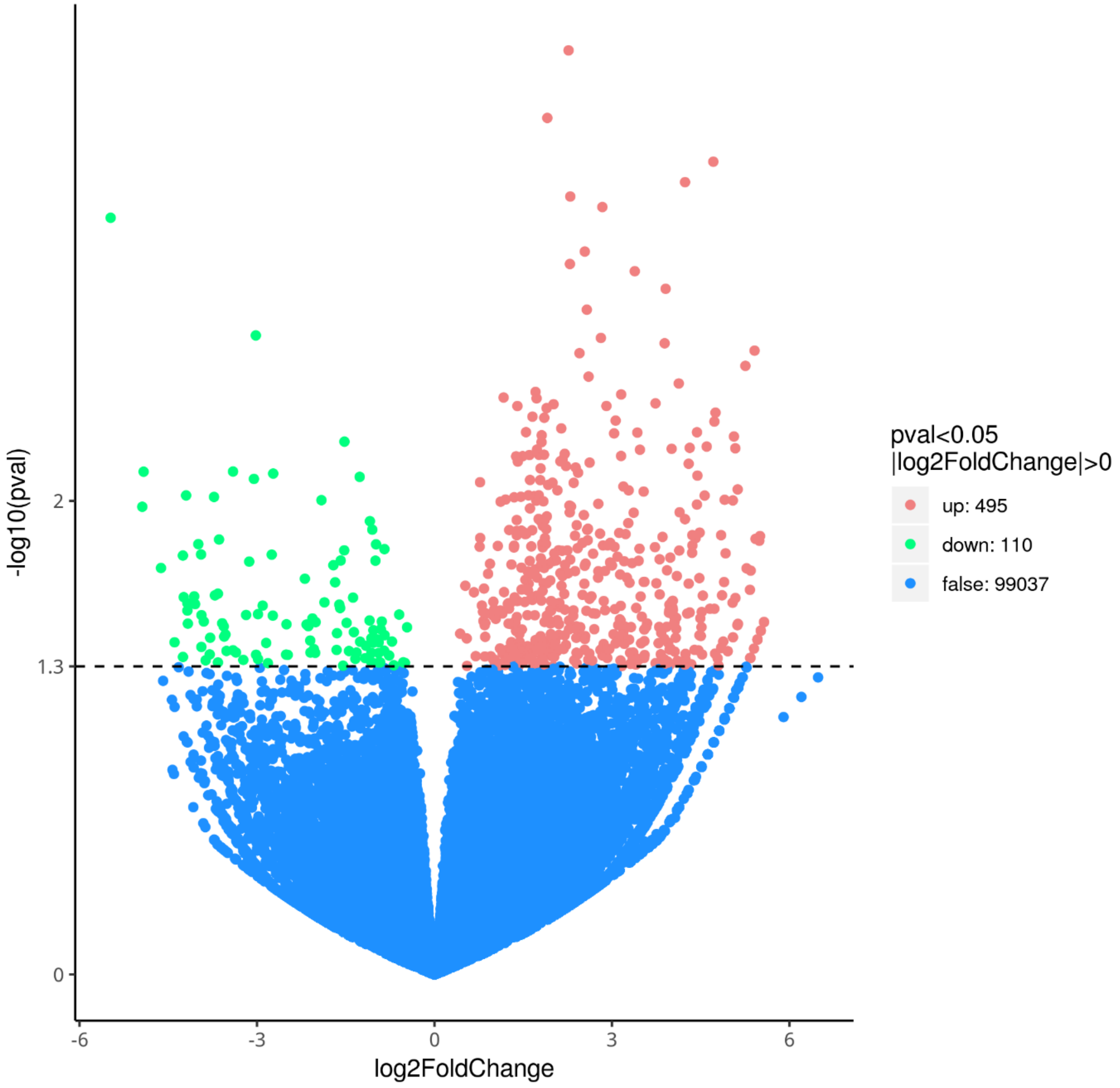

Table S1 Concentrations of bioavailable elements in the topsoil (0-40 cm depth at three locations in which infested T. cordata trees were growing. The data present averages ± SE (n=3).

Soil	cm	Ca (mg kg^{-1})	Cu (µg kg^{-1})	Fe (µg kg^{-1})	K (mg kg^{-1})	Mg (mg kg^{-1})	Mn (µg kg^{-1})	Ni (µg kg^{-1})	P (µg kg^{-1})	S (µg kg^{-1})	Zn (µg kg^{-1})	pH
1	10	171.06 ± 6.03	55.26 ± 2.54	71.49 ± 2.86	2519.67 ± 110.74	24.34 ± 0.81	3817.59 ± 155.13	17.43 ± 0.80	121.41 ± 2.01	446.73 ± 11.12	1364.65 ± 49.24	5.75
	20	205.01 ± 6.06	35.97 ± 0.71	76.22 ± 1.56	830.80 ± 18.24	27.09 ± 0.20	1464.95 ± 9.86	25.51 ± 0.23	91.58 ± 2.53	355.42 ± 7.82	1018.22 ± 2.81	5.82
	30	152.96 ± 0.75	30.12 ± 0.59	41.80 ± 1.48	616.37 ± 3.44	27.14 ± 0.69	1074.40± 36.99	22.62 ± 0.72	32.10 ± 0.98	209.33 ± 3.17	1156.10 ± 33.58	6.14
	40	94.11 ± 1.02	30.34 ± 0.76	25.71 ± 0.80	592.89 ± 8.88	17.20 ± 0.12	889.25 ± 30.64	10.38 ± 0.16	56.31 ± 1.49	158.42 ± 5.55	481.80 ± 6.93	6.09
2	10	363.41 ± 5.87	73.74 ± 2.68	120.97 ± 3.01	1885.48 ± 27.64	31.13 ± 0.54	891.11 ± 5.79	10.72 ± 0.09	84.61 ± 3.03	612.55 ± 12.02	510.08 ± 2.40	6.75
	20	81.84 ± 1.38	88.10 ± 2.30	100.50 ± 0.74	514.23 ± 11.73	15.70 ± 0.25	1197.86 ± 10.70	25.85 ± 0.57	30.00 ± 0.12	541.33 ± 3.61	551.90 ± 5.45	5.14
	30	85.68 ± 1.91	58.52 ± 3.80	105.15 ± 1.73	409.50 ± 4.20	13.07 ± 0.23	1652.08 ± 6.09	29.08 ± 0.35	37.87 ± 0.28	659.33 ± 8.07	461.32± 2.88	4.97
	40	299.08 ± 0.90	51.93 ± 2.40	81.72 ± 1.84	1099.87 ± 9.28	20.31 ± 0.65	1722.47 ± 52.12	24.39 ± 0.35	70.92 ± 0.74	680.01 ± 15.05	562.07 ± 26.39	6.55
3	10	447.89 ± 2.58	59.49 ± 1.90	115.296 ± 5.15	2923.84 ± 44.52	42.72 ± 1.19	357.10 ± 14.65	16.213 ± 0.38	560.13 ± 8.89	888.05 ± 11.86	861.79 ± 13.17	7.53
	20	410.66 ± 4.49	44.19 ± 1.83	54.89 ± 0.53	991.83 ± 12.32	30.74 ± 0.48	328.17 ± 11.54	11.18 ± 0.10	318.19 ± 2.79	746.12 ± 12.74	481.19 ± 7.22	7.24
	30	454.95 ± 3.63	82.19 ± 1.50	54.91 ± 0.32	613.11 ± 7.36	17.39 ± 0.13	340.26 ± 4.74	12.35 ± 0.38	332.14 ± 3.76	1323.82 ± 14.77	2957.32 ± 69.77	7.7
	40	209.54 ± 1.33	38.11 ± 0.79	35.88 ± 0.95	643.51± 1.57	12.48 ± 0.11	596.81 ± 6.97	11.34 ± 0.37	158.94 ± 2.38	625.36 ± 9.35	1337.91 ± 18.27	7.17

Table S2. Statistics of XANES LCF fits. Results of the XANES LCF showing the percentage contribution of different Mn species in gall tissues and healthy leaf (n=3 independent biological replicates from separately collected galls).

	Epidermis		Idioblasts		Nutritive t.		Parenchyma		Leaf	
Ligand	Mean	SE	Mean	SE	Mean	SE	Mean	SE	Mean	SE
thylakoids	0.2	0.2	0.0	0.0	0.0	0.0	0.0	0.0	4.2	4.2
Mn2mucilage	10.3	2.5	4.5	2.6	8.3	4.8	3.7	3.7	27.9	16.8
Mn2malate	9.7	5.9	0.0	0.0	8.8	3.7	0.0	0.0	4.9	4.9
Mn2aquo	17.0	7.8	47.9	14.5	2.9	2.9	21.9	13.4	8.4	8.4
Mn2phytate	61.4	7.0	47.5	10.2	65.9	5.3	74.3	12.3	32.9	17.4
Mn2GSH	0.0	0.0	0.0	0.0	3.9	3.0	0.0	0.0	17.6	8.8
Mn3acetate	0.0	0.0	0.0	0.0	9.9	5.7	0.0	0.0	0.9	0.9
Mn4oxide	1.5	1.3	0.1	0.1	0.3	0.3	0.1	0.1	3.3	1.4
