# Supplementary material for: How eriophyid mites shape metal metabolism in leaf galls on Tilia cordata
Source: New Phytol. 2025 Apr 16;246(5):2222–42. doi: 10.1111/nph.70103 (PMC12059528; doi:10.1111/nph.70103)
Supplement: Supplementary file 2 — Methods S1 Bulk element content in the plants and in the soil. Methods S2 Histochemical staining. Methods S3 Details of the measuring conditions for X‐ray spectroscopy. Methods S4 Quantifying micro‐XRF tomograms with absorption correction for nail galls as elliptical hollow objects. [file NPH-246-2222-s005.pdf]

New Phytologist

New Phytologist SupporWing InformaWion

Article title: How eriophyid mites shape metal metabolism in leaf galls on *Tilia cordata*

Authors: Filis Morina[1*], Anđela Kuvelja[1,2], Dennis Brückner[3], Miloš Mojović[4], Đura Nakarada[4], Syed Nadeem Hussain Bokhari[1], Bojan Vujić[1], Gerald Falkenberg[3], Hendrik Küpper[1,2*]

Article acceptance date: 06 March 2025

Method S1 Bulk element content in the plants and in the soil

For analyses of bulk element content, three types of samples, healthy leaves (HL), infested leaves (INFL), and nail galls (G) were pooled together from each of the six trees and freeze-dried. About 25-30 mg of freeze-dried tissues was acid digested in a mixture of 85% concentrated $HclO_4$ (Suprapur® grade, Carl Roth, Karlsruhe, Germany and 15% concentrated HNO_3 (Ultrapur® grade, Carl Roth, Karlsruhe, Germany) according to Zhao et al. (1994). Analyses of digested tissues were done as described in Andresen et al. (2020) with the sector field ICP-MS Element XR-2 with jet interface (Thermo Fisher Scientific, Bremen, Germany). Indium (1 ng/ml) was added to every sample as an internal standard. For analyses of extractable metal soil concentrations, the samples were extracted in 1M ammonium acetate pH7 (1:5 w/v) on a shaker for 1h as in Ure et al. (1993). The suspensions were centrifuged at 3000 rpm for 15 min at 25°C and the supernatant was filtered using 0.2 µm filters (Whatman, nucleopore). The same instrumentation used for the tissue digests was used for the soil samples as described above.

Method S2 Histochemical staining

For ruthenium red staining of mucilage, the apical and basal parts of the galls were cut off and the middle part was immersed in formalin-aceto-alcohol (FAA) fixative (90 mL of 50% ethanol, 5 mL of glacial acetic acid and 5 mL of 37% formaldehyde). After overnight fixation at 4°C, tissues were washed four times for 15 min in 50% ethanol. Standard histological techniques were used for tissue dehydration, embedding in paraplast, sectioning at 7 µm, deparaffinization and rehydration. Sections were washed with ddH2O and 0.1% ruthenium red was applied for 5 minutes. Stained sections were washed twice to remove the excess dye and mounted on glycerin or glycerin gelatin.

Method S3 Details of the measuring conditions for X-ray spectroscopy

For µXRF and µXANES, we used the µXRF beamline P06 at PETRAIII at DESY (Hamburg, Germany). Shock-frozen-hydrated samples were measured in the guided cryostream (see main text and Fig. 1 for details). The X-ray beam was generated in a 2m Spectroscopy undulator U32 and monochromatized using a cryogenically cooled Si(111) Double Crystal Monochromator at 15 keV with approximately 1.4x10-4 dE/E band pass. Focusing was achieved using Kirkpatrick-Baez (KB) mirrors. The beam was focused to about 0.5 x 0.5 µm size for µXRF tomography and 5 x 5 µm for µXANES tomography. For the latter, a radiation dose estimation was done based on measured average absorption in the 1.3 mm diameter galls (with central cavity as shown in the figures) around 80-85%, 1.0e10 ph/s incoming flux on the sample, 2.6 s scan time per line (10 ms per pixel), 180 rotation angles: 0.324 kGy per line, 874 kGy for the whole tomography. For detection, a Vortex Si--EM silicon drift detector and the Ardesia detector were used, one placed at 90° and the other one at 270° relative to the X-ray beam direction. Data reduction for XRF spectrum peak and background fitting was performed with PyMCA (Solé et al., 2007). Tomographic reconstruction was performed using a maximum-likelihood expectation-maximization algorithm (Bruyant, 2002).

For bulk XANES (samples HL1 and HL2 and part of the models), measurements were performed at the XAS beamline P65 at the synchrotron radiation source PETRA III (DESY, Hamburg, Germany). The beamline was equipped with a Si(331) monochromator. The beam size was 0.5 mm (vertical) *1.0 mm (horizontal). Samples were mounted in a He closed cycle cryostat and kept at temperatures arounf 20 K. Spectra were collected in fluorescence mode with a 4-element SDD detector (Hitachi ME4 SDD).

Method S4

Quantifying µXRF tomograms with absorption correction for nail galls as elliptical hollow objects

Simplifying assumptions:

(A) The gall is still close enough to a circle that deviations in absorption profile don't significantly deviate from slightly deforming an originally circular profile to the gall ellipse,
(B) The centre of the gall is filled mostly with air, in which the absorption of the elements measured is negligible within the size of the central cavity (max. 1 mm)

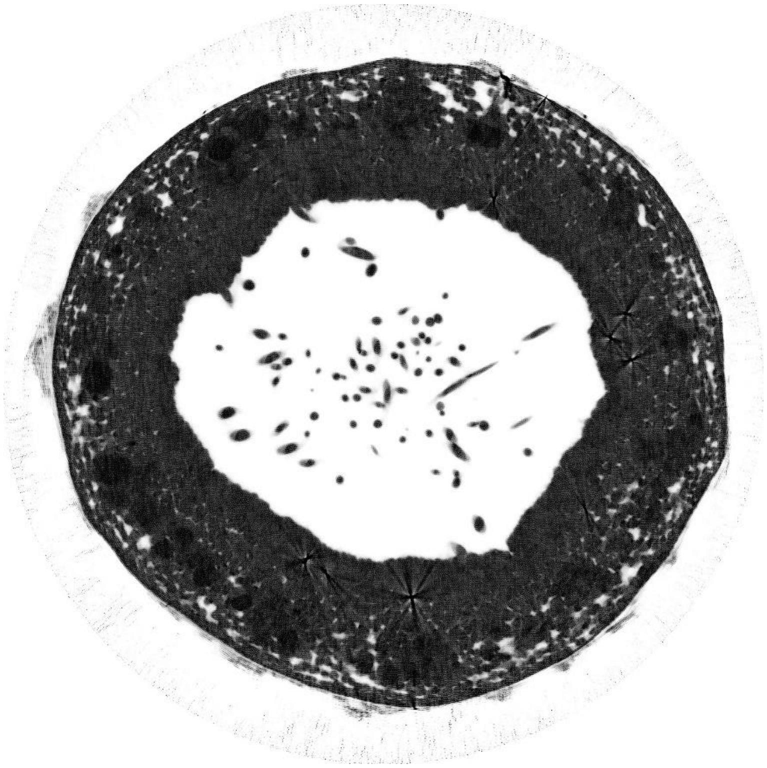

1) Data acquisition. In the beamline record a standard in a capillary, the diameter of which is approximately the same as the sum of the wall thickness in a transect through the gall, to have approximately equal absorption for both detectors that capture photons on both sides of the gall. The standard should have a composition as similar as possible to the sample, in the same measuring geometry and with the same flux as the sample. Do all data processing in 32-bit mode to minimise loss on dynamic range.

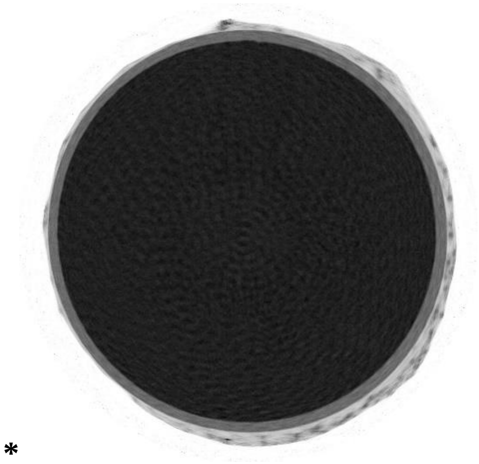

2) Export of absorption Gradient. In ImageJ, load the tomogram of a standard that is slightly bigger than the gall

a) For each element, determine the background outside the capillary, if it is >0 subtract that from the standard and all samples

b) For each element, create a radial profile on a selection that covers the **inner** diameter of the capillary

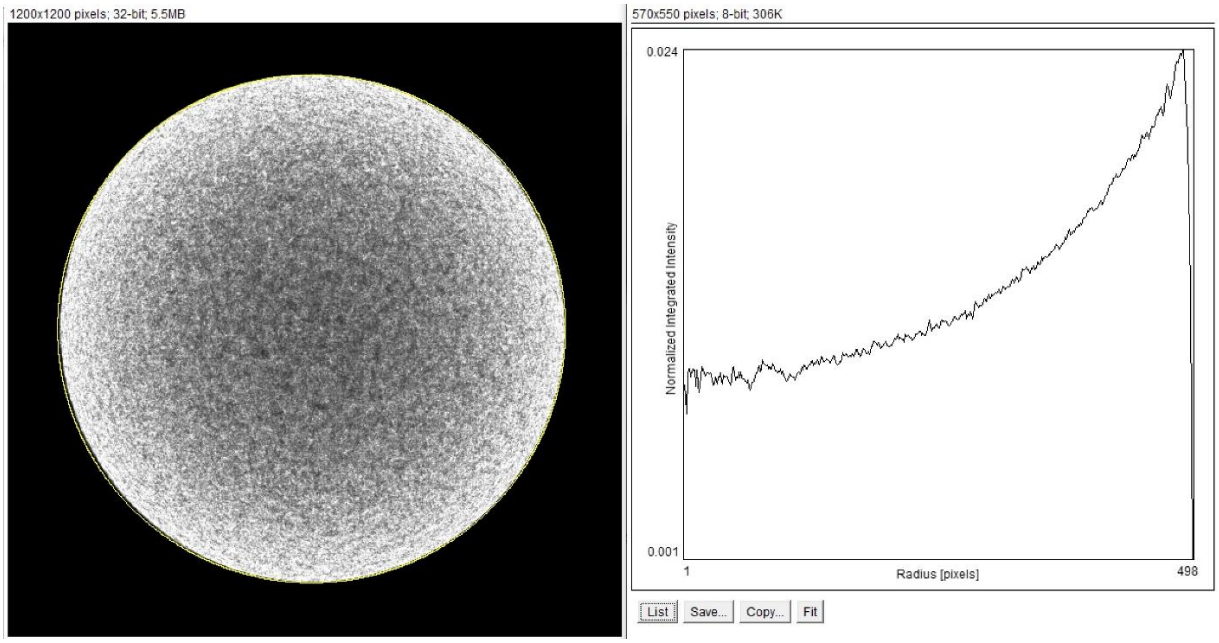

3) Fit of exponential. In Origin, fit an exponential decay, covering the whole X-axis corresponding to the wall thickness of the gall. Export this as greyscale gradient

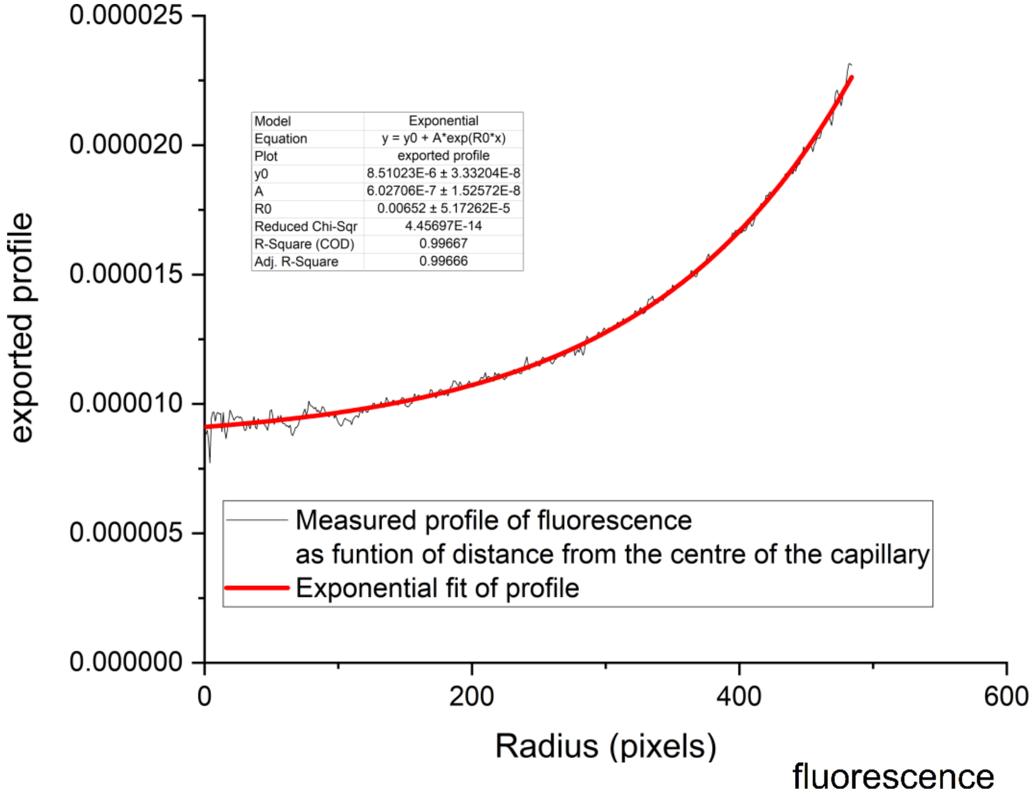

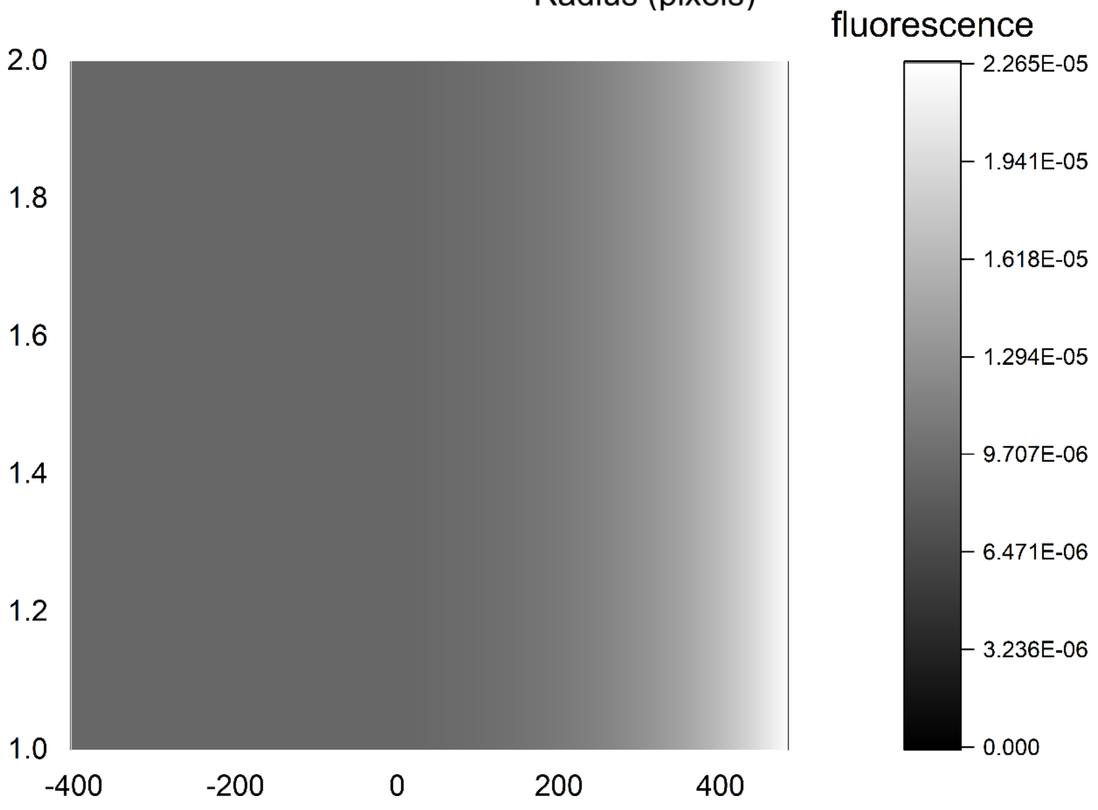

4) Polar transform of profile. In ImageJ, After cropping our the greyscale gradient to a strip 2 pixels high and with the width of the gall, run polar transformer in ImageJ, cartesian transform 360°. This will yield a circular map with the same pixel size like the tomogram of the capillary of the samples, with circular smoothed grey values.

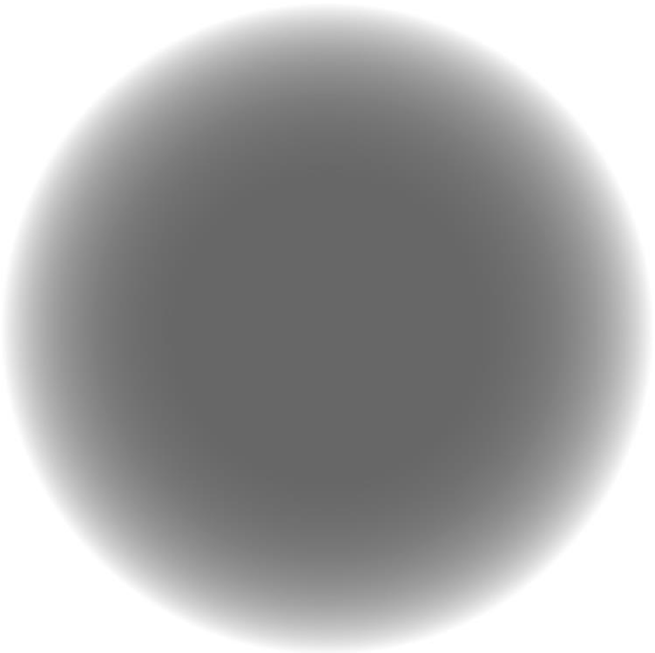

a) Determine size and position of the galls on the absorption tomogram by the selection tool (read the number of pixels on the top of the screen).

b) Then set size and border of the standard in the batch processing tool according to (a), resizing without “aspect ratio” and setting the borders with “add canvas”

6) Adaption of the standard to the gall shape (photo-editing software)

a) paste the absorption correction standard from step (5) onto the gall tomogram, make it 15% transparent. Areas where absorption would be overestimated because the correction standard protrudes beyond the tomogram look light. Areas where the absorption would be underestimated because the gall protrudes beyond the standard appear dark.

b) turn and distort the absorption correction standard until it ideally fits the gall tomogram. Be careful not to distort too much into one direction, because this will lead to big errors in the gradient for absorption correction.

7) Absorption correction of samples with simulated standards: In ImageJ

a) Divide the standard by its maximum value using the "image expression parser" in ImageJ. <Don't use the "math" function for it, because it does not work in a floating-point way, so that it leads only to integers, i.e. 0 and 1 in this case. Multiply with the maximal grey value of the original non-normalised standard. Save this as absorption-correction standard.

b) Divide the sample tomograms by the absorption-correction standard and multiply with the mM or ppm of the element that were contained in the standard to yield a ppm or mM scale in the sample tomogram.

8) Choice of concentration ranges and colour scales, export for publication

a) Determine and set the interesting concentration range for each element, as the best compromise for all samples to be compared

b) choose a colour scale for each element

c) disassemble stacks to individual images again, and re-label these images (they are in alphabetical order)

d) apply smoothing and/or binning for noise reduction, re-scaling to the original pixels/µm after binning

e) convert images to 24-bit RGB format suitable for publication.

References:

Andresen, E., Lyubenova, L., Hubáček, T., Bokhari, S.N.H., Matoušková, Š., Mijovilovich, A., Rohovec, J. and Küpper, H. (2020). Chronic exposure of soybean plants to nanomolar cadmium reveals specific additional high-affinity targets of cadmium toxicity. Journal of Experimental Botany, 71(4), pp.1628-1644.

Ure, A. M., Quevauviller, P. H., Muntau, H., Griepink, B. (1993). Speciation of heavy metals in soils and sediments. An account of the improvement and harmonization of extraction techniques undertaken under the auspices of the BCR of the Commission of the European Communities. International journal of environmental analytical chemistry, 51(1-4), 135-151.

Zhao, F., McGrath, S. P., Crosland, A. R. (1994). Comparison of three wet digestion methods for the determination of plant sulphur by inductively coupled plasma atomic emission spectroscopy (ICP-AES). Communications in soil science and plant analysis, 25(3-4), 407-418.
