# Supplementary material for: How eriophyid mites shape metal metabolism in leaf galls on Tilia cordata
Source: New Phytol. 2025 Apr 16;246(5):2222–42. doi: 10.1111/nph.70103 (PMC12059528; doi:10.1111/nph.70103)
Supplement: Supplementary file 3 — Notes S1 mRNA analyses. [file NPH-246-2222-s004.pdf]

*New Phytologist* Supporting Information

Article title: How eriophyid mites shape metal metabolism in leaf galls on *Tilia cordata*

Authors: Filis Morina[1*], Anđela Kuvelja[1,2], Dennis Brückner[3], Miloš Mojović[4], Đura Nakarada[5], Syed Nadeem Hussain Bokhari[1], Bojan Vujić[1], Gerald Falkenberg[3], Hendrik Küpper[1,2*]

Article acceptance date: 06 March 2025

Supporting Notes S1 mRNA analyses

## 1. Experimental Procedure

### 1.1 Sample quality control

Please refer to QC report for methods of sample quality control.

### 1.2 Library preparation for Transcriptome sequencing Non strand specific library

Messenger RNA was purified from total RNA using poly-T oligo-attached magnetic beads. After fragmentation, the first strand cDNA was synthesized using random hexamer primers followed by the second strand cDNA synthesis. The library was ready after end repair, A-tailing, adapter ligation, size selection, amplification, and purification.

The library was checked with Qubit and real-time PCR for quantification and bioanalyzer for size distribution detection.

**Strand specific library**

Messenger RNA was purified from total RNA using poly-T oligo-attached magnetic beads. After fragmentation, the first strand cDNA was synthesized using random hexamer primers. Then the second strand cDNA was synthesized using dUTP, instead of dTTP. The directional library was ready after end repair, A-tailing, adapter ligation, size selection, amplification, and purification.

The library was checked with Qubit and real-time PCR for quantification and bioanalyzer for size distribution detection.

### 1.3 Clustering and sequencing

After library quality control, different libraries were pooled based on the effective concentration and targeted data amount, then subjected to Illumina sequencing. The basic principle of sequencing is "Sequencing by Synthesis", where fluorescently labeled dNTPs, DNA polymerase, and adapter primers are added to the sequencing flow cell for amplification. As each sequencing cluster extends its complementary strand, the addition of each fluorescently labeled dNTP releases a corresponding fluorescence signal. The sequencer captures these fluorescence signals and converts them into sequencing peaks through computer software, thereby obtaining the sequence information of the target fragment.

## 2. Bioinformatics Analysis Pipeline

### 2.1 Data quality control

Raw data (raw reads) of fastq format were firstly processed through fastp software. In this step, clean data (clean reads) were obtained by removing reads containing adapter, reads containing ploy-N and low quality reads from raw data. At the same time, Q20, Q30 and GC content the clean data were calculated. All the downstream analyses were based on the clean data with high quality.

### 2.2 Transcriptome assembly

Trinity (v2.15.1) is an efficient and stable transcriptome splicing software for RNA-seq data developed in collaboration with the Hebrew University of Jerusalem and the Broad Institute. It combines three independent software modules to sequentially splice many RNA-seq data, namely: Inchworm, Chrysalis and Butterfly. The main process is as follows: Inchworm: First, read all the fq files of reads, then convert the fq reads files into fa format reads files, and merge the reads on end 3 and end 5 to get both. reads were then decomposed according to k-mers (short fragments of K-bp length), and the types of k-mers and the number of each type of k-mer were counted. Reads were sorted from highest to lowest according to the frequency of k-mer. The k-mer with the highest frequency is selected as the seed to start extending to end 3, extending one base each time. The number of occurrences of each k-mer after extension is counted, and the k-mer with the highest frequency is selected as the extension path. Finally, according to the greedy algorithm, The Overlap was used to extend k-mers and form contig sequence.

Chrysalis: All contigs with similar regions larger than k-1-mers were clustered to form components. de Bruijn graph was constructed according to different components.

reads were compared with components for verification.
Butterfly: butterfly transcripts were enumerated, the graph was split into linear sequences, the error sequences were eliminated using reads and pairs relations, the de Bruijn graph of each component was simplified, and the full-length transcript of the variable cut subtype was output.

After combing the transcripts corresponding to the parologous genes, the result file of the splicing was obtained: TRINITY.fasta.

### 2.3 Corset hierarchical clustering

Corset (Nadia M Davidson, Alicia Oshlack, 2014) aggregated transcripts into many clusters according to Shared Reads between transcripts on the basis of Trinity stitching. Combined with the expression level of transcripts between different samples and H-Cluster algorithm, transcripts with expression differences between samples are separated from the original cluster, and new clusters are established. Finally, each cluster is defined as "Gene". This method can polymerization redundant transcript, and improve the detection rate of differentially expressed genes, Corset's official website
https://code.google.com/p/corset-project/.

### 2.4 Transcript quality assessment

Benchmarking Universal Single-Copy Orthologs (BUSCO) is the evaluation of assembled transcripts using a Single Copy orthologous gene library combined with software such as tblastn, augustus, and hmmer. The integrity of transcript assembly was evaluated. We used BUSCO software to evaluate the splicing quality of Trinity.fasta, unigene.fa and cluster.fasta obtained by splicing, and evaluated the accuracy and completeness of the splicing results according to the proportion and completeness of the comparison.

## 2.5 Common function database annotation

Gene function was annotated based on the following databases:

Nr: The NCBI official protein sequence database includes the

protein-coding sequence of the GenBank gene, PDB(Protein DataBank) Protein database, SwissProt Protein sequence and data from PIR(Protein Information esource) and PRF(Protein Research Foundation) And other database of protein sequences.

Nt: NCBI official nucleic acid sequence database including the GenBank, EMBL or DDBJ (but not including EST, STS, GSS, WGS, TSA, PAT, HTG sequence) of nucleic acid sequences.

Pfam: The most comprehensive classification system for protein domain annotation Proteins are composed of individual domains, and the protein sequence of each specific domain has certain conserved properties. PFAM divides the protein domain into different protein families, and establishes the HMM statistical model of amino acid sequences of each family by comparing the protein sequences.

PFAM: The reliability of annotation results is divided into two main categories: the highly reliable PFAM-A family of manual annotation and the automatically generated PFAM-B family. Through the HMMER3 program, it is possible to search the HMM model of the established protein domain, and then annotate the protein family of the Gene.

KOG/COG: COG: Clusters of Orthologous Groups of proteins; KOG/COG: Cog: Clusters of orthologous groups of proteins; KOG: euKaryotic Ortholog Groups KOG and COG are both gene-based ortholog relationships of NCBI, with COG for prokaryotes and KOG for eukaryotes. The COG-KOG combined evolutionary relationship divides homologous genes from different species into different Ortholog clusters, currently 4,873 classes in COG and 4,852 classes in KOG. Genes from the same ortholog have the same function, so that functional annotations can be inherited directly to other members of the same COG/KOG cluster.

Swiss-Prot: A collection of protein sequences organized and studied by experienced biologists.

KEGG: A database that analyzes the metabolic pathways of gene products and compounds in cells and the functions of these gene products. It integrates data on genomes, chemical molecules, and biochemical systems, These include metabolic pathways (KEGG PATHWAY), KEGG drugs, KEGG diseases, functional models (KEGG MODULE), KEGG GENES, and KEGG GENOME. The KO(KEGG ORTHOLOG) system connects various KEGG annotation systems, and KEGG has established a complete KO annotation system for functional annotation of the genome or transcriptome of a newly sequenced species.

GO: An internationally standardized classification system for gene function description.

## 2.6 Reference sequence alignment

The transcriptome spliced by Trinity was used as a reference sequence (Ref). The clean reads of each sample were mapped to the Ref. reads with a comparison mass value lower than 10 were filtered out, and reads that were not paired were compared to reads in multiple regions of the genome. During the comparison, RSEM (v1.3.3) software was used (Li et al, 2011), and the bowtie2 parameter mismatch 0 was used in RSEM (the default parameter of bowtie2).

## 2.7 Differential expression analysis

For DESeq2 with biological replicates: Differential expression analysis for two conditions/groups was performed using the DESeq2 R package (1.42.0). DESeq2 provides statistical programs for determining differential expression in digital gene expression data using models based on negative binomial distribution. The resulting P-value is adjusted using the Benjamini and Hochberg’s methods to control the error discovery rate. The threshold of significant differential expression： padj <= 0.05 & |log2(foldchange)| >= 1.

For edgeR without biological replicates: Prior to differential gene expression analysis, for each sequencing library, read counts were adjusted using the edgeR R package (4.0.16) by scaling normalization factors to eliminate differences in sequencing depth between samples, followed by differential expression analysis. The resulting P value is adjusted using the Benjamini and Hochberg’s methods to control the error discovery rate. The threshold of significant differential expression： padj <= 0.005 & |log2(foldchange)| >= 1.

## 2.8 GO and KEGG enrichment analysis of differentially expressed genes

Gene Ontology (GO) enrichment analysis of differentially expressed genes was implemented by the GOseq R package, in which gene length bias was corrected. GO terms with corrected Pvalue less than 0.05 were considered significantly enriched by differential expressed genes.

KEGG is a database resource for understanding high-level functions and utilities of the biological system, such as the cell, the organism and the ecosystem, from molecular-level information, especially large-scale molecular datasets generated by genome sequencing and other high-throughput experimental technologies (http://www.genome.jp/kegg/). We used KOBAS software to test the statistical enrichment of differential expression genes in KEGG pathways.

## 2.9 PPI analysis of differentially expressed genes

PPI analysis of differentially expressed genes was based on the STRING database, which contains known and predicted protein-protein interactions. For species present in the database, we construct the network by extracting the target gene list from the database. Otherwise, we use diamond (version 0.9.13) to align the target gene sequences with selected reference protein sequences, and then establish the network based on the known interactions of the selected reference species.
