# Supplementary material for: How eriophyid mites shape metal metabolism in leaf galls on Tilia cordata
Source: New Phytol. 2025 Apr 16;246(5):2222–42. doi: 10.1111/nph.70103 (PMC12059528; doi:10.1111/nph.70103)
Supplement: Supplementary file 4 — Notes S2 Untargeted and targeted metabolomics. [file NPH-246-2222-s003.pdf]

New Phytologist

New Phytologist SupporWing InformaWion

Article title: How eriophyid mites shape metal metabolism in leaf galls on *Tilia cordata*
Authors: Filis Morina[1*], Anđela Kuvelja[1,2], Dennis Brückner[3], Miloš Mojović[4], Đura Nakarada[4], Syed Nadeem Hussain Bokhari[1], Bojan Vujić[1], Gerald Falkenberg[3], Hendrik Küpper[1,2*]
Article acceptance date: 06 March 2025

Notes S2 Untargeted and targeted metabolomics All samples analyses were carried out by MS-Omics (four biological replicates from each tissue type). Semi-polar metabolite analysis by LC-MS/MS was carried out using a Thermo Scientific Vanquish LC coupled to a Orbitrap Exploris 240 MS, Thermo Fisher Scientific. An electrospray ionization interface was used as ionization source. Analysis was performed in positive and negative ionization mode under polarity switching. The UPLC was performed using a slightly modified version of the protocol described by Catalin et al. (UPLC/MS Monitoring of Water-Soluble Vitamin Bs in Cell Culture Media in Minutes, Water Application note 2011, 720004042en). Peak areas were extracted using Compound Discoverer 3.3 (Thermo Scientific). Lipid analysis was carried out using a UHPLC system (Elute-XT system, Bruker Daltonics) coupled with a trapped ion-mobility high-resolution quadrupole-time-of flight mass spectrometer (timsTOF Pro 2, Bruker) equipped with VIP-HESI source). Ionization was performed in positive and negative ionization mode using an electrospray ionization interface. The chromatographic separation of lipids was carried out on a Waters® ACQUITY Charged Surface Hybrid (CSH™) C18 column (2.1 x 100 mm, 1.7 µm). The column was thermostated at 55°C. The mobile phases consisted of (A) Acetonitrile/water (60:40) and (B) Isopropanol/acetonitrile (90:10), both with 10 mM ammonium formate and 0.1% formic acid. Lipids were eluted in a two-step gradient by increasing B in A from 40 to 99% over 18 min. Flow rate was 0.4 ml/min. Peak areas were extracted using Metaboscape 2.2 (Bruker). For GC-metabolite analysis, the samples were derivatized with methyl chloroformate using a slightly modified version of the protocol described by Smart et al. (DOI: 10.1038/nprot.2010.108). All samples were analysed in a randomized order. Analysis was performed using gas chromatography (7890B, Agilent) coupled with a quadropole detector (5977B, Agilent). The system was controlled by ChemStation (Agilent). Raw data was converted to netCDF format using Chemstation (Agilent), before the data was imported and processed in Matlab R2021b (Mathworks, Inc.) using the PARADISe software described by Johnsen et. al (DOI: 10.1016/j.chroma.2017.04.052). Identification of compounds were performed at four levels; Level 1: identification by retention times (compared against in-house authentic standards), accurate mass (with an accepted deviation of 3ppm), and MS/MS spectra, Level 2a: identification by retention times (compared against in-house authentic standards), accurate mass (with an accepted deviation of 3ppm). Level 2b: identification by accurate mass (with an accepted deviation of 3ppm), and MS/MS spectra, Level 3: identification by accurate mass alone (with an accepted deviation of 3ppm).
